# Supplementary material for: Asymmetric small-molecule acceptor enables suppressed electron-vibration coupling and minimized driving force for organic solar cells
Source: Nat Commun. 2025 Feb 10;16:1503. doi: 10.1038/s41467-025-56799-6 (PMC11811148; doi:10.1038/s41467-025-56799-6)
Supplement: Supplementary file 1 — Supplementary Information [file 41467_2025_56799_MOESM1_ESM.pdf]

Supplementary Information

Asymmetric Small-Molecule Acceptor Enables Suppressed Electron-Vibration Coupling and Minimized Driving Force for Organic Solar Cells

Jing Guo[1*], *Shucheng Qin*[2], *Jinyuan Zhang*[2], *Can Zhu*[2], *Xinxin Xia*[3], *Yufei Gong*[2], *Tongling Liang*[4], *Yan Zeng*[2], *Guangchao Han*[2], *Hongmei Zhuo*[2], *Yuechen Li*[2], *Lei Meng*[2], *Yuanping Yi*[2], *Jianhui Chen*[1*], *Xiaojun Li*[2*], *Beibei Qiu*[5*] *and Yongfang Li*[2, 6]

1. Province-Ministry Co-construction Collaborative Innovation Center of Hebei Photovoltaic Technology, Hebei Key Laboratory of Optic-electronic Information and Materials, College of Physics Science and Technology, Hebei University, Baoding, Hebei 071002, China

2. Beijing National Laboratory for Molecular Sciences, CAS Key Laboratory of Organic Solids, Institute of Chemistry Chinese Academy of Sciences, Beijing 100190, China

3. National Engineering Research Center for Colloidal Materials, Shandong University, Jinan, Shandong 250100, China

4. Center for Physicochemical Analysis and Measurement, Institute of Chemistry, Chinese Academy of Sciences, Beijing 100190, China.

5. Key Laboratory of Solid-State Optoelectronic Devices of Zhejiang Province, College of Physics and Electronic Information Engineering, Zhejiang Normal University, Jinhua, Zhejiang 321004, China

6. Laboratory of Advanced Optoelectronic Materials, Suzhou Key Laboratory of Novel Semiconductor-optoelectronics Materials and Devices, College of Chemistry, Chemical Engineering and Materials Science, Soochow University, Suzhou, Jiangsu 215123, China

* **Corresponding authors**: guojing@hbu.edu.cn (J. Guo), chenjianhui@hbu.edu.cn (J. Chen), lixiaojun@iccas.ac.cn (X. Li), and qiubeibei@zjnu.edu.cn (B. Qiu)

Supplementary Figures

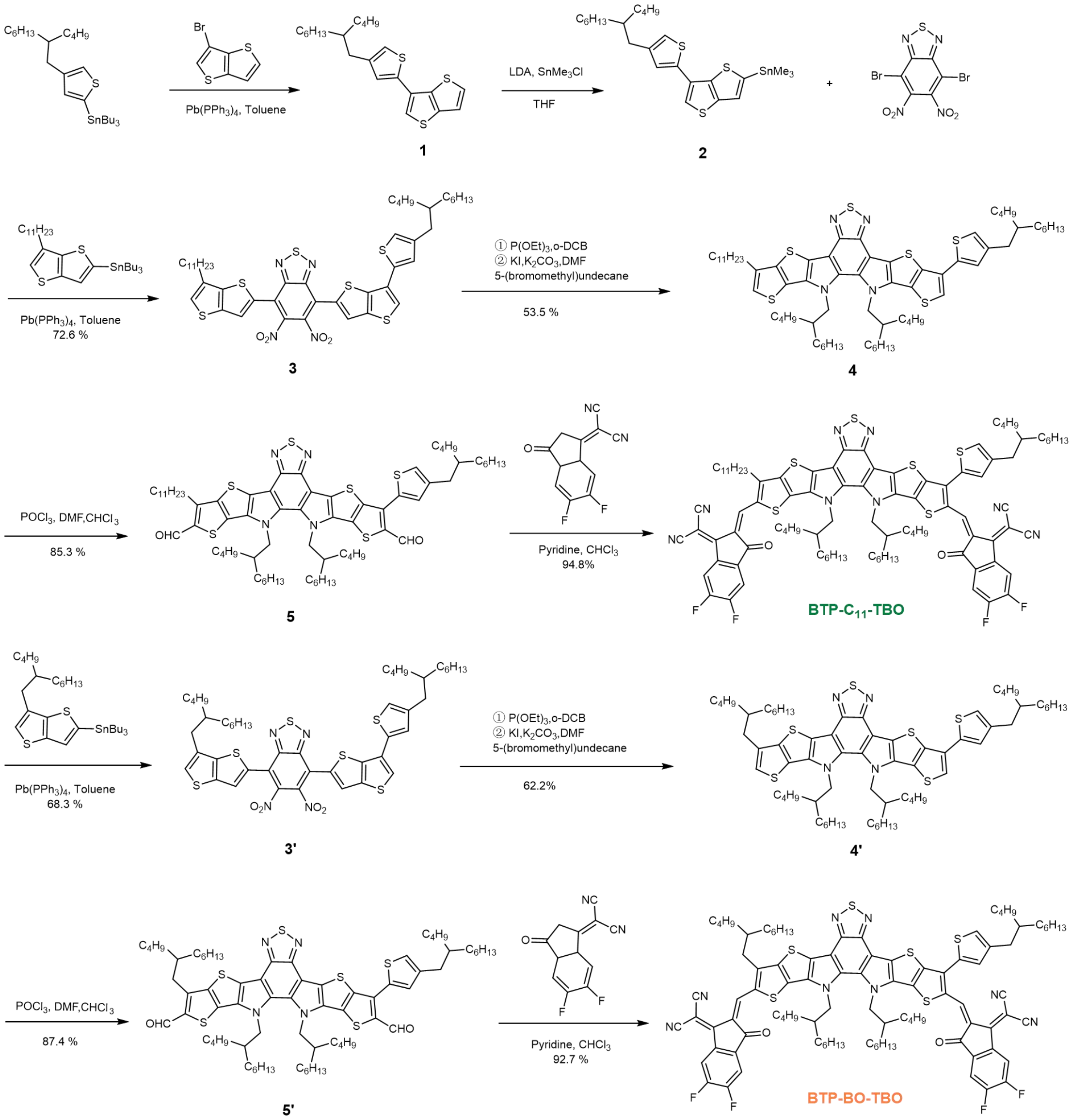

Supplementary Fig. 1 | Acceptors molecule synthesis. The synthetic routes of 1D/2D side chain asymmetric acceptors (BTP-C11-TBO and BTP-BO-TBO).

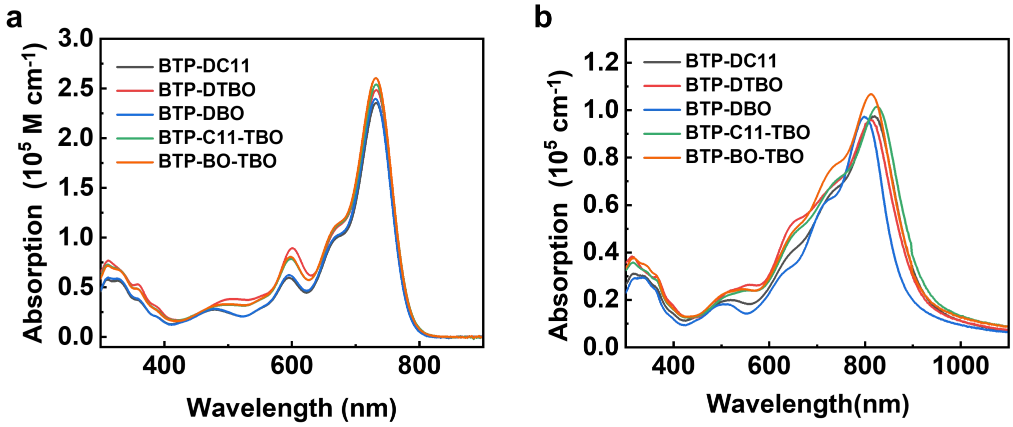

Supplementary Fig. 2 | Absorption spectra. a Absorption spectra and of BTP-DC11, BTP-DTBO, BTP-DBO, BTP-C11-TBO and BTP-BO-TBO in dilute chloroform solution; **b** Absorption spectra of BTP-DC11, BTP-DTBO, BTP-DBO, BTP-C11-TBO and BTP-BO-TBO in solid state. Source data are provided as a Source Data file.

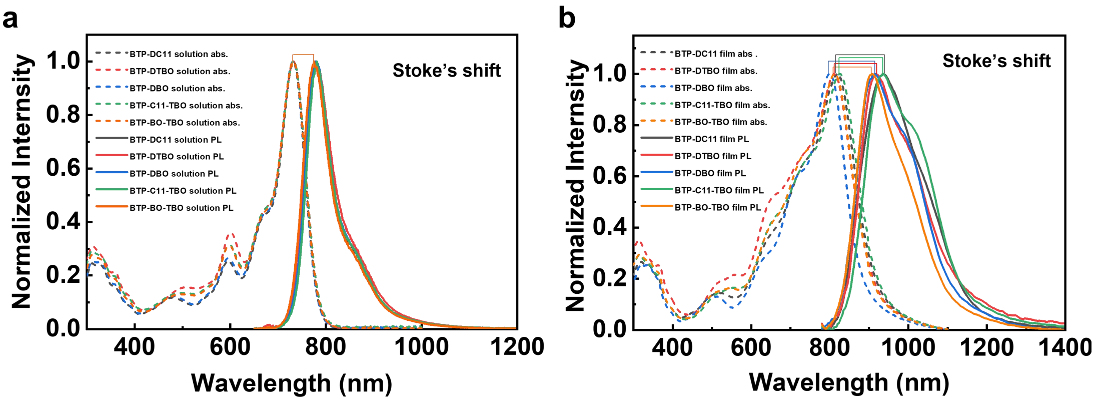

Supplementary Fig. 3 | Photoluminescence spectrum. a Normalized PL spectra and Stokes shifts of five acceptors in chloroform solution. **b** Normalized PL spectra and Stokes shifts of five acceptors in films. Source data are provided as a Source Data file.

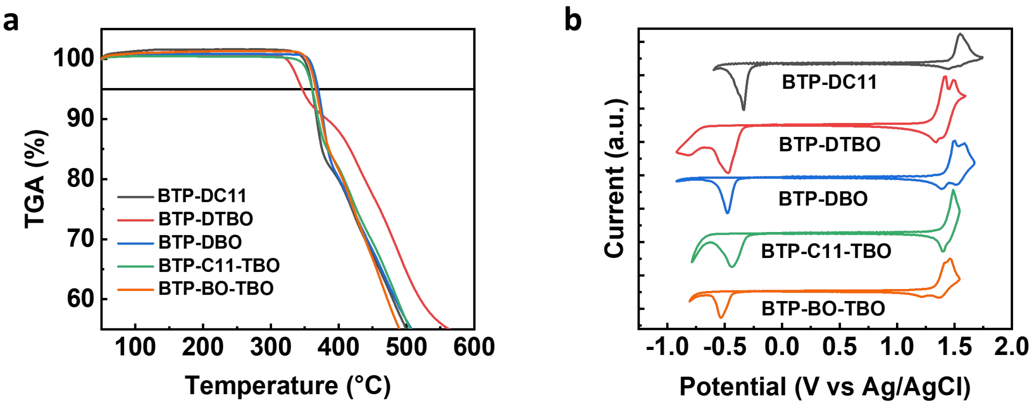

Supplementary Fig. 4 | Thermogravimetric analysis and cyclic voltammograms curves. TGA plots (**a**) and the cyclic voltammograms (**b**) of the BTP-DC11, BTP-DTBO, BTP-DBO, BTP-C11-TBO and BTP-BO-TBO.

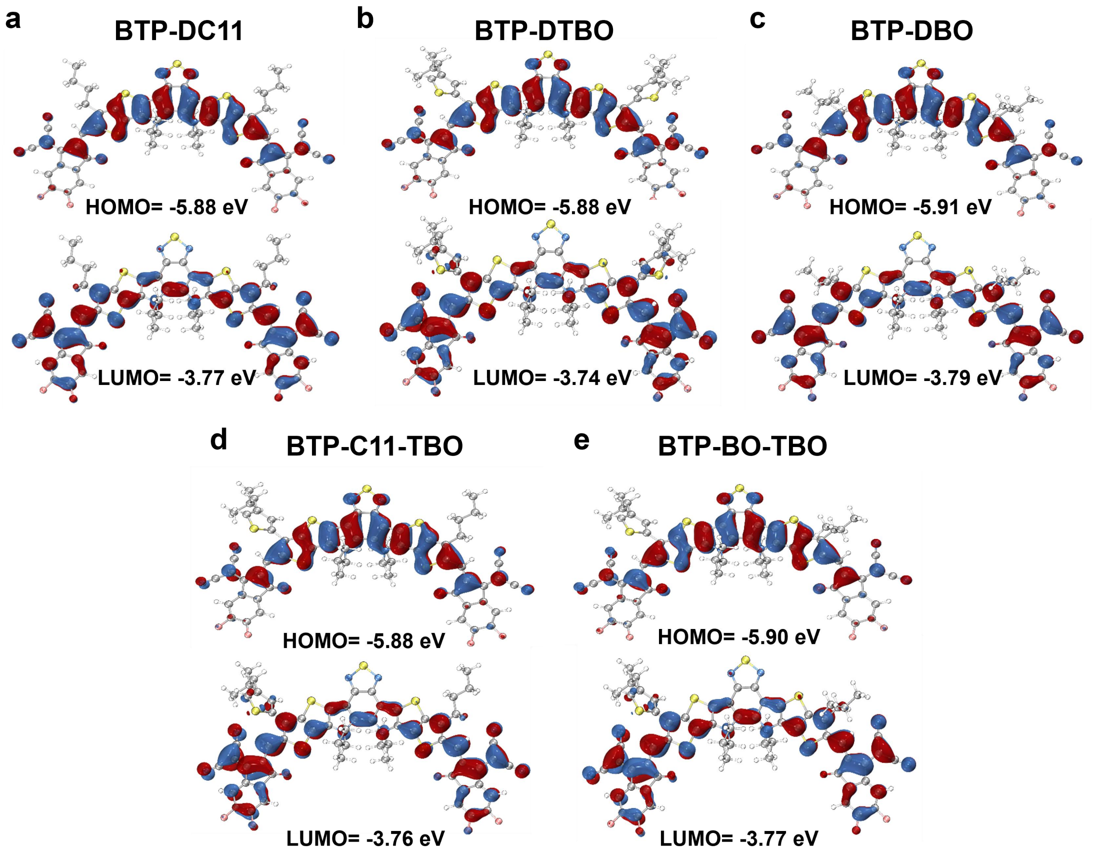

Supplementary Fig. 5 | The HOMO/LUMO energy levels by DFT at the B3LYP/6-31G (d,p) level. BTP-DC11 (**a**), BTP-DTBO (**b**), **c** BTP-DBO (**c**), BTP-C11-TBO (**d**) and **e** BTP-BO-TBO (**e**).

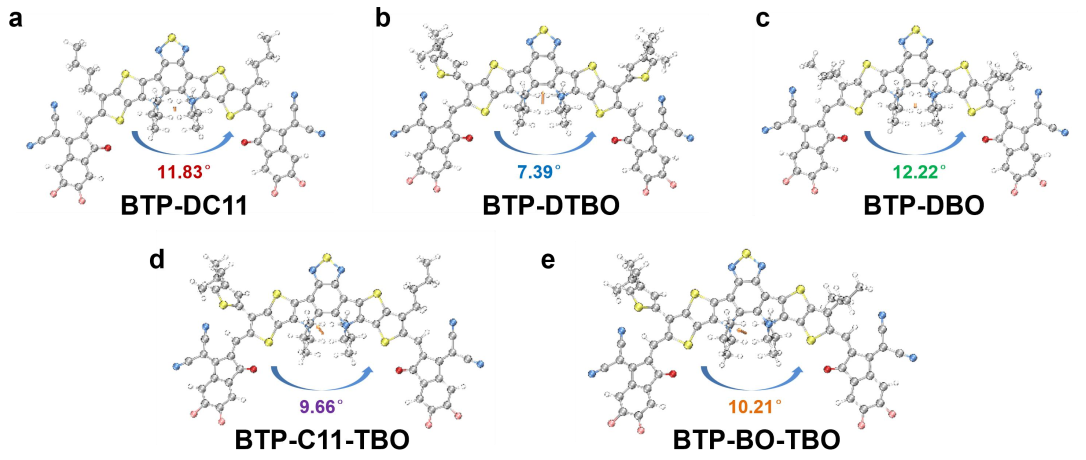

Supplementary Fig. 6 | The dihedral angles of SMAs. BTP-DC11 (**a**), BTP-DTBO (**b**), BTP-DBO (**c**), BTP-C11-TBO (**d**) and BTP-BO-TBO (**e**).

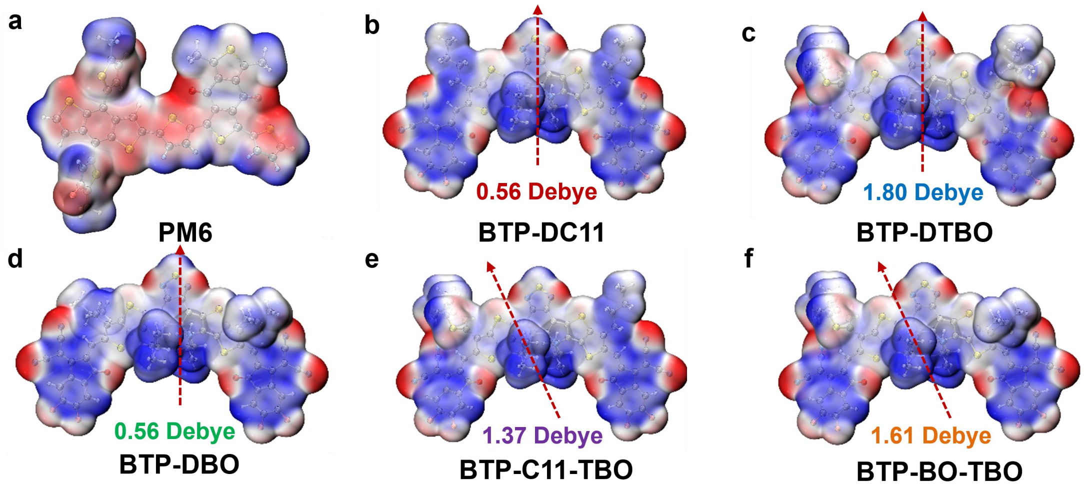

Supplementary Fig. 7 | The dipole moments of molecules. PM6 (**a**), BTP-DC11 (**b**), BTP-DTBO (**c**), BTP-DBO (**d**), BTP-C11-TBO (**e**) and BTP-BO-TBO (**f**).

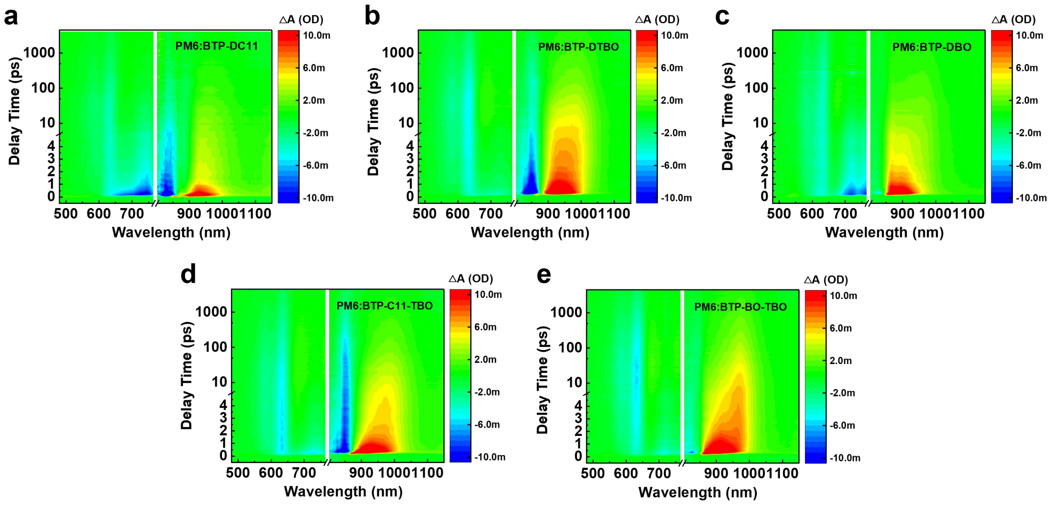

Supplementary Fig. 8 | 2D transient absorption spectra of in the blend films. PM6:BTP-DC11 (**a**), PM6:BTP-DTBO (**b**), PM6:BTP-DBO (**c**), PM6:BTP-C11-TBO (**d**) and PM6:BTP-BO-TBO (**e**).

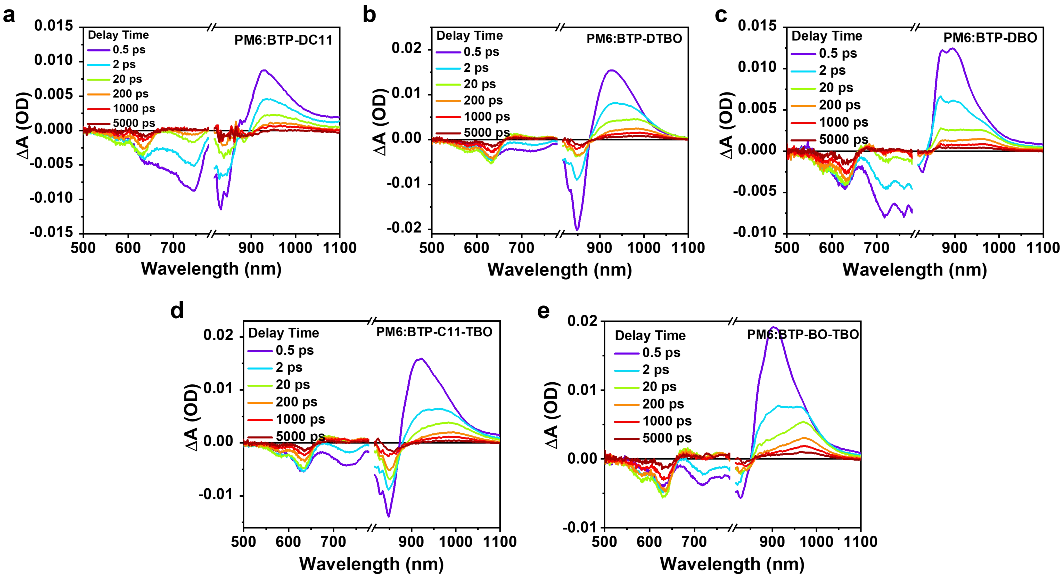

Supplementary Fig. 9 | Femtosecond transient absorption spectra of blend films. PM6:BTP-DC11 (**a**), PM6:BTP-DTBO (**b**), PM6:BTP-DBO (**c**), PM6:BTP-C11-TBO (**d**) and PM6:BTP-BO-TBO (**e**).

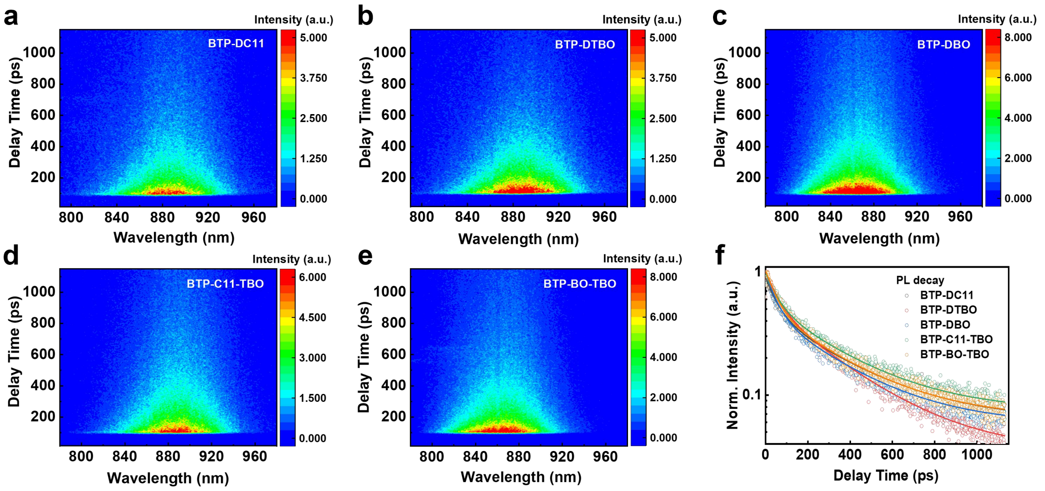

Supplementary Fig. 10 | Streak camera images of the photoluminescence. BTP-DC11 (**a**), BTP-DTBO (**b**), BTP-DBO (**c**), BTP-C11-TBO (**d**) and BTP-BO-TBO (**e**) pristine acceptor films; **f** Photoluminescence decay traces of the pristine acceptor films.

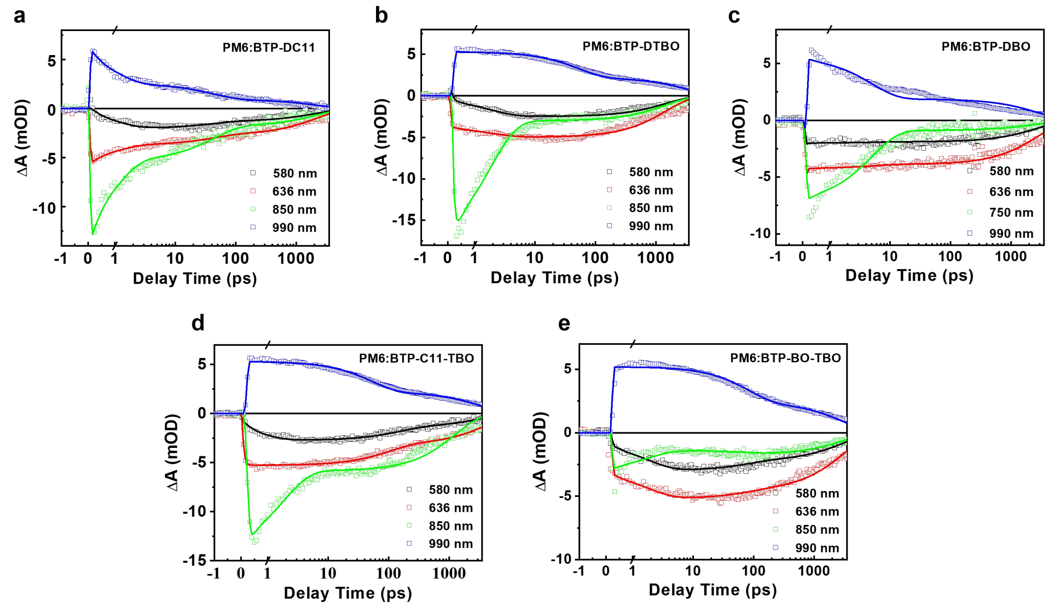

Supplementary Fig. 11 | Global fitting of kinetic traces. PM6:BTP-DC11(**a**), PM6:BTP-DTBO (**b**), PM6:BTP-DBO (**c**), PM6:BTP-C11-TBO (**d**) and PM6:BTP-BO-TBO (**e**) blend films.

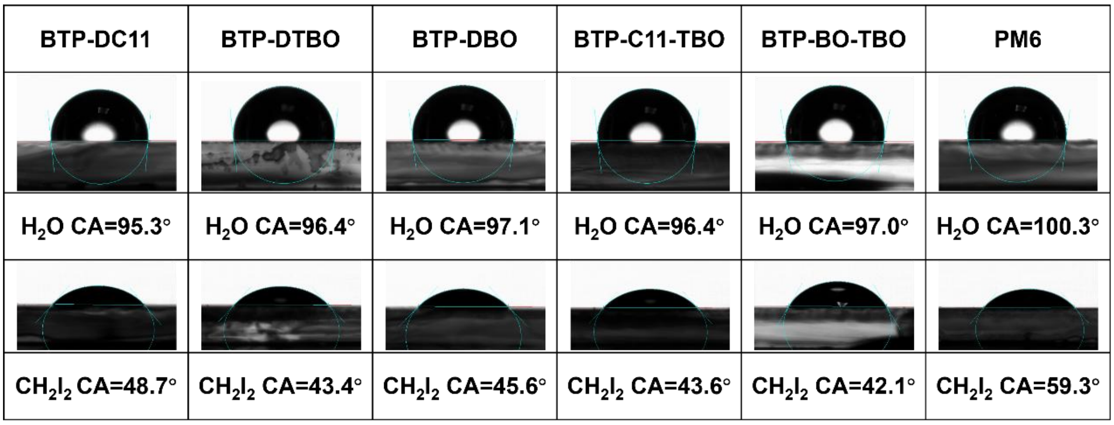

Supplementary Fig. 12 | The contact angle images of BTP-DC11, BTP-DTBO, BTP-DBO, BTP-C11-TBO and BTP-BO-TBO Film.

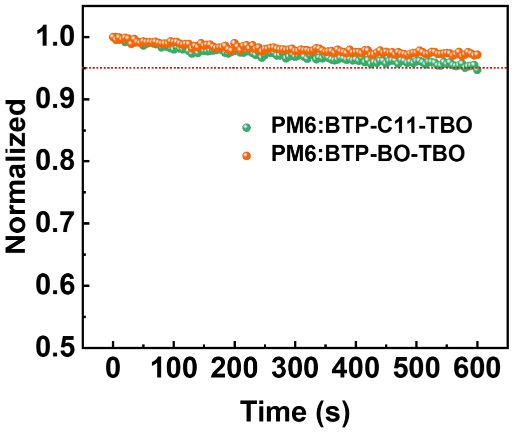

Supplementary Fig. 13 | The maximum power point tracking (MPPT) stability of OSCs. The OSCs based on the PM6: BTP-C11-TBO and PM6: BTP-BO-TBO devices. Source data are provided as a Source Data file.

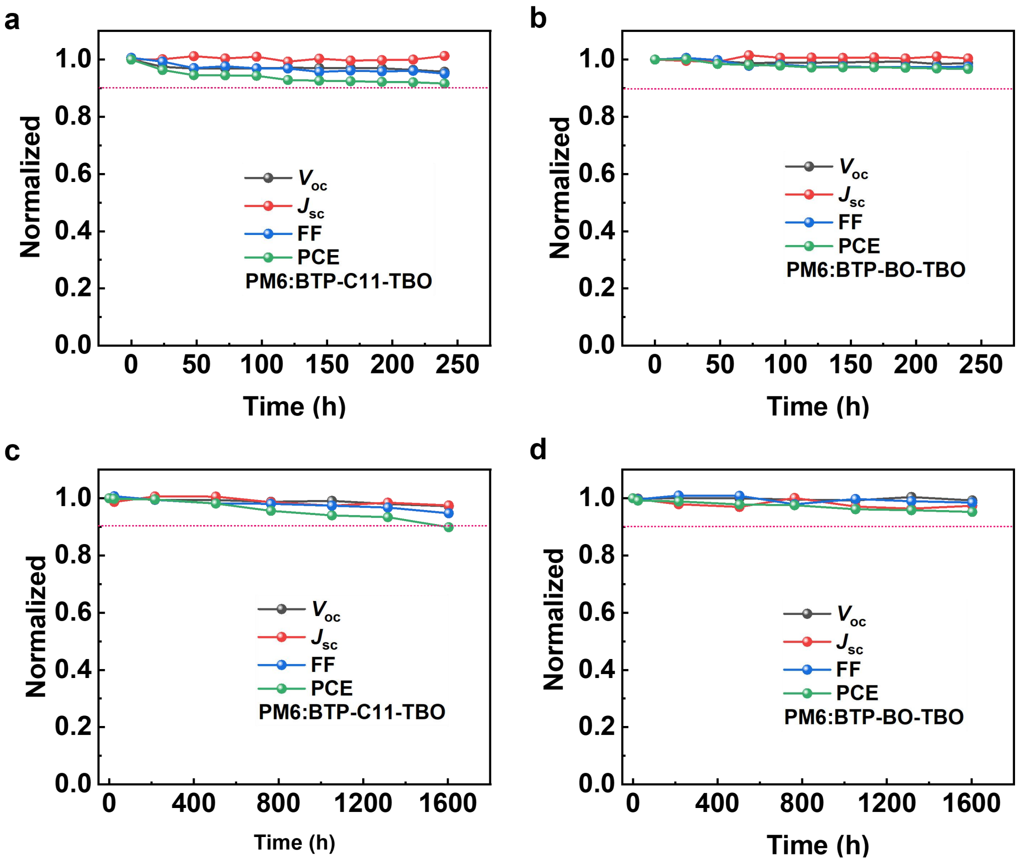

Supplementary Fig. 14 | The stability of OSCs. The operational stability of OSCs based on the PM6: BTP-C11-TBO (**a**) and PM6: BTP-BO-TBO devices (**b**). The storage stability of OSCs based on the PM6: BTP-C11-TBO (**c**) and PM6: BTP-BO-TBO devices (**d**). Source data are provided as a Source Data file.

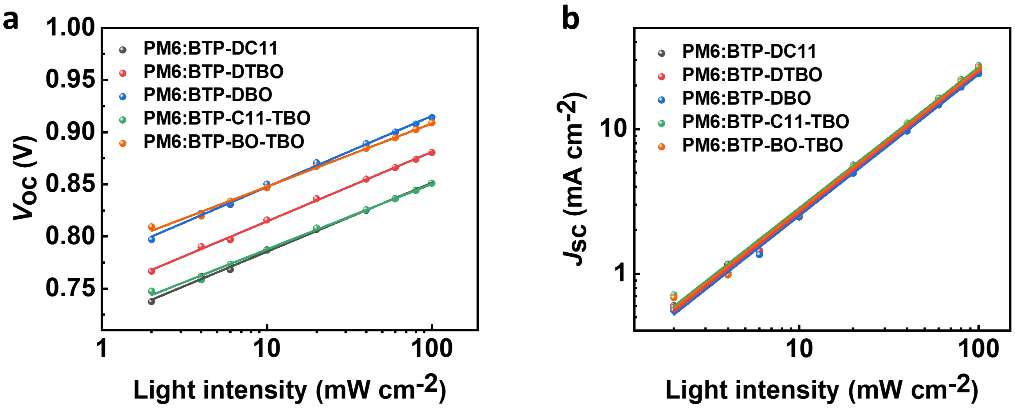

Supplementary Fig. 15 | The V_{oc} versus ln (light intensities) (P_{light}) (a) and log J_{sc} versus logP_{light} (b) plots of OSCs based on PM6:BTP-DC11; PM6:BTP-DTBO; PM6:BTP-DBO; BTP-C11-TBO and BTP-BO-TBO.

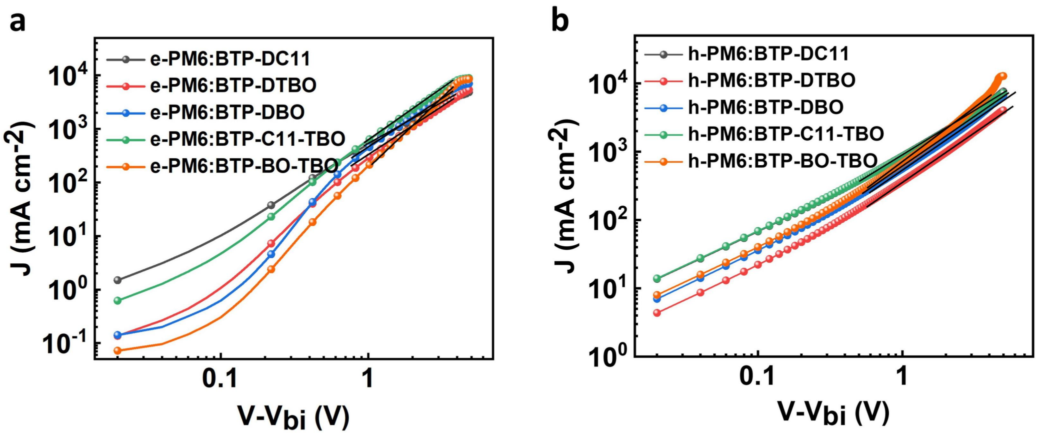

Supplementary Fig. 16 | The mobilities of OSCs. a *J-V* curves of electron-only devices. **b** *J-V* curves of hole-only devices.

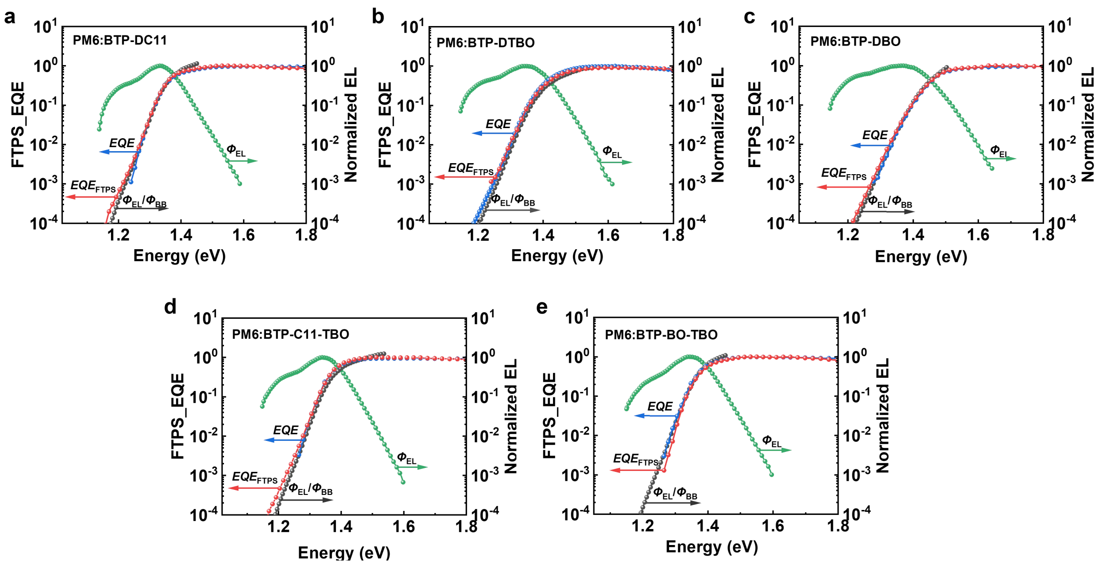

Supplementary Fig. 17 | Semi-logarithmic plots of the EL, sensitive EQE and reciprocally calculated EL and EQE as a function of energy. PM6:BTP-DC11 (**a**); PM6:BTP-TBO (**b**); PM6:BTP-DBO (**c**); PM6:BTP-C11-TBO (**d**) and PM6:BTP-BO-TBO (**e**).

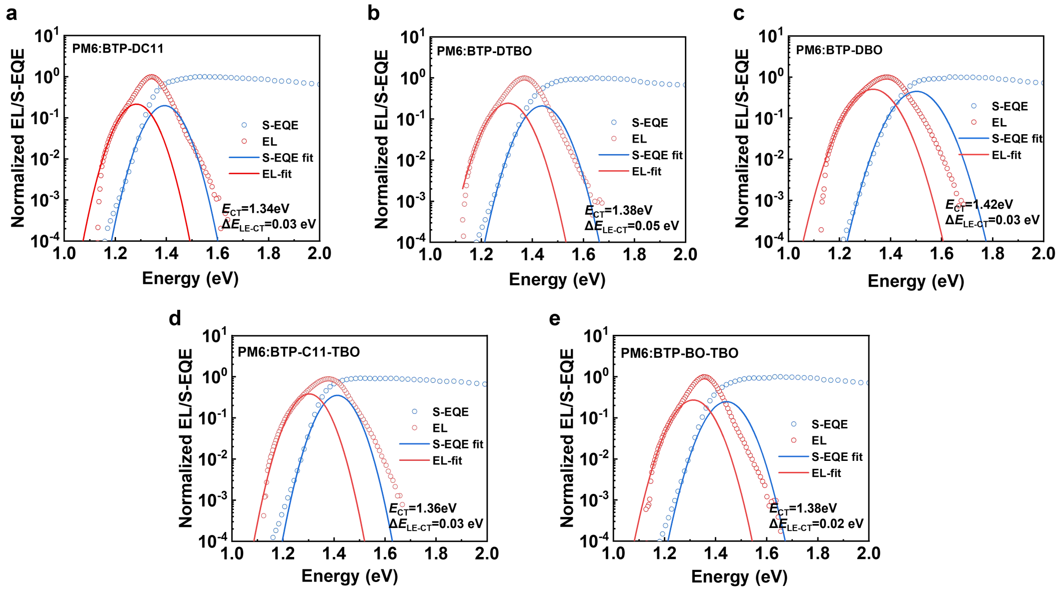

Supplementary Fig. 18 | The E_{CT} and ΔE_{LE-CT}. The s-EQE and EL curves of the devices based on PM6:BTP-DC11 (**a**); PM6:BTP-DTBO (**b**); PM6:BTP-DBO (**c**); PM6:BTP-C11-TBO (**d**) and PM6:BTP-BO-TBO (**e**).

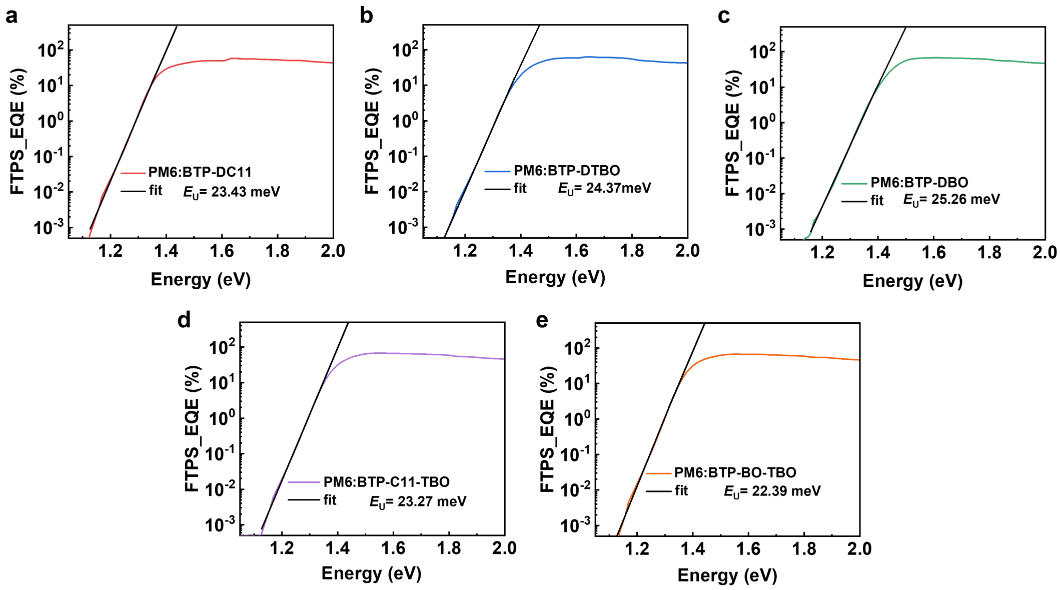

Supplementary Fig. 19 | The E_U of OSCs. FTPS-EQE of the devices based on PM6:BTP-DC11(**a**); PM6:BTP-DTBO (**b**); PM6:BTP-DBO (**c**); PM6:BTP-C11-TBO (**d**) and PM6:BTP-BO-TBO (**e**) at the absorption onset.

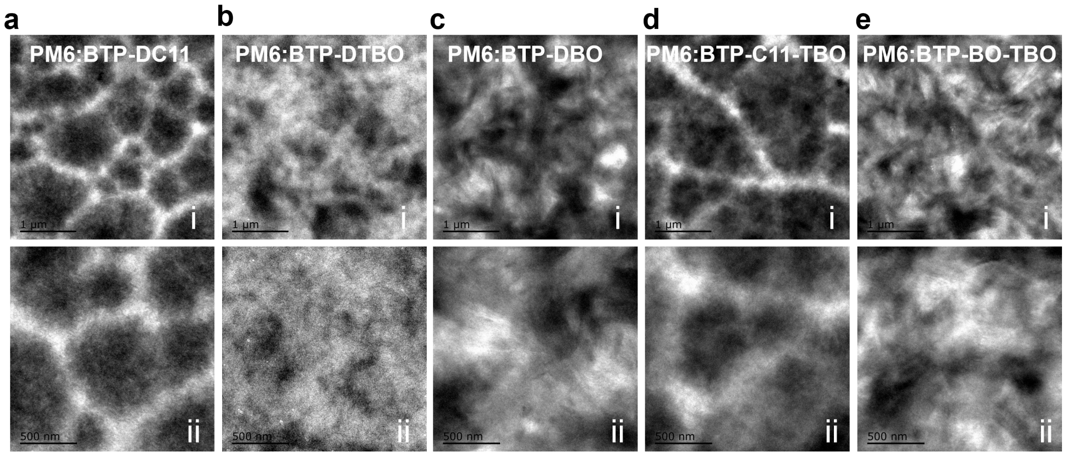

Supplementary Fig. 20 | The TEM phase images (1 μm (i) and 500 nm (ii) scale) of the blend films. PM6: BTP-DC11 (**a**); PM6: BTP-DTBO (**b**); PM6: BTP-DBO (**c**); PM6: BTP-C11-TBO (**d**); PM6: BTP-BO-TBO (**e**).

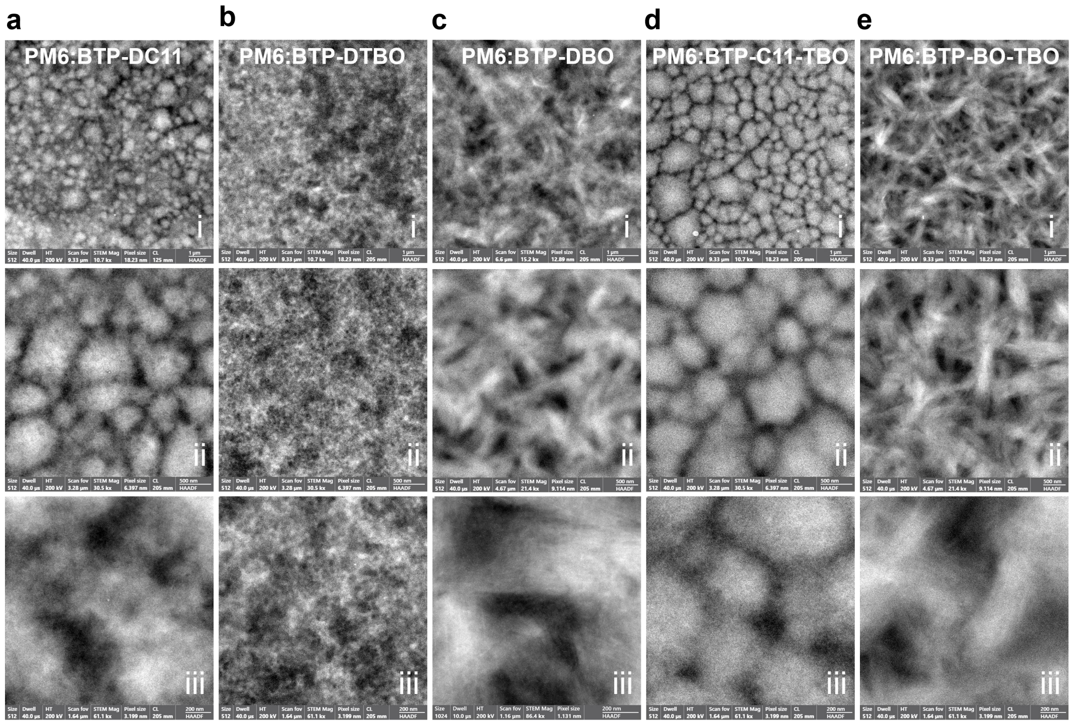

Supplementary Fig. 21 | The STEM phase images (1 μm (i), 500 nm (ii) and 200 nm (iii) scale) of the blend films. PM6: BTP-DC11 (**a**); PM6: BTP-DTBO (**b**); PM6: BTP-DBO (**c**); PM6: BTP-C11-TBO (**d**); PM6: BTP-BO-TBO (**e**).

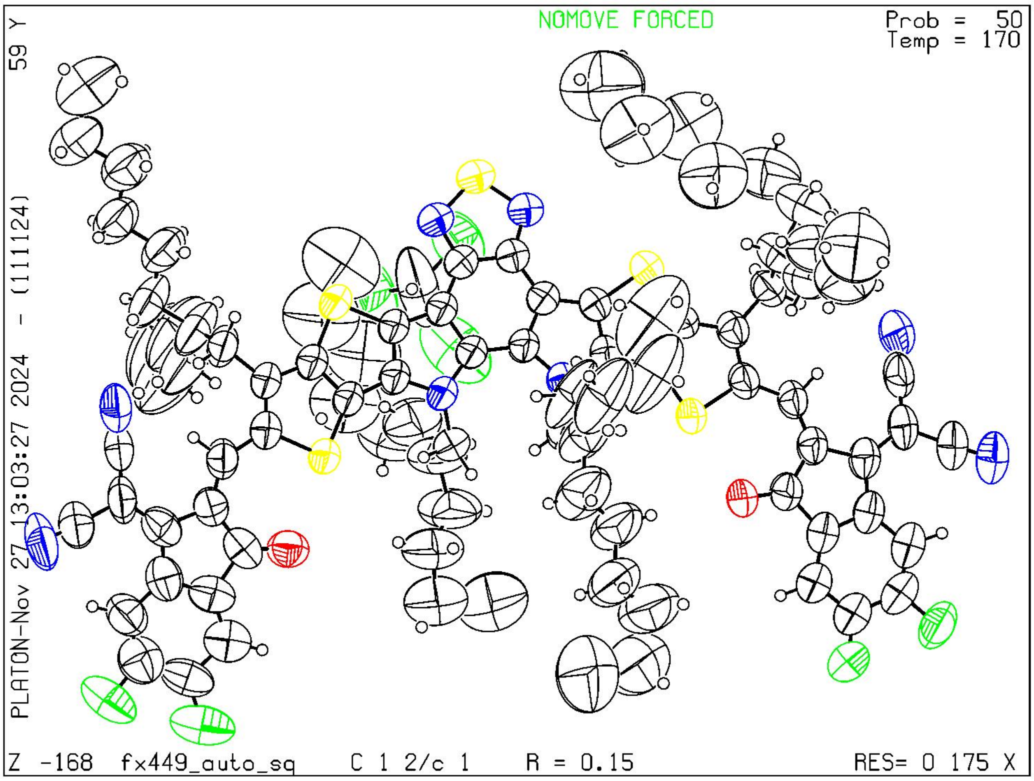

Supplementary Fig. 22 | The Oak Ridge Thermal Ellipsoid Plot (ORTEP) view of BTP-DBO (CCDC number: 2405808) with atomic displacement parameters shown at the 50% probability level. Alerts justification: Numerous crystals obtained from various methods have been attempted, but the quality is still unsatisfactory, which can be attributed to the high degree of disorder caused by outer/inner alkyl chains and the crystal decomposition during the measurement process. Despite the long exposure time, the crystal did not exhibit observable intensity at a resolution above 0.90 angstrom, therefore level B alarms were displayed in the Check CIF report. However, in this work, we only analyzed based on the conjugated skeleton and thiophene side chain skeleton.

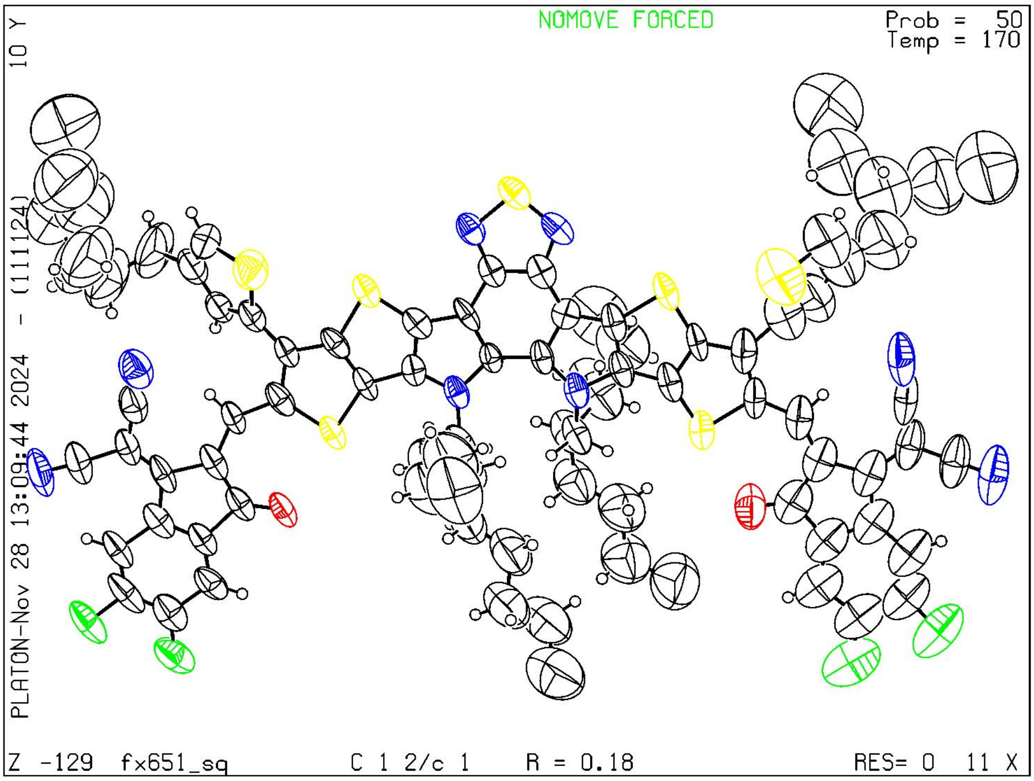

Supplementary Fig. 23 | The Oak Ridge Thermal Ellipsoid Plot (ORTEP) view of BTP-DTBO (CCDC number: 2405811) with atomic displacement parameters shown at the 50% probability level. Alerts justification: Numerous crystals obtained from various methods have been attempted, but the quality is still unsatisfactory, which can be attributed to the high degree of disorder caused by outer/inner alkyl chains and the crystal decomposition during the measurement process. Despite the long exposure time, the crystal did not exhibit observable intensity at a resolution above 0.90 angstrom, therefore level B alarms were displayed in the Check CIF report. However, in this work, we only analyzed based on the conjugated skeleton and thiophene side chain skeleton.

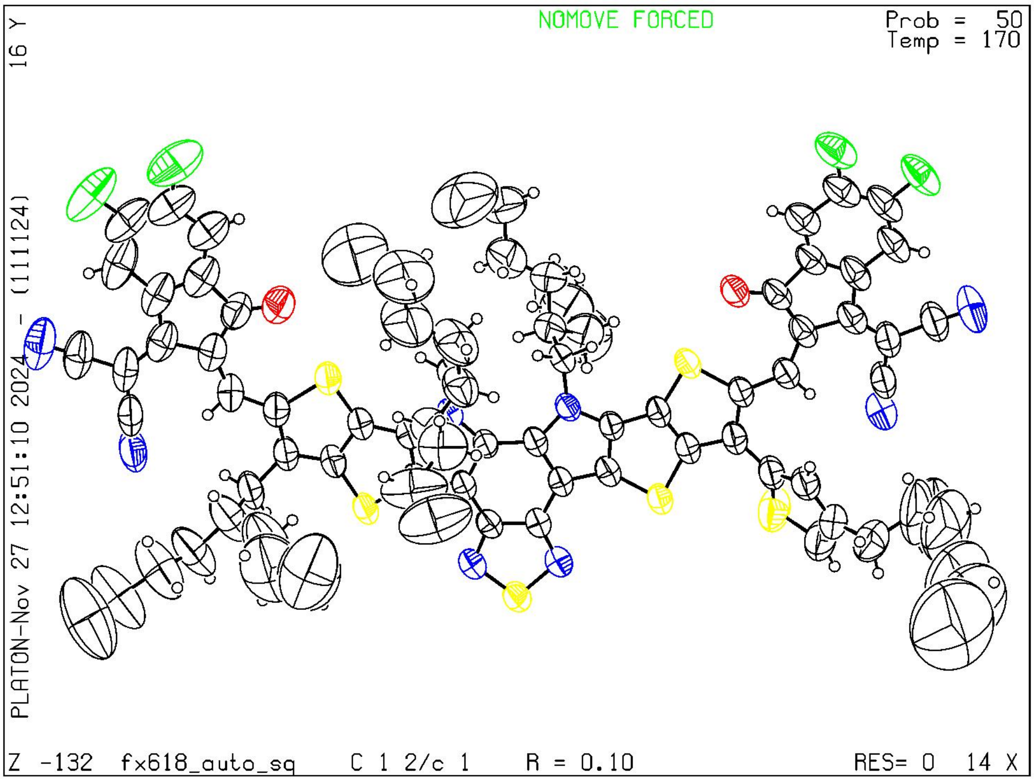

Supplementary Fig. 24 | The Oak Ridge Thermal Ellipsoid Plot (ORTEP) view of BTP-BO-TBO (CCDC number: 2405812) with atomic displacement parameters shown at the 50% probability level. Alerts justification: Numerous crystals obtained from various methods have been attempted, but the quality is still unsatisfactory, which can be attributed to the high degree of disorder caused by outer/inner alkyl chains and the crystal decomposition during the measurement process. Despite the long exposure time, the crystal did not exhibit observable intensity at a resolution above 0.90 angstrom, therefore level B alarms were displayed in the Check CIF report. However, in this work, we only analyzed based on the conjugated skeleton and thiophene side chain skeleton.

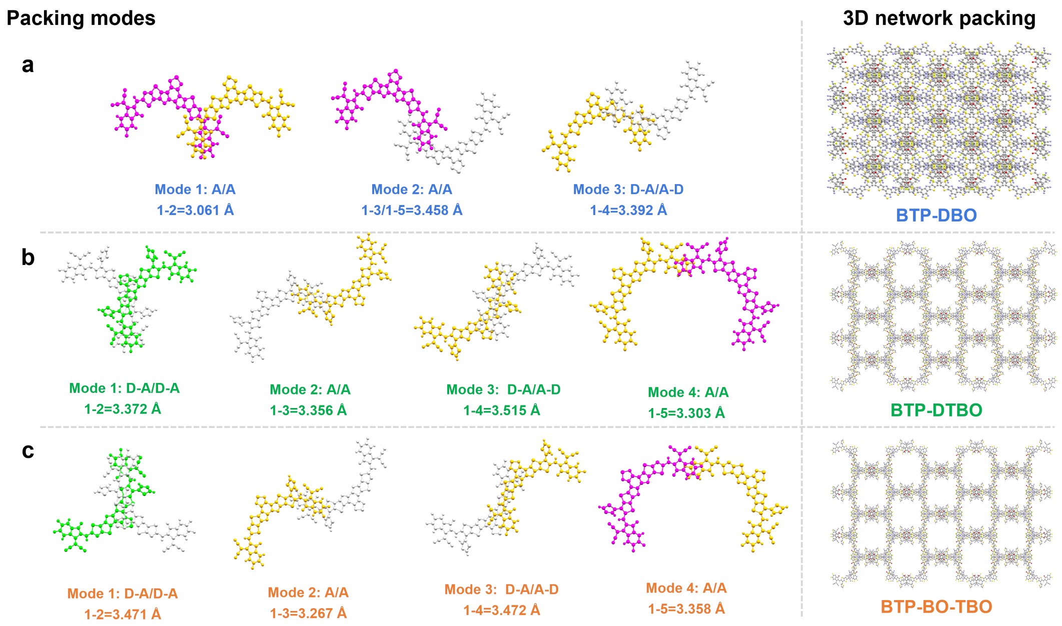

Supplementary Fig. 25 | Single crystal diffraction results. Crystallographic structures and their intermolecular packing modes and the 3D network packing of BTP-DBO (**a**), BTP-DTBO (**b**) and BTP-BO-TBO (**c**) along the c-axis.

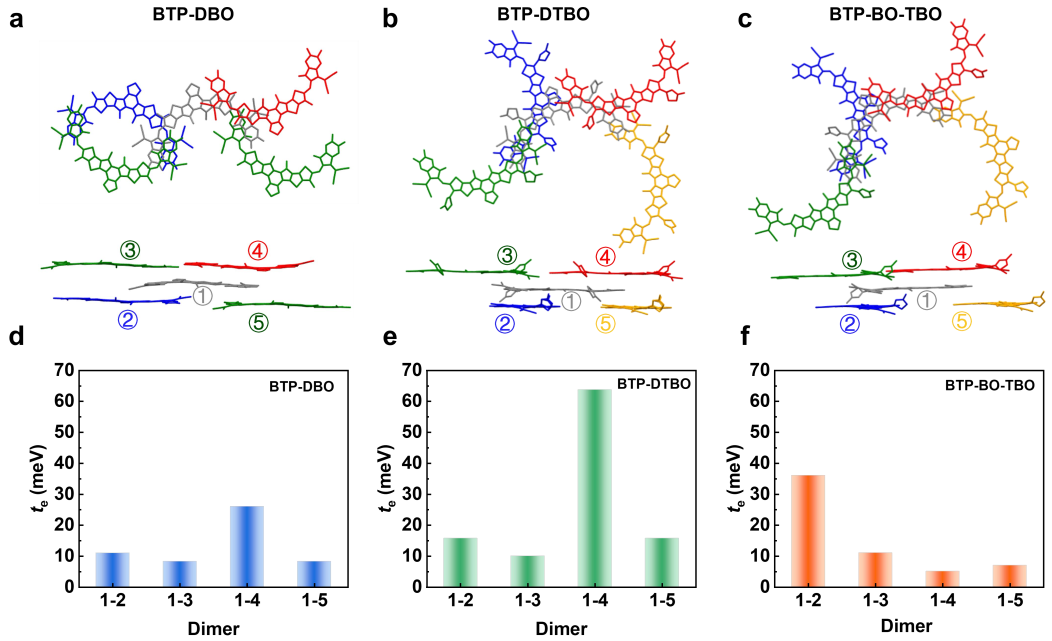

Supplementary Fig. 26 | Theoretical calculation based on single crystallographic structures. a-c Illustration of the nearest-neighbor molecular pairs and the corresponding electron transfer integrals calculated at the B3LYP/6-31G** level for BTP-DBO (**d**), BTP-DTBO (**e**), and BTP-BO-TBO (**f**) (For clarity, the alkyl side chains are shortened by methyl groups and the hydrogen atoms are omitted).

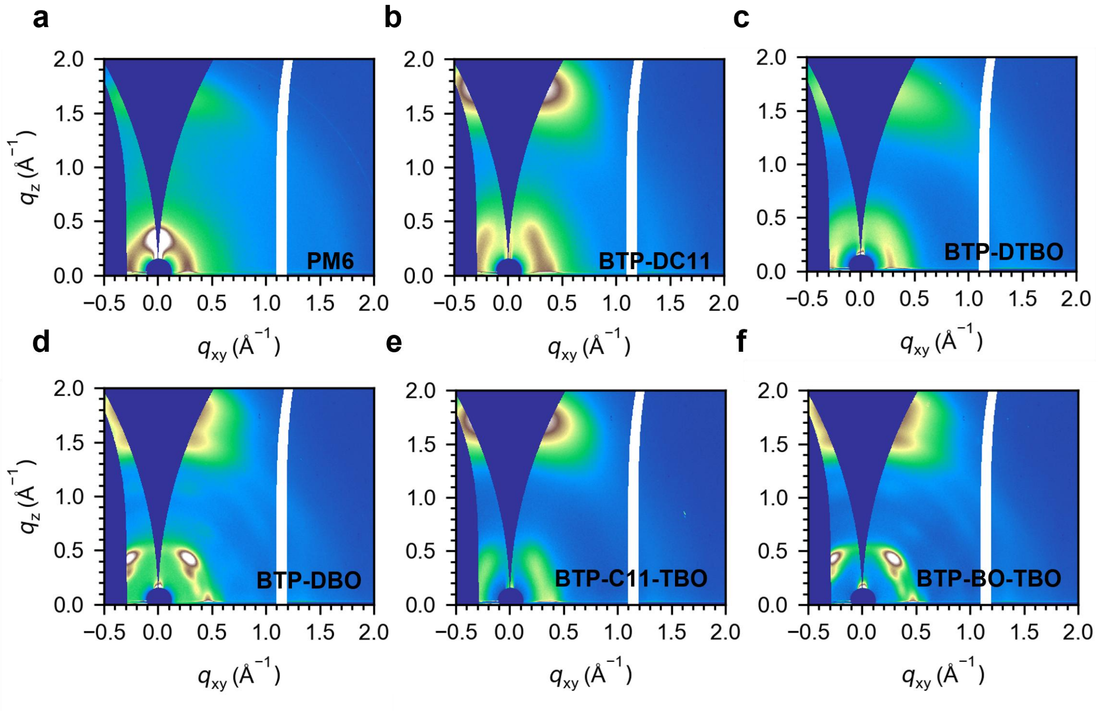

Supplementary Fig. 27 | GIWAXS 2D patterns of neat film. PM6 (**a**), BTP-DC11 (**b**), BTP-DTBO (**c**), BTP-DBO (**d**), BTP-C11-TBO (**e**) and BTP-BO-TBO (**f**).

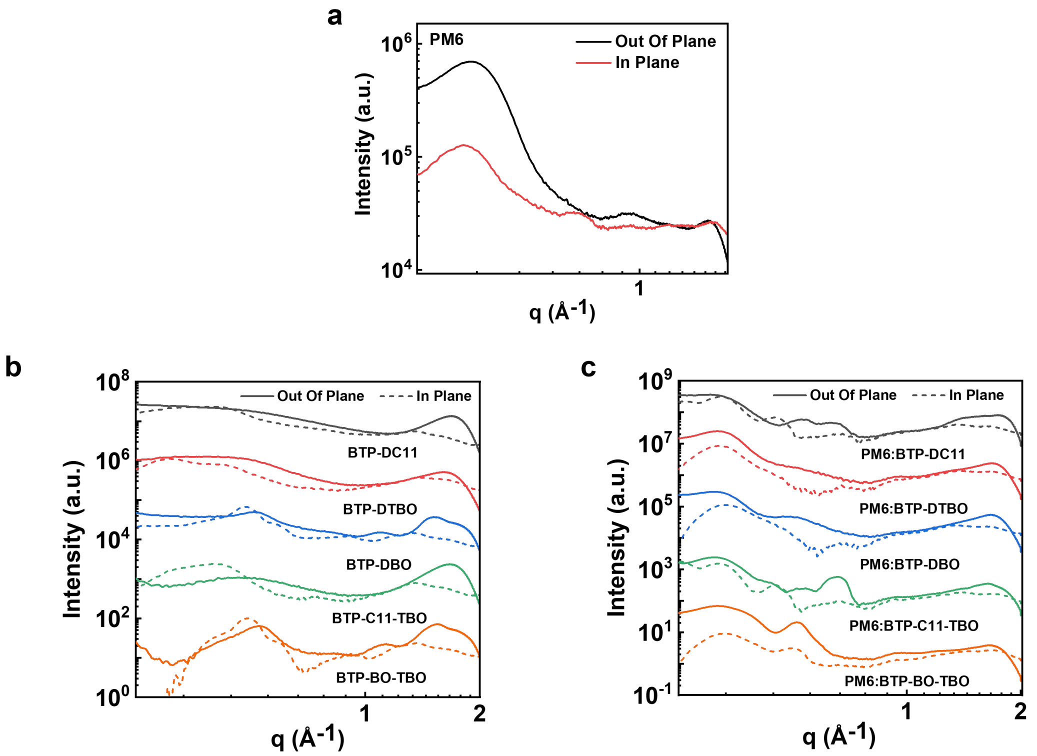

Supplementary Fig. 28 | The GIWAXS 1D line cuts. The neat film of PM6 (**a**) and

BTP-DC11, BTP-DTBO, BTP-DBO, BTP-C11-TBO and BTP-BO-TBO (**b**) and binary blend films of PM6:BTP-DC11; PM6:BTP-DTBO; PM6:BTP-DBO; PM6:BTP-C11-TBO and PM6:BTP-BO-TBO (**c**).

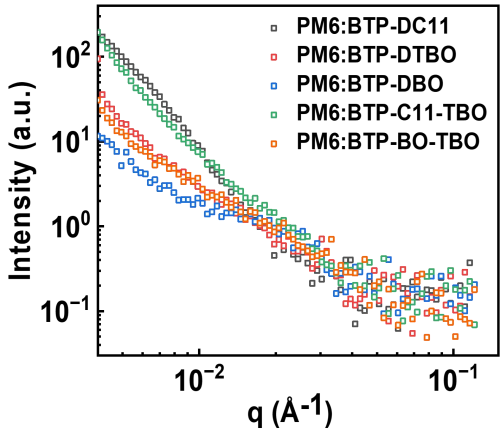

Supplementary Fig. 29 | GISAXS q_r intensity profiles and fitting results. The blends of PM6:BTP-DC11; PM6:BTP-DTBO; PM6:BTP-DBO; PM6:BTP-C11-TBO and PM6:BTP-BO-TBO.

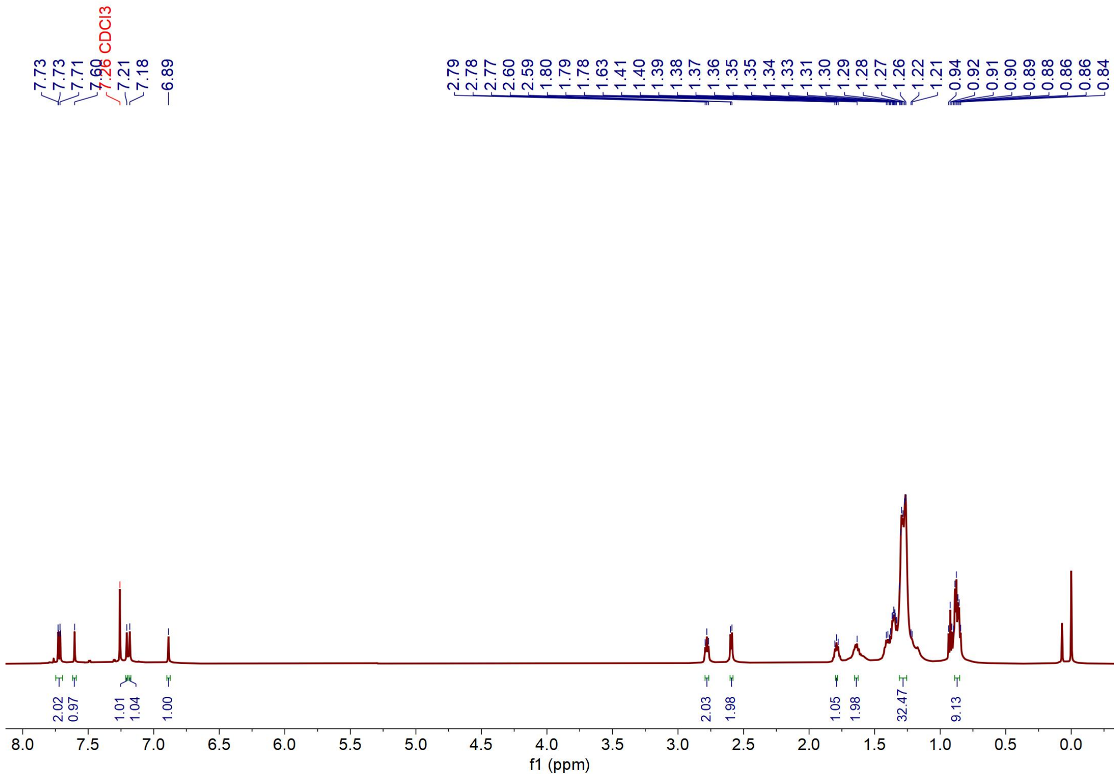

Supplementary Fig. 30 | ^{1}H-NMR spectrum of Compound 3 in $CDCl_3$.

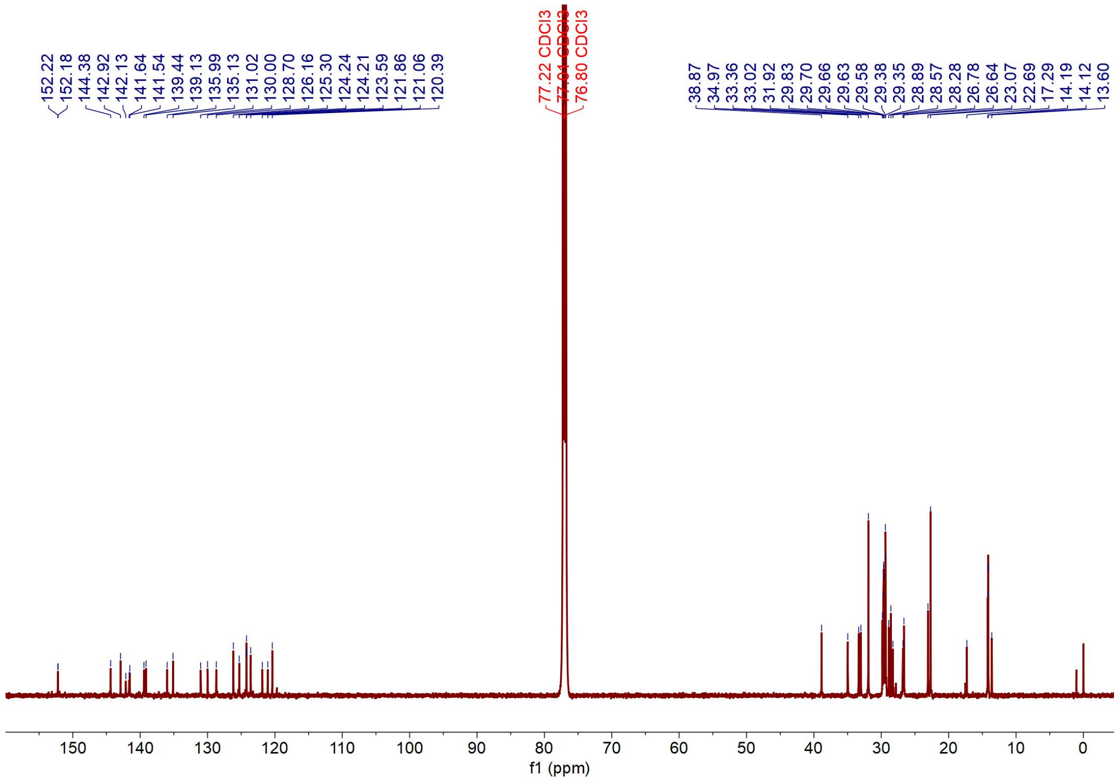

Supplementary Fig. 31 | ^{13}C-NMR spectrum of Compound 3 in $CDCl_3$.

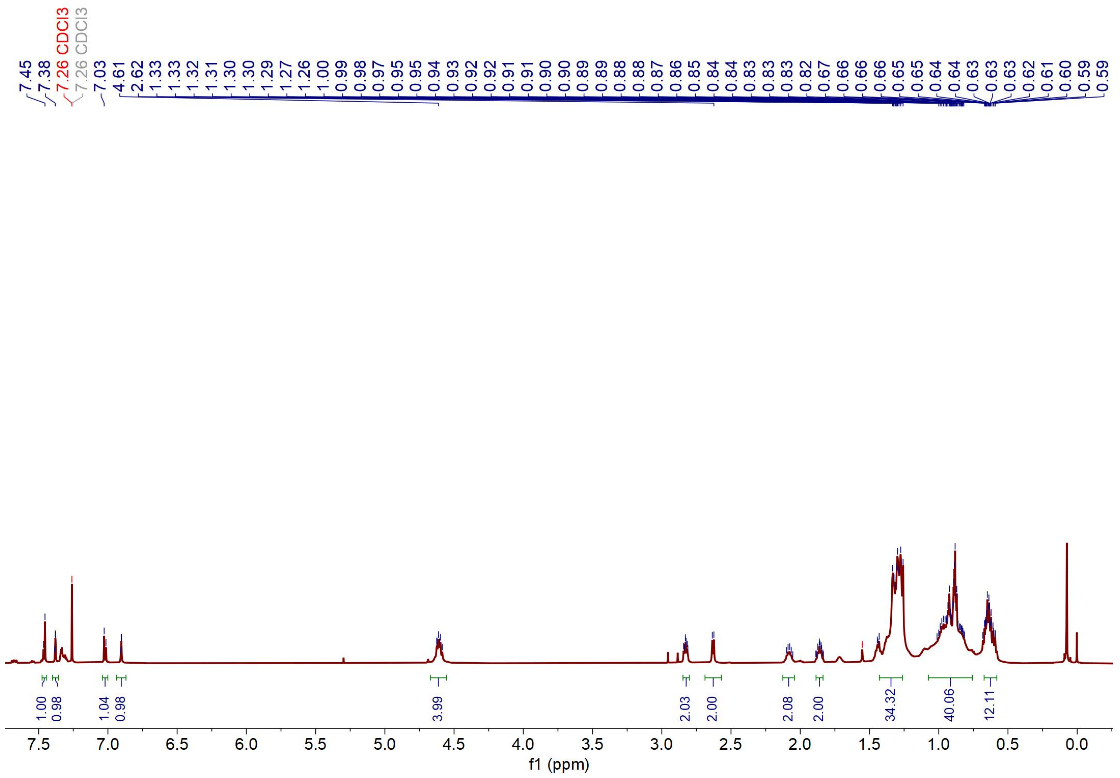

Supplementary Fig. 32 | ^{1}H-NMR spectrum of Compound 4 in $CDCl_3$.

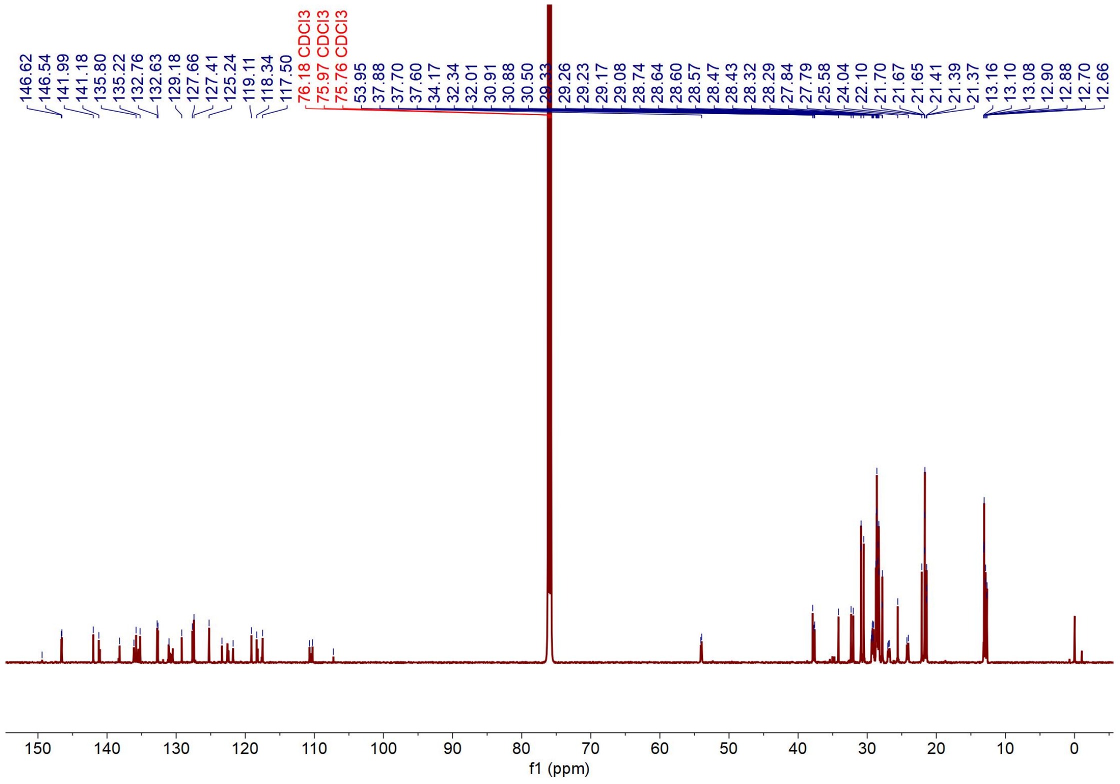

Supplementary Fig. 33 | ^{13}C-NMR spectrum of Compound 4 in $CDCl_3$.

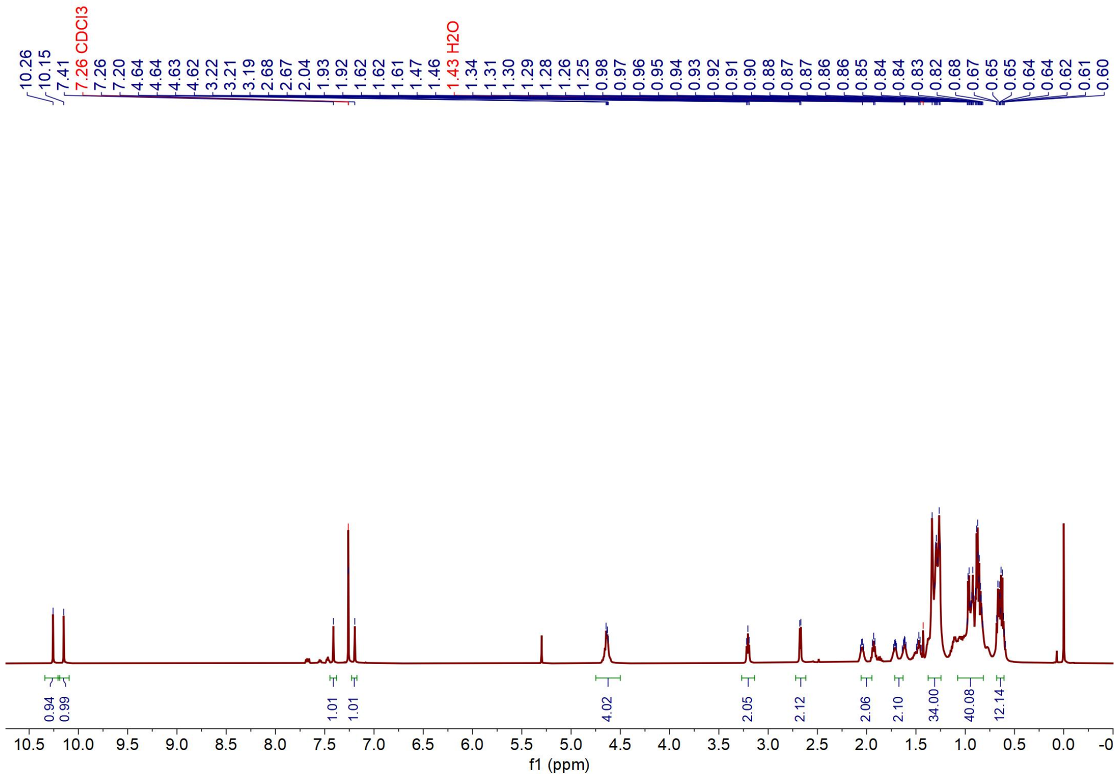

Supplementary Fig. 34 | ^{1}H-NMR spectrum of Compound 5 in $CDCl_3$.

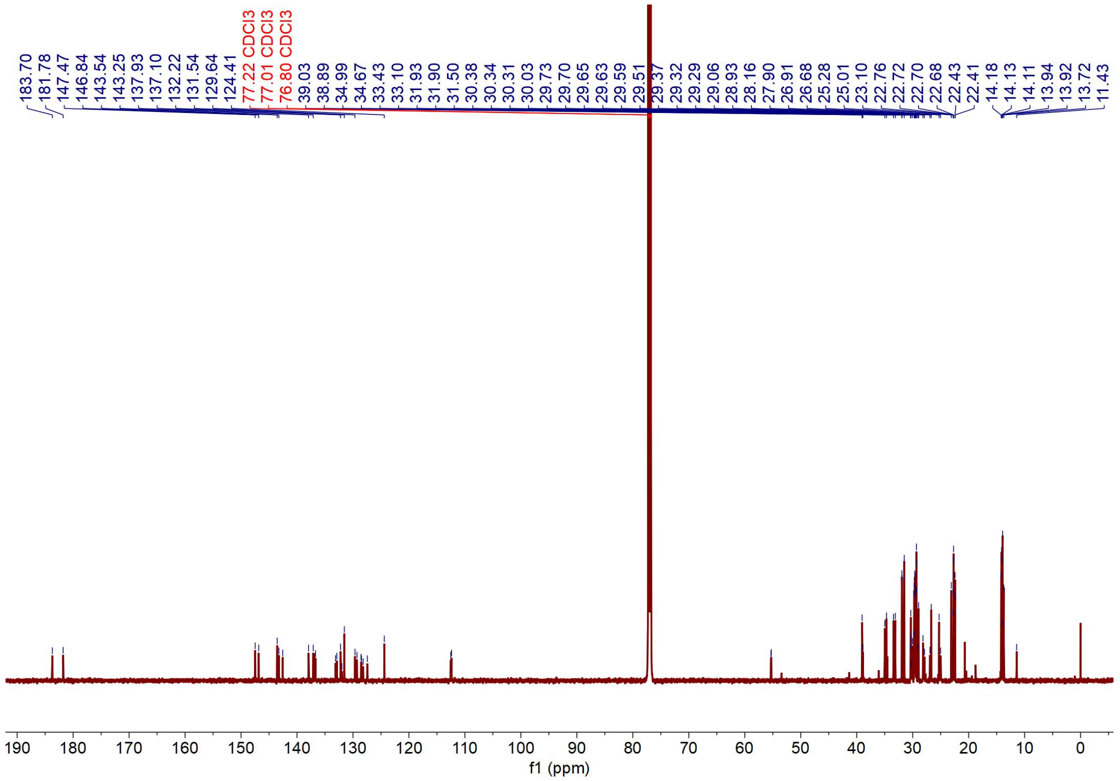

Supplementary Fig. 35 | ^{13}C-NMR spectrum of Compound 5 in $CDCl_3$.

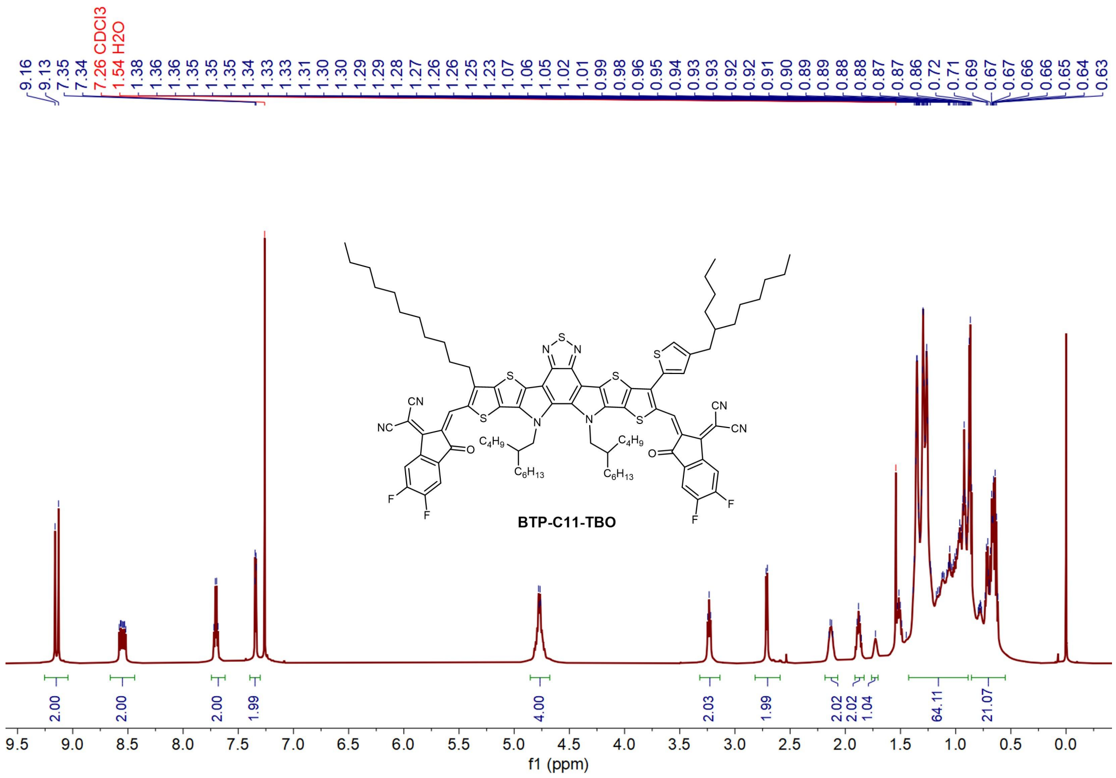

Supplementary Fig. 36 | ^{1}H-NMR spectrum of BTP-C11-TBO in $CDCl_3$.

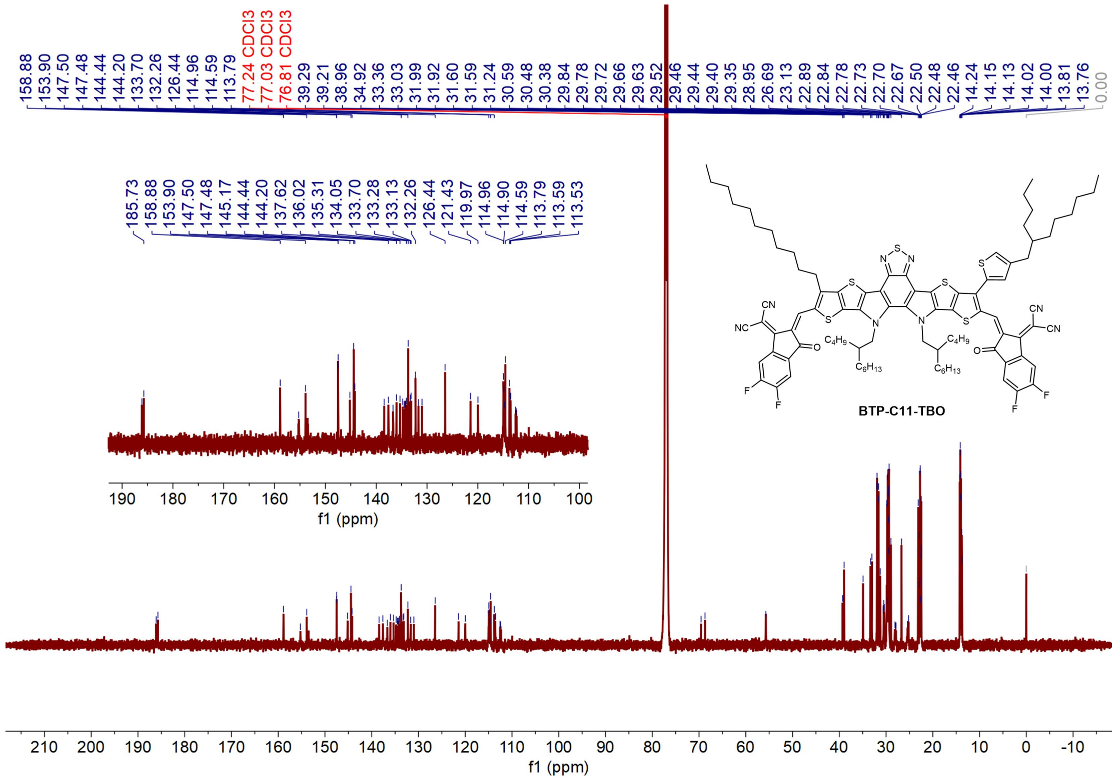

Supplementary Fig. 37 | ^{13}C-NMR spectrum of BTP-C11-TBO in $CDCl_3$.

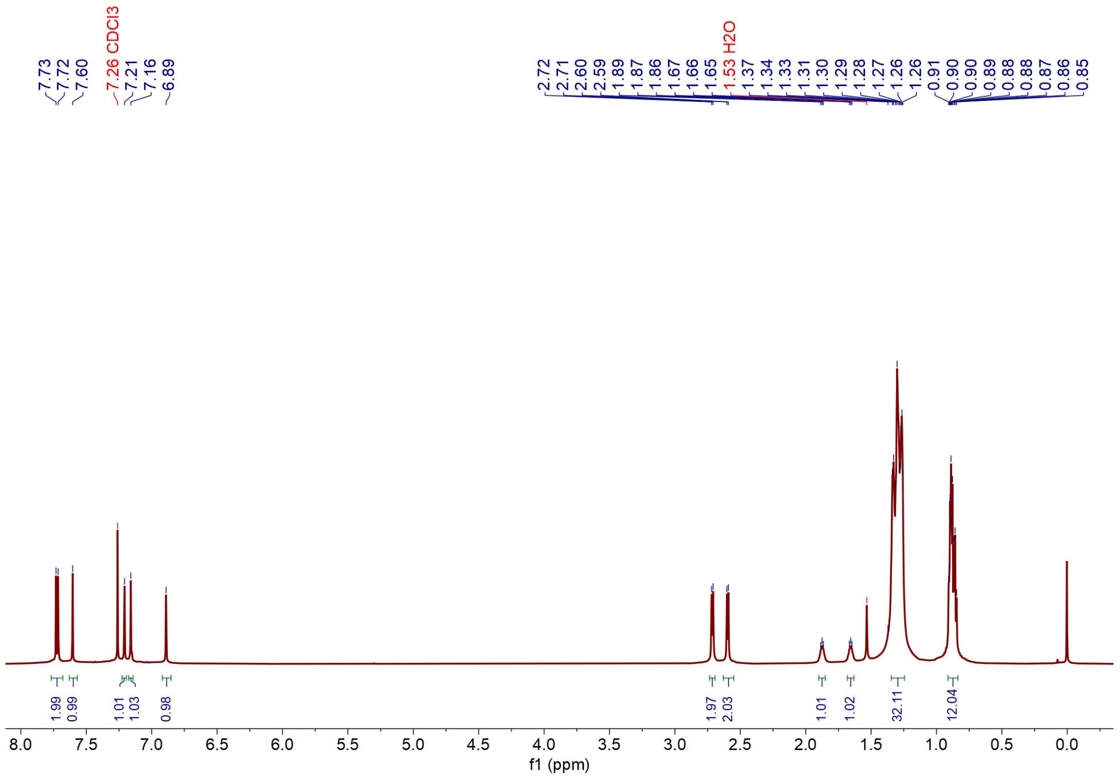

Supplementary Fig. 38 | ^{1}H-NMR spectrum of Compound 3′ in CDCl₃.

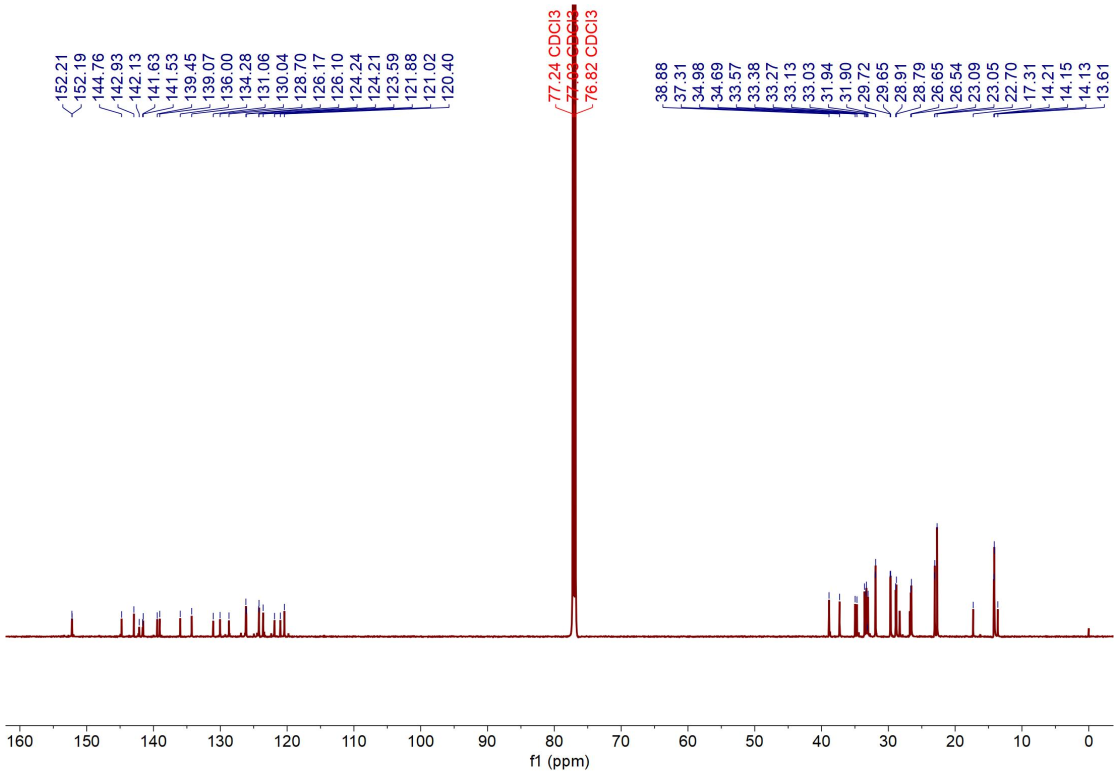

Supplementary Fig. 39 | ^{13}C-NMR spectrum of Compound 3′ in CDCl₃.

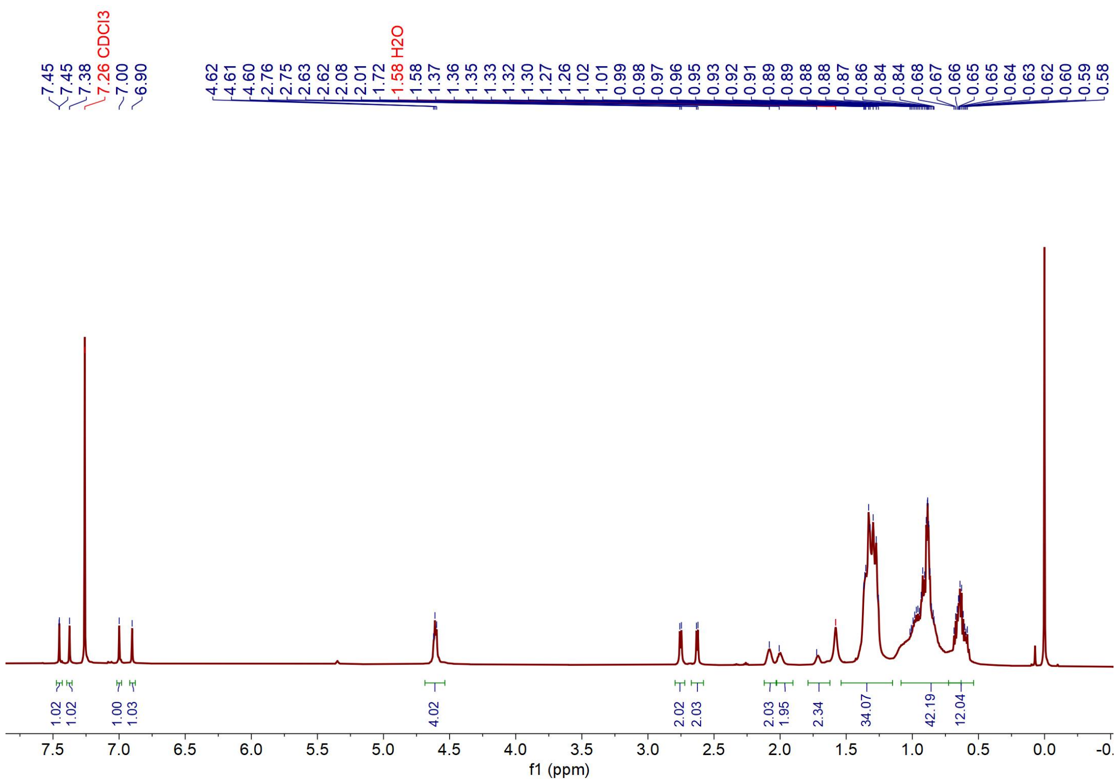

Supplementary Fig. 40 | ^{1}H-NMR spectrum of Compound 4′ in $CDCl_3$.

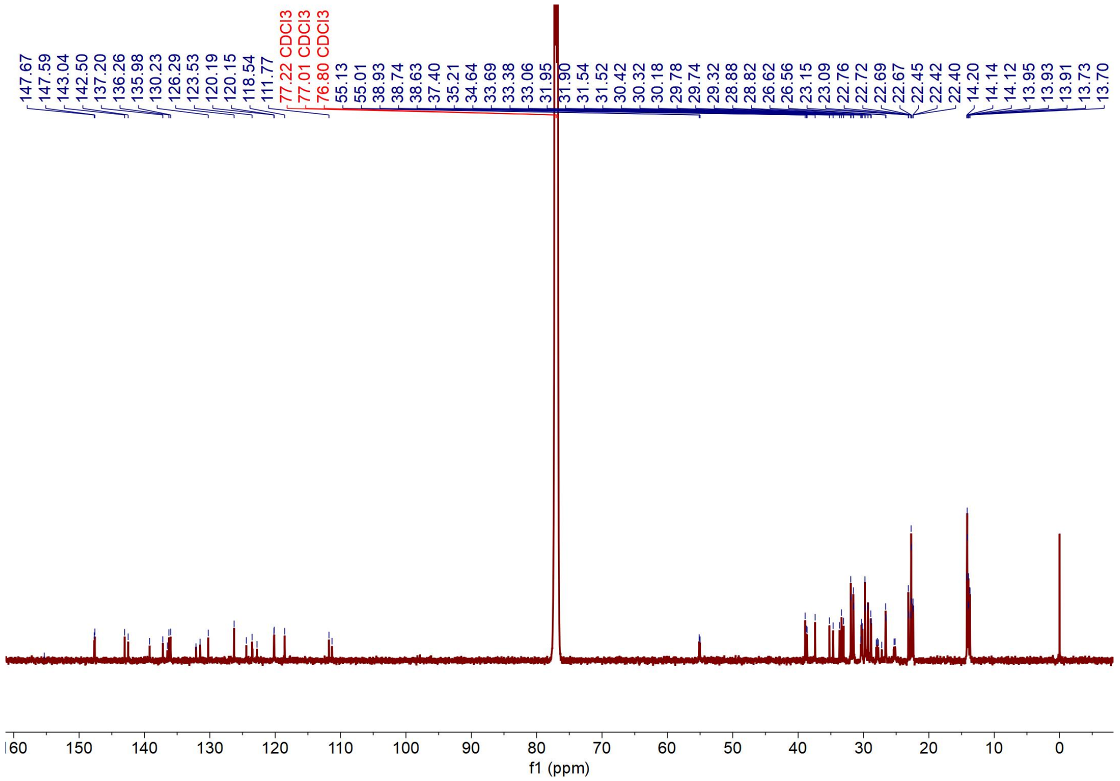

Supplementary Fig. 41 | ^{13}C-NMR spectrum of Compound 4′ in $CDCl_3$.

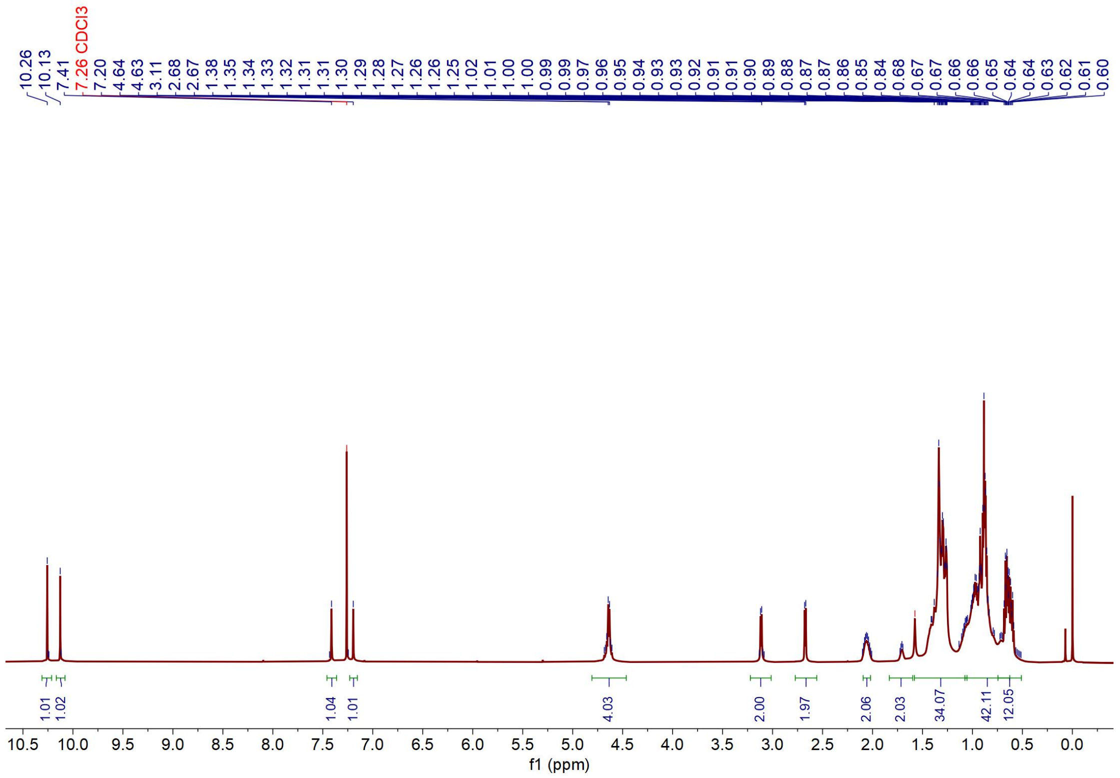

Supplementary Fig. 42 | ^{1}H-NMR spectrum of Compound 5′ in $CDCl_3$.

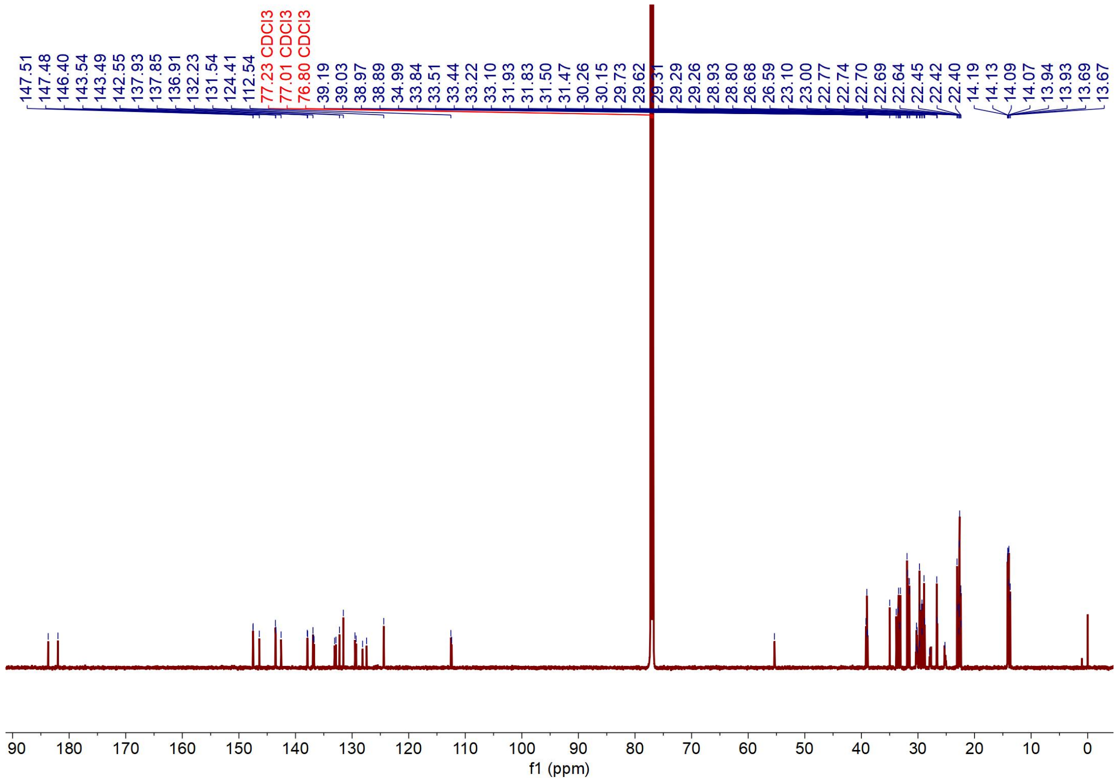

Supplementary Fig. 43 | ^{13}C-NMR spectrum of Compound 5′ in $CDCl_3$.

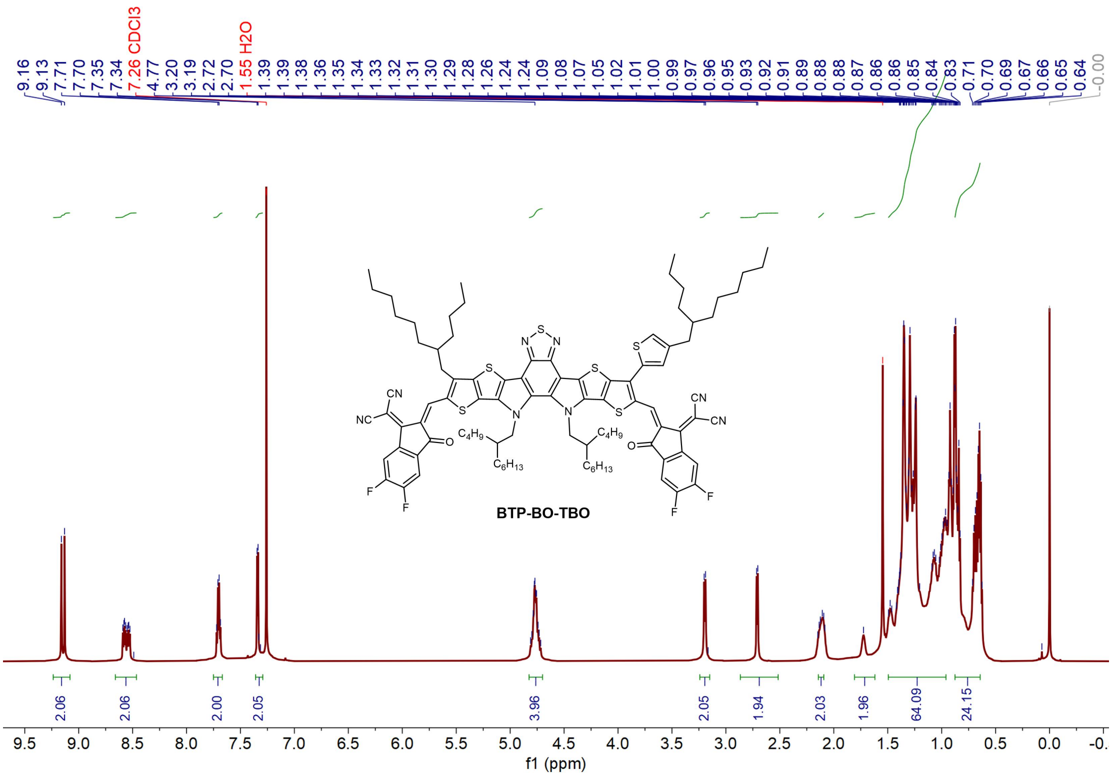

Supplementary Fig. 44 | ^{1}H-NMR spectrum of BTP-BO-TBO in $CDCl_3$.

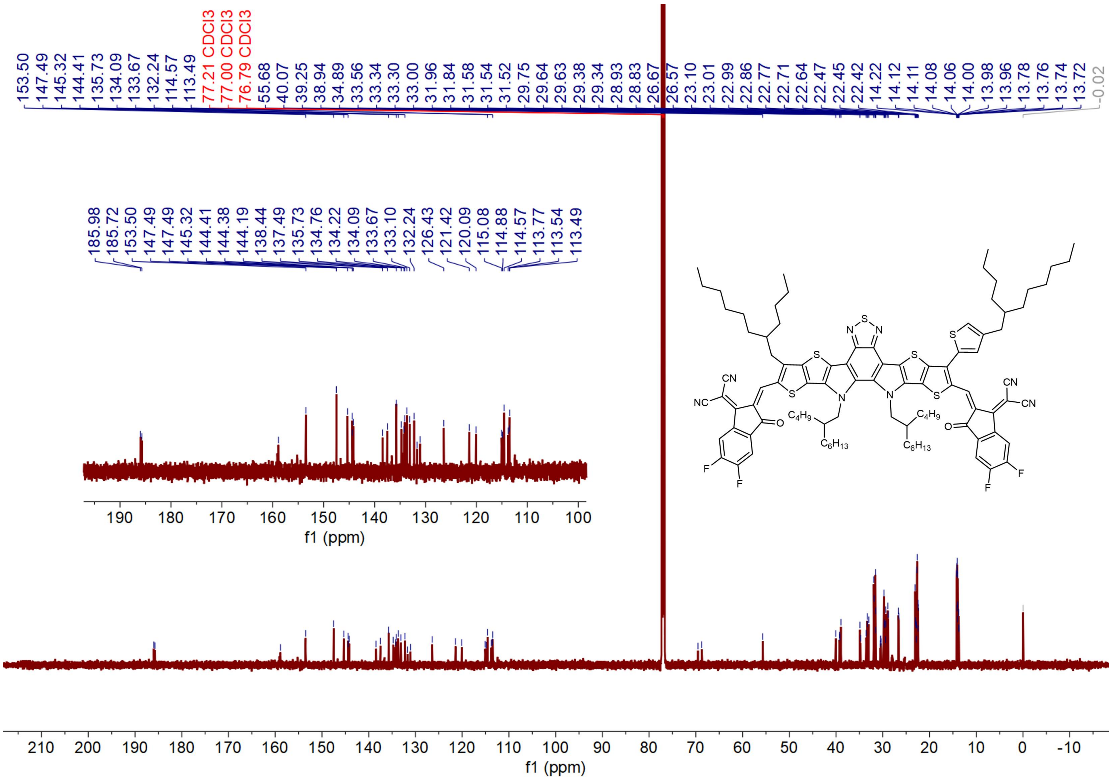

Supplementary Fig. 45 | ^{13}C-NMR spectrum of BTP-BO-TBO in $CDCl_3$.

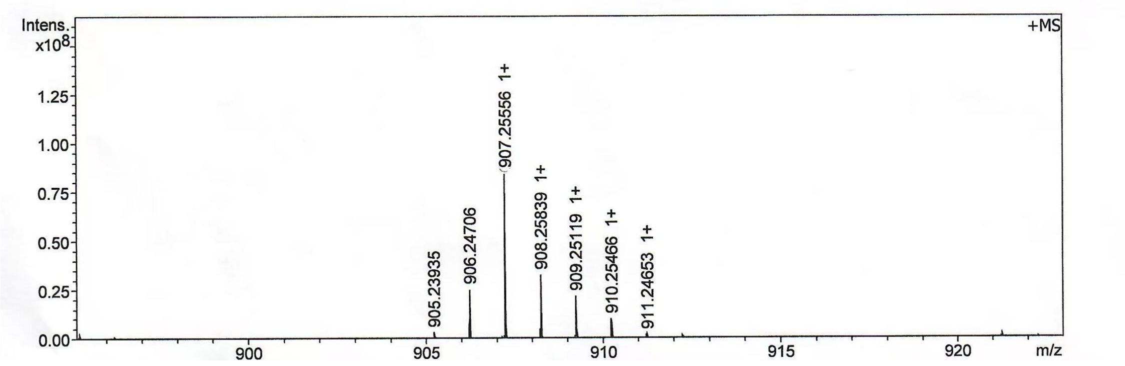

Supplementary Fig. 46 | The mass spectrum (MALDI-TOF) of Compound 3.

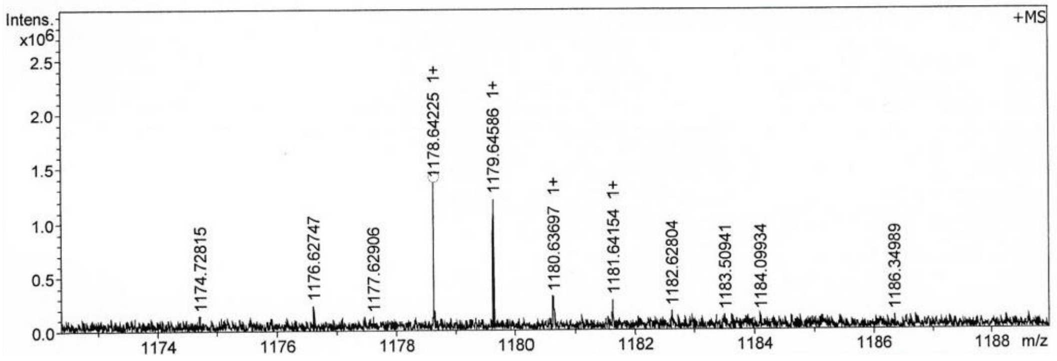

Supplementary Fig. 47 |. The mass spectrum (MALDI-TOF) of Compound 4.

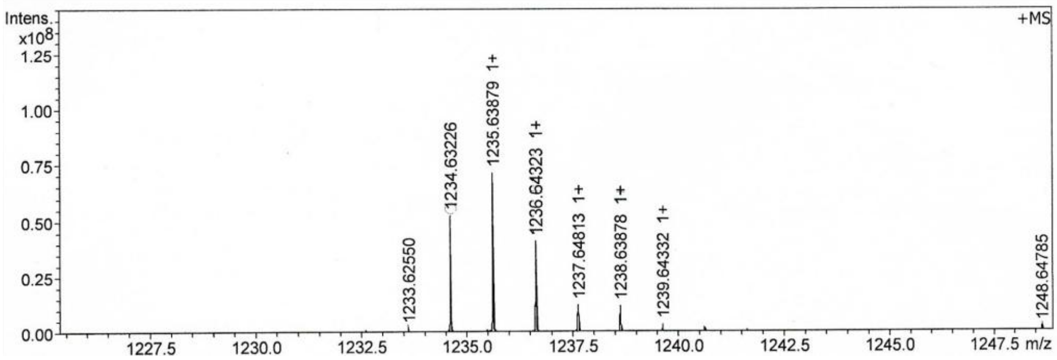

Supplementary Fig. 48 | The mass spectrum (MALDI-TOF) of Compound 5.

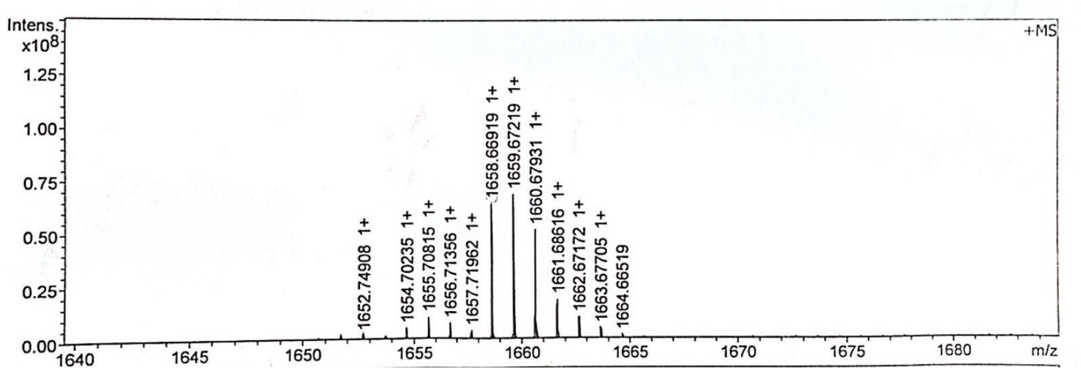

Supplementary Fig. 49 | The mass spectrum (MALDI-TOF) of BTP-C11-TBO.

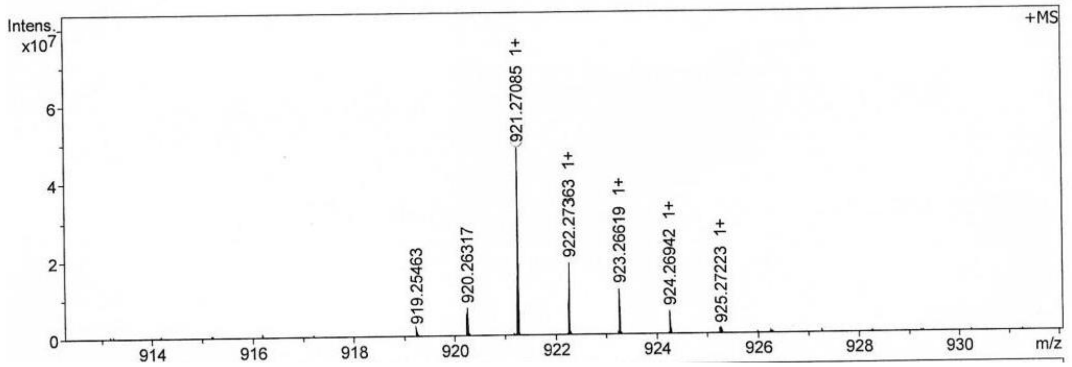

Supplementary Fig. 50 | The mass spectrum (MALDI-TOF) of Compound 3′ .

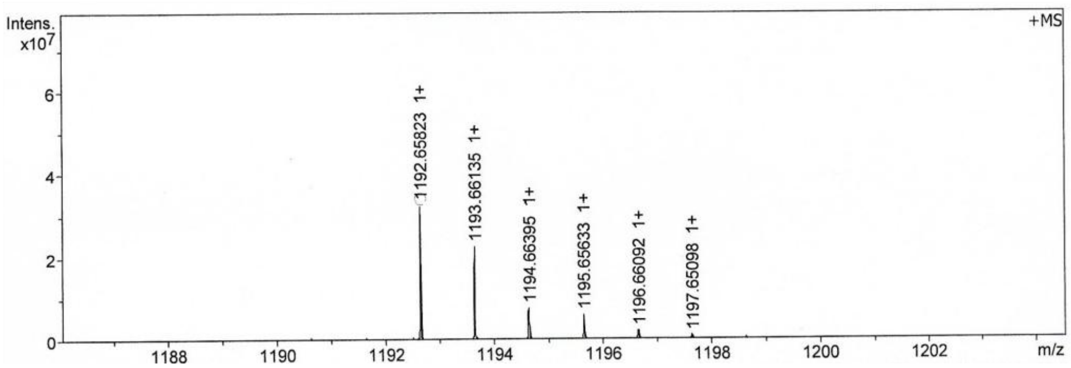

Supplementary Fig. 51 |The mass spectrum (MALDI-TOF) of Compound 4′ .

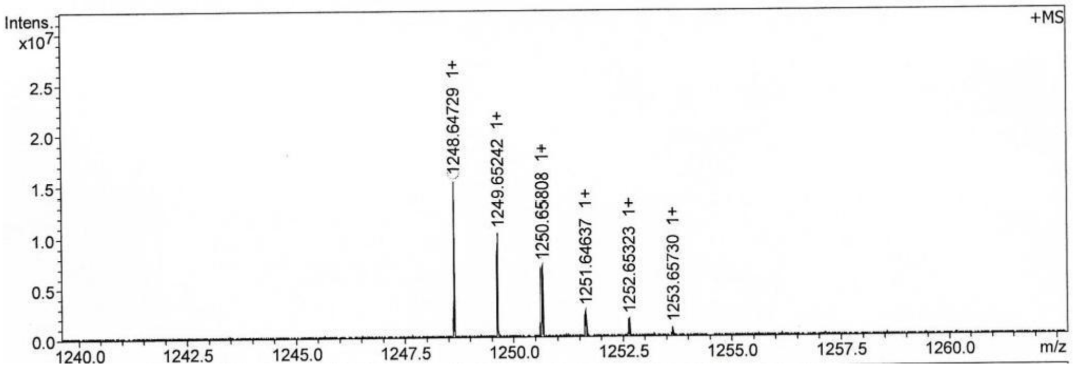

Supplementary Fig. 52 | The mass spectrum (MALDI-TOF) of Compound 5′ .

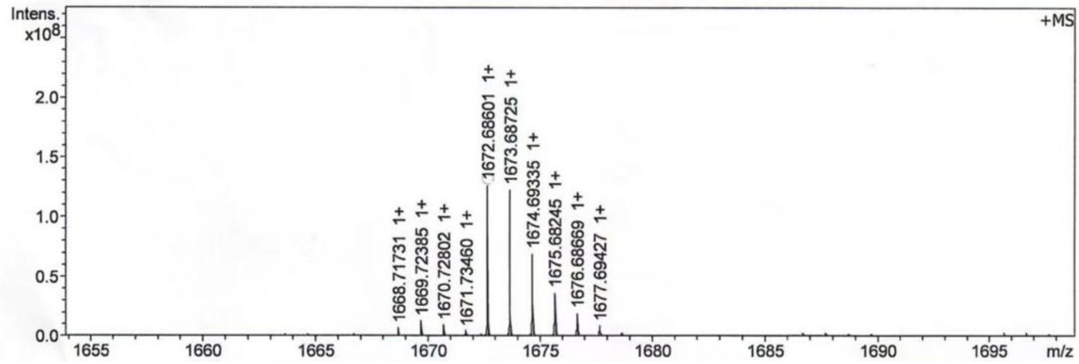

Supplementary Fig. 53 | The mass spectrum (MALDI-TOF) of BTP-C11-TBO.

Supplementary Tables

Supplementary Table 1 | Absorption coefficient of SMAs (BTP-DC11, BTP-DTBO, BTP-DBO, BTP-C11-TBO and BTP-BO-TBO) in dilute chloroform solution and solid state.

Acceptor	Absorption coefficient	
	Solution $\times 10^5$ [M cm^{-1}]	Film $\times 10^5$ [cm^{-1}]
BTP-DC11	2.34	0.97
BTP-DTBO	2.48	0.96
BTP-DBO	2.39	0.97
BTP-C11-TBO	2.54	1.02
BTP-BO-TBO	2.61	1.07

Supplementary Table 2 | PL quantum yield measured of SMAs (BTP-DC11, BTP-DTBO, BTP-DBO, BTP-C11-TBO and BTP-BO-TBO) in solution and film.

Material	Solution	Film
	Φ_{PL} (%)	Φ_{PL} (%)
BTP-DC11	13.49	6.25
BTP-DTBO	8.60	2.62
BTP-DBO	11.04	5.33
BTP-C11-TBO	14.40	6.88
BTP-BO-TBO	15.42	7.02

Supplementary Table 3 | Double-exponential fitting results of photoluminescence decay traces of the original acceptor.

Film	t_1 (ps)	t_2 (ps)
BTP-DC11	52.17 ±1.98	330.22 ±10.8
BTP-DTBO	55.97 ±2.04	300.37 ± 8.52
BTP-DBO	49.09 ±1.38	309.75 ± 8.74
BTP-C11-TBO	53.05 ±2.11	316.60 ± 9.37
BTP-BO-TBO	51.12 ±1.51	316.70 ± 9.23

Supplementary Table 4 | Global fitting of kinetic traces of PM6:SMAs

Active layer	τ_1 (ps)	τ_2 (ps)	τ_3 (ps)
PM6:BTP-DC11	0.51 ±0.04	2.5 ±0.44	1530 ± 82
PM6:BTP-DTBO	0.50 ±0.03	15 ± 0.89	3840 ± 148
PM6:BTP-DBO	0.48 ±0.02	5.2 ± 0.61	3412 ± 124
PM6:BTP-C11-TBO	0.57 ±0.05	3.8 ± 0.26	3515 ± 160
PM6:BTP-BO-TBO	0.49 ±0.02	12 ± 0.47	4178 ± 261

Supplementary Table 5 | The contact angle and the corresponding surface energy of different functional films.

Film	H_2O [°]	CH_2I_2 [°]	SE [mN m^{-1}]	γ_d [mN/m]	γ_P [mN/m]	χ [a.u.]
BDP-DC11	95.3	48.7	35.12	35.09	0.03	0.28K
BDP-BTBO	96.4	43.4	38.34	38.32	0.03	0.64K
BTP-DBO	97.1	45.6	37.14	37.12	0.02	0.49K
BTP-C11-TBO	96.4	43.6	38.22	38.20	0.02	0.62K
BTP-BO-TBO	97.0	42.1	39.19	39.12	0.07	0.74K
PM6	100.3	59.3	29.10	29.08	0.01	\

$$\chi = K(\sqrt{\gamma_D} - \sqrt{\gamma_A})^2$$

Supplementary Table 6 | Photovoltaic parameters of PM6:SMAs at different conditions with 100 °C, 10 min.

Active layer	Ratio	V_{OC} [V]	J_{SC} [mA cm^{-2}]	FF [%]	PCE [%]
PM6:BTP-DC11	1:1	0.854	26.58	78.13	17.73
	1:1.2	0.852	26.50	78.69	17.76
	1:1.5	0.852	26.10	78.39	17.43
PM6:BTP-DTBO	1:1	0.880	25.77	75.25	17.06
	1:1.2	0.881	25.64	76.23	17.22
	1:1.5	0.882	25.59	75.95	17.14
PM6:BTP-DBO	1:1	0.911	25.61	78.33	18.27

	1:1.2	0.909	25.89	78.27	18.42
	1:1.5	0.907	25.75	78.03	18.22
	1:1	0.858	27.32	78.67	18.44
PM6:BTP-C11-TBO	1:1.2	0.856	27.35	79.06	18.51
	1:1.5	0.854	27.25	78.53	18.28
	1:1	0.914	26.63	80.26	19.53
PM6:BTP-BO-TBO	1:1.2	0.913	26.67	81.17	19.76
	1:1.5	0.907	26.42	80.12	19.20

Supplementary Table 7 | Photovoltaic parameters of PM6:SMAs at different conditions.

Active layer	Condition	V_{OC} [V]	J_{SC} [mA cm^{-2}]	FF [%]	PCE [%]
	90 °C	0.854	26.33	78.38	17.62
PM6:BTP-DC11	100 °C	0.852	26.50	78.69	17.76
	110 °C	0.851	26.59	78.27	17.71
	90 °C	0.883	25.45	76.01	17.08
PM6:BTP-DTBO	100 °C	0.881	25.64	76.23	17.22
	110 °C	0.879	25.52	75.81	17.01
	90 °C	0.910	25.67	77.84	18.18
PM6:BTP-DBO	100 °C	0.909	25.89	78.27	18.42
	110 °C	0.905	25.85	77.67	18.17
	90 °C	0.859	27.11	78.50	18.28
PM6:BTP-C11-TBO	100 °C	0.856	27.35	79.06	18.51
	110 °C	0.851	27.37	77.52	18.06

	90 °C	0.915	26.31	80.71	19.43
PM6:BTP-BO-TBO	100 °C	0.913	26.67	81.17	19.76
	110 °C	0.909	26.25	80.24	19.15

Supplementary Table 8 | Charge transport properties of the blend films.

Active layer	Electron mobility (μ_e) ($\times 10^{-4}$cm^2 V^{-1} s^{-1})	Hole mobility (μ_h) ($\times 10^{-4}$cm^2 V^{-1} s^{-1})	μ_e/μ_h
PM6:BTP-DC11	1.36	1.95	0.70
PM6:BTP-DTBO	1.32	1.72	0.77
PM6:BTP-DBO	1.92	2.34	0.83
PM6:BTP-C11-TBO	2.57	2.88	0.89
PM6:BTP-BO-TBO	3.47	3.73	0.93

Supplementary Table 9 | Multi-fitting data statistics of s-EQE and EL curves for calculating $\Delta E_{LE\text{-}CT}$.

Active layer	E_{LE} [eV]	E_{CT} [eV]	$\Delta E_{LE\text{-}CT}$ [eV]
PM6:BTP-DC11	1.37	1.34 (1.346±0.0115) [a]	0.03 (0.0233±0.0115) [a]
PM6:BTP-DTBO	1.43	1.38 (1.3775±0.005)	0.05 (0.0525±0.005)
PM6:BTP-DBO	1.45	1.42 (1.426±0.0115)	0.03 (0.0233±0.0115)
PM6:BTP-C11-TBO	1.39	1.36 (1.363±0.0057)	0.03 (0.0267±0.0057)
PM6:BTP-BO-TBO	1.40	1.38 (1.385±0.0058)	0.02 (0.0150±0.0058)

[a)] The values in parentheses are the average values with standard deviations obtained from over 3 fits.

Supplementary Table 10 | Crystal data for BTP-DBO (CCDC 2405808), BTP-DTBO (CCDC 2405811) and BTP-BO-TBO (CCDC 2405812).

Acceptor	BTP-DBO	BTP-DTBO	BTP-BO-TBO
Space group	C2/c	C2/c	C2/c
a [Å]	27.0612(7)	27.5504(13)	28.0817(11)
b [Å]	22.3128(6)	57.0641(14)	56.7025(17)
c [Å]	32.2933(9)	13.5264(5)	13.5233(5)
alpha [°]	90	90	90
beta [°]	110.561(3)	93.431(4)	93.635(4)
gamma [°]	90	90	90
Volume [Å^3]	18260.3(9)	21227.3(14)	21489.9(13)

Supplementary Table 11 | Electron coupling of BTP-DBO, BTP-DTBO, and BTP-BO-TBO. Dimers refer to the numbering scheme in the figures in the main text.

Dimer	|J|/meV		
	BTP-DBO	BTP-DTBO	BTP-BO-TBO
1-2	10.98	15.76	36.09
1-3	8.30	10.06	11.05
1-4	26.06	63.79	5.07
1-5	8.30	15.76	7.05

Supplementary Table 12 | Structure parameters of various neat and binary films obtained from GIWAXS 2D patterns.

Thin films	q_r-peak position (Å^{-1})	*d*-spacing (Å)	CCL (Å)	FWHM (Å^{-1})	q_z-peak position (Å^{-1})	d-spacing (Å)	CCL (Å)	FWHM (Å^{-1})
PM6	0.322	19.51	40.99	0.1338	1.639	3.834	32.28	0.177
BTP-DC11	0.391	16.07	36.24	0.158	1.692	3.713	12.68	0.451
BTP-DTBO	0.352	17.85	27.59	0.205	1.615	3.891	15.56	0.367
BTP-DBO	0.485	12.95	74.45	0.076	1.767	3.556	29.67	0.193
BTP-C11-TBO	0.398	15.78	37.95	0.149	1.672	3.758	14.74	0.389
BTP-BO-TBO	0.492	12.77	71.65	0.079	1.778	3.534	28.21	0.203

Supplementary Table 13 | Structure parameters of various blend films obtained from GIWAXS 2D patterns.

PM6:SMAs	q_r-peak position(Å^{-1})	*d*-spacing (Å)	FWHM (Å^{-1})	CCL (Å)	q_z-peak position (Å^{-1})	*d*-spacing (Å)	FWHM (Å^{-1})	CCL (Å)
BTP-DC11	0.326	19.27	0.050	113.1	1.679	3.742	0.367	15.578
BTP-DTBO	0.323	19.24	0.086	65.78	1.660	3.785	0.343	16.66
BTP-DBO	0.333	18.87	0.090	62.86	1.663	3.778	0.308	18.56
BTP-C11-TBO	0.328	19.16	0.046	122.9	1.645	3.820	0.316	18.08
BTP-BO-TBO	0.333	18.87	0.084	67.35	1.671	3.760	0.303	18.87

Supplementary Table 14 | GISAXS q_r intensity profiles fitting results based on PM6:SMAs blend films.

Active layer	$2R_g$ (nm)
PM6:BTP-DC11	50
PM6:BTP-DTBO	27
PM6:BTP-DBO	20
PM6:BTP-C11-TBO	17
PM6:BTP-BO-TBO	26

Supplementary Methods

Synthetic Procedures of BTP-C11-TBO and BTP-BO-TBO were shown in Supplementary Figs. 1. Raw materials such as compounds **Tributyl(4-(2-butyloc tyl)thiophen-2-yl)stannane, (6-bromothieno[3,2-b]thiophen-2-yl)tributylstannan-e, 3-bromothieno[3,2-b]thiophene, 4,7-dibromo-5,6-dinitrobenzo[c][1,2,5]thiadi-azole, Tributyl(6-undecylthieno[3,2-b]thiophen-2-yl)stannane,Tributyl(6-(2-buty l-octyl)thieno[3,2-b]thiophen-2-yl)stannane, 5-(bromomethyl)undecane** and **2-(5, 6-difluoro-3-oxo-2,3,3a,7a-tetrahydro-1H-inden-1-ylidene)malononitrile (IC-2F)** were commercially available from Solarmer Materials Inc. $Pd(PPh_3)_4$ was obtai ned from J&K Chemical Co. Toluene was dried over Na/benzophenone and fre shly distilled before use. The other chemicals, solvents and materials used in t his work were all commercially available and used without further purification.

Synthesis of Compound 1

A mixture of compound tributyl(4-(2-butyloctyl) thiophen-2-yl) stannane (5.43 g, 10 mmol), (6-bromothieno[3,2-b]thiophen-2-yl)tributylstannane (2.19 g, 10 mmol), and $Pd(PPh_3)_4$ (0.35 g, 0.30 mmol) were dissolved in anhydrous toluene (40.0 mL) and stirred at 110 °C overnight under argon atmosphere. After being cooled to room temperature, the solvent was then removed under reduced pressure. The residue was washed with water and extracted with hexane. Without any further purification, the product of Compound **1** was used in the following reaction.

Synthesis of Compound 2

To a solution of compound **1** in anhydrous tetrahydrofuran (40 mL), 2.0 M

lithium diisopropylamide (4.0 mL, 8.0 mmol) was added dropwise at -78 °C under an argon atmosphere. After stirring at the same temperature for 3.5 h, 1.0 M trimethyl tin chloride (8.0 mL, 8.0 mmol) was added dropwise at -78 °C, and the mixture was gradually warmed to room temperature. After stirring overnight, the mixture was quenched with saturated KF aqueous solution and extracted with hexane. The organic layer was dried with Na_2SO_4. Removing the solvent under reduced pressure gave the crude compound **2**. Without any further purification, the product was used in the following reaction.

Synthesis of Compound 3 and 3'

Compound **2** (2.22 g, 4.0 mmol) , 4,7-dibromo-5,6-dinitrobenzo[c][1,2,5]thi-adiazole (1.53 g, 4.0 mmol), Tributyl(6-undecylthieno[3,2-b]thiophen-2-yl)stannan-e (2.33 g, 4 mmol)/Tributyl(6-(2-butyloctyl)thieno[3,2-b]thiophen-2-yl)stannane (2.39 g, 4 mmol) and $Pd(PPh_3)_4$ (0.60 g, 0.51 mmol) were dissolved in anhydr ous toluene (30.0 mL) and stirred at 110 °C overnight under argon atmosphere. Then, the reaction mixture was cooled and poured into a saturated KF aqueou s solution. The mixture was extracted with diethyl ether three times. The comb ined organic phase was washed with water followed by brine. Then, the soluti on was dried over Na_2SO_4 and concentrated under reduced pressure. Compound **3** was purified by chromatography in a silica gel column eluting with petrole um ether/dichloromethane (4/1, v/v), which yields a red powder as the product of Compound **3** (2.63 g, 72.6% yield) and **3'** (2.52 g, 68.3% yield).

Compound 3： ^{1}H NMR (600 MHz, $CDCl_3$) δ 7.72 (dd, *J*=8.9, 1.4 Hz, 2H), 7.60 (s, 1H), 7.21 (s, 1H), 7.18 (s, 1H), 6.89 (s, 1H), 2.78 (t, *J*=7.7 Hz, 2H), 2.59 (d, *J*=6.8 Hz, 2H), 1.79 (s, 1H), 1.63 (s, 2H), 1.31-1.26 (m, 32H), 0.87 (dd, *J* =13.1, 6.0 Hz, 9H). ^{13}C NMR (151 MHz, CDCl3) δ 152.22, 152.18, 144.38, 142.92, 142.13, 141.64, 141.54, 139.44, 139.13, 135.99, 135.13, 131.02, 130.00, 128.70, 126.16, 125.30, 124.24, 124.21, 123.59, 121.86, 121.06, 120.39, 38.87, 34.97, 33.36, 33.02, 31.92, 29.83, 29.70, 29.66, 29.63, 29.58, 29.38, 29.35, 28.89, 28.57, 28.28, 26.78, 26.64, 23.07, 22.69, 17.29, 14.19, 14.12, 13.60. HRMS (MALDI-TOF) (m/z): [M]+ calculated for $C_{45}H_{54}N_4O_4S_6$, 907.25; found: 907.25.

Compound 3'： ^{1}H NMR (600 MHz, $CDCl_3$) δ 7.72 (d, *J*=9.6 Hz, 2H), 7.60 (s, 1H), 7.21 (s, 1H), 7.16 (s, 1H), 6.89 (s, 1H), 2.71 (d, *J*=7.0 Hz, 2H), 2.60 (d, *J*=6.8 Hz, 2H), 1.87 (t, *J*=6.2 Hz, 1H), 1.68-1.63 (m, 1H), 1.35-1.24 (m, 32H), 0.91-0.84 (m, 12H). ^{13}C NMR (151 MHz, CDCl3) δ 152.21, 152.19, 144.76, 142.93, 142.13, 141.63, 141.53, 139.45, 139.07, 136.00, 134.28, 131.06, 130.04, 128.70, 126.17, 126.10, 124.24, 124.21, 123.59, 121.88, 121.02, 120.40, 38.88, 37.31, 34.98, 34.69, 33.57, 33.38, 33.27, 33.13, 33.03, 31.94, 31.90, 29.72, 29.65, 28.91, 28.79, 26.65, 26.54, 23.09, 23.05, 22.70, 17.31, 14.21, 14.15, 14.13, 13.61. HRMS (MALDI-TOF) (m/z): $[M]^+$ calculated for $C_{46}H_{56}N_4O_4S_6$, 921.26; found: 921.27.

Synthesis of Compound 4 and 4'

Compound **3** (2.27 g, 2.50 mmol) / **3'** (2.28 g, 2.50 mmol) and triethyl phosphate (10 mL) were dissolved in anhydrous 1,2-dichlorobenzene (o-DCB, 10 mL) under argon and the mixture was stirred at 180 °C overnight. After cooling to room

temperature, the solvent was removed by vacuum distillation. Subsequently, the red intermediate was then mixed with K_2CO_3 (3.45 g, 25.0 mmol), KI (0.42 g, 2.50 mmol), 5-(bromomethyl) decane (1.87 g, 7.50 mmol), and anhydrous DMF (25 mL) were mixed under argon and stirred at 100 °C overnight. The mixture was extracted with ethyl acetate ether three times. The combined organic phase was washed with water followed by brine. Then, the solution was dried over Na_2SO_4 and concentrated under reduced pressure, by column chromatography on silica gel using petroleum ether/dichloromethane (5/1, v/v) as the eluent to give an orange solid Compound **4** (1.58 g, 53.5 % yield) and Compound **4'** (1.85 g, 62.2 % yield).

Compound 4： ^{1}H NMR (600 MHz, $CDCl_3$) δ 7.46 (d, *J*=7.1 Hz, 1H), 7.38 (d, *J*=1.4 Hz, 1H), 7.02 (d, *J*=7.8 Hz, 1H), 6.90 (d, *J*=1.3 Hz, 1H), 4.67-4.55 (m, 4H), 2.83 (td, *J* =7.8, 3.0 Hz, 2H), 2.63 (d, *J*=6.9 Hz, 2H), 2.08 (p, *J*=6.6 Hz, 2H), 1.89-1.84 (m, 2H), 1.43-1.26 (m, 34H), 1.07-0.76 (m, 40H), 0.67-0.58 (m, 12H). ^{13}C NMR (151 MHz, $CDCl_3$) δ 149.40, 146.62, 146.54, 141.99, 141.18, 138.18, 136.11, 135.80, 135.22, 132.76, 132.63, 131.03, 129.18, 127.66, 127.41, 125.24, 123.37, 121.76, 119.11, 118.34, 117.50, 110.71, 110.28, 107.25, 54.08, 53.95, 37.88, 37.70, 37.60, 34.17, 32.34, 32.01, 30.91, 30.88, 30.50, 29.38, 29.33, 29.26, 29.23, 29.17, 29.08, 28.74, 28.64, 28.60, 28.57, 28.47, 28.43, 28.32, 28.29, 27.84, 27.79, 27.02, 26.93, 26.76, 25.58, 24.25, 24.04, 22.10, 21.70, 21.67, 21.65, 21.41, 21.39, 21.37, 13.16, 13.10, 13.08, 12.90, 12.88, 12.70, 12.66. HRMS (MALDI-TOF) (m/z): $[M]^+$ calculated for $C_{69}H_{102}N_4O_2S_6$, 1178.64; found: 1178.64.

Compound 4'：^{1}H NMR (600 MHz, $CDCl_3$) δ 7.45 (d, *J*=1.1 Hz, 1H), 7.38 (s, 1H), 7.00 (s, 1H), 6.90 (s, 1H), 4.61 (t, *J*=7.0 Hz, 4H), 2.75 (d, *J*=7.2 Hz, 2H), 2.63 (d, *J*= 6.9 Hz, 2H), 2.08 (s, 2H), 2.01 (s, 2H), 1.72 (s, 2H), 1.54-1.15 (m, 34H), 1.09-0.63 (m, 42H), 0.73-0.54 (m, 12H). ^{13}C NMR (151 MHz, $CDCl_3$) δ 147.67, 147.59, 143.04, 142.50, 139.22, 137.20, 136.51, 136.26, 135.98, 132.12, 131.49, 130.23, 126.29, 124.42, 123.53, 122.78, 120.19, 120.15, 118.54, 111.77, 111.30, 55.13, 55.01, 38.93, 38.74, 38.63, 37.40, 35.21, 34.64, 33.69, 33.38, 33.06, 31.95, 31.90, 31.54, 31.52, 30.42, 30.32, 30.18, 29.78, 29.74, 29.32, 28.88, 28.82, 28.04, 27.89, 27.73, 27.21, 26.62, 26.56, 25.32, 25.17, 23.15, 23.09, 22.76, 22.72, 22.69, 22.67, 22.45, 22.42, 22.40, 14.20, 14.14, 14.12, 13.95, 13.93, 13.91, 13.73, 13.70. HRMS (MALDI-TOF) (m/z): [M]+ calculated for $C_{70}H_{104}N_4O_2S_6$, 1192.66; found: 1192.66.

Synthesis of Compound 5 and 5'

Compound **4** (1.18 g, 1.0 mmol) /**4'** (1.19 g, 1.0 mmol) was dissolved in 20 mL trichloromethane under argon, then the fresh Valmeyer reagent (3.6 mL $POCl_3$ in 7.7 mL DMF) was added dropwise at 0 °C. After stirring for 20 min at 0 °C, the mixture was heated to 65 °C and reacted for 24 h. The reaction was quenched with saturated $NaHCO_3$ solution and allowed to stir at room temperature for 24 h. The organic layer was separated, and the aqueous phase was extracted with diethyl ether three times. The combined organic layer was washed with brine, dried over Na_2SO_4, filtered, and concentrated under reduced pressure. The crude product was purified with a column chromatograph on silica gel using petroleum ether/dichloromethane (2/1, v/v) as the eluent to give an orange solid of Compound **5** (1.05 g, 85.3 % yield) and 5' (1.09 g,

87.4 % yield).

Compound 5：^{1}H NMR (600 MHz, $CDCl_3$) δ 10.26 (s, 1H), 10.15 (s, 1H), 7.41 (s, 1H), 7.20 (s, 1H), 4.63 (dd, *J*=8.4, 5.1 Hz, 4H), 3.21 (t, *J*=7.8 Hz, 2H), 2.67 (d, *J*=6.8 Hz, 2H), 2.06-1.95 (m, 2H), 1.71-1.63 (m, 2H), 1.38-1.25 (m, 34H), 1.08-0.82 (m, 40H), 0.68-0.61 (m, 12H). ^{13}C NMR (151 MHz, CDCl3) δ 155.77, 147.64, 142.07, 137.03, 137.01, 136.83, 132.75, 132.16, 132.10, 132.06, 131.99, 131.97, 131.70, 131.68, 130.30, 128.56, 128.48, 123.65, 122.79, 122.75, 119.23, 111.48, 70.79, 54.99, 38.63, 37.44, 34.67, 34.53, 31.93, 31.82, 31.59, 31.55, 30.99, 30.67, 30.39, 30.25, 30.11, 29.71, 29.69, 29.64, 29.61, 29.51, 29.47, 29.36, 29.34, 29.31, 29.06, 28.87, 28.84, 28.01, 27.80, 27.67, 26.92, 26.63, 25.28, 22.97, 22.75, 22.70, 22.65, 22.62, 22.44, 22.42, 14.09, 14.05, 13.95, 13.93, 13.74, 13.69, 11.44. HRMS (MALDI-TOF) (m/z): $[M]^+$ calculated for $C_{71}H_{102}N_4O_2S_6$, 1234.63; found: 1234.63.

Compound 5'：^{1}H NMR (600 MHz, $CDCl_3$) δ 10.26 (s, 1H), 10.13 (s, 1H), 7.41 (s, 1H), 7.20 (s, 1H), 4.64 (q, *J*=7.5 Hz, 4H), 3.11 (d, *J*=7.5 Hz, 2H), 2.67 (d, *J*=6.8 Hz, 2H), 2.06 (tt, *J*=12.9, 6.3 Hz, 2H), 1.71 (q, *J*=5.9 Hz, 2H), 1.58-1.05 (m, 34H), 1.08-0.63 (m, 42H), 0.74-0.51 (m, 12H). ^{13}C NMR (151 MHz, CDCl3) δ 183.70, 181.98, 147.51, 147.48, 146.40, 143.54, 143.49, 142.55, 137.93, 137.85, 136.91, 136.88, 136.72, 133.08, 132.85, 132.23, 131.54, 129.49, 129.29, 128.14, 127.43, 124.41, 112.54, 112.39, 55.36, 39.19, 39.03, 38.97, 38.89, 34.99, 33.84, 33.51, 33.44, 33.22, 33.10, 31.93, 31.83, 31.50, 31.47, 30.37, 30.26, 30.15, 30.05, 29.73, 29.71, 29.62, 29.31, 29.29, 29.26, 28.93, 28.80, 27.96, 26.68, 26.59, 25.23, 23.10, 23.00, 22.77, 22.74, 22.70, 22.69, 22.64, 22.45, 22.42, 22.40, 14.19, 14.13, 14.09, 14.07,

13.94, 13.93, 13.69, 13.67. HRMS (MALDI-TOF) (m/z): $[M]^+$ calculated for $C_{72}H_{104}N_4O_2S_6$, 1248.65; found: 1248.65.

Synthesis of 1D/2D side asymmetric SMAs: BTP-C11-TBO and BTP-BO-TBO

Compound **5** (61.7 mg, 0.05 mmol) / **5’** (62.4 mg, 0.05 mmol) and IC-2F (58.3 mg, 0.25 mmol) were dissolved in chloroform solution (30 mL) and added pyridine (1 mL), followed by degassing with argon several times. The mixture was stirred at room temperature overnight, then the mixture was poured into methanol and filtered. The residue was purified with column chromatography on silica gel using dichloromethane/petroleum ether (1/1, v/v) as the eluent to give a dark blue solid BTP-C11-TBO (78.6 mg, 94.8% yield) and BTP-BO-TBO (77.5 mg, 92.7% yield).

BTP-C11-TBO: ^{1}H NMR (600 MHz, Chloroform-d) δ 9.14 (d, J=18.9 Hz, 2H), 8.55 (ddd, J=19.3, 9.8, 6.4 Hz, 2H), 7.70 (q, J=7.6 Hz, 2H), 7.34 (d, J=5.7 Hz, 2H), 4.99-4.54 (m, 4H), 3.23 (t, J=8.0 Hz, 2H), 2.71 (d, J=6.8 Hz, 2H), 2.26-2.00 (m, 2H), 1.88 (p, J=7.9 Hz, 2H), 1.73 (s, 1H), 1.63-0.72 (m, 64H), 0.90-0.53 (m, 21H). ^{13}C NMR (151 MHz, Chloroform-d) δ 186.10, 185.73, 158.88, 155.25, 153.90, 147.48, 145.17, 144.44, 144.20, 138.44, 137.62, 136.69, 136.02, 135.31, 134.77, 134.51, 133.70, 133.28, 133.13, 126.44, 119.97, 114.96, 114.90, 114.59, 113.79, 113.59, 113.53, 112.57, 112.46, 68.72, 55.70, 39.29, 39.21, 38.96, 34.92, 33.36, 33.03, 31.99, 31.92, 31.60, 31.59, 31.24, 30.48, 29.84, 29.78, 29.72, 29.66, 29.63, 29.52, 29.46, 29.44, 29.40, 29.35, 28.95, 27.98, 27.86, 26.69, 25.42, 25.19, 23.13, 22.89, 22.84, 22.78, 22.73, 22.70, 22.50, 22.48, 22.46, 14.24, 14.15, 14.13, 14.02, 14.00, 13.81, 13.76. HRMS (MALDI-TOF) (m/z): $[M]^+$ calculated for $C_{95}H_{106}F_4N_8O_2S_6$, 1658.67;

found: 1658.67.

BTP-BO-TBO: ^{1}H NMR (600 MHz, Chloroform-d) δ 9.15 (d, *J*=18.0 Hz, 2H), 8.56 (ddd, *J*=23.6, 9.8, 6.3 Hz, 2H), 7.70 (q, *J*=7.1 Hz, 2H), 7.34 (d, *J*=5.4 Hz, 2H), 4.77 (dp, *J*=19.2, 7.2, 6.2 Hz, 4H), 3.20 (d, *J*=7.7 Hz, 2H), 2.71 (d, *J*=6.8 Hz, 2H), 2.11 (t, *J*=9.3 Hz, 2H), 1.72 (s, 2H), 1.49-0.94 (m, 64H), 0.87-0.60 (m, 24H). ^{13}C NMR (151 MHz, Chloroform-d) δ 185.98, 185.72, 158.88, 153.50, 147.49, 145.32, 144.41, 138.44, 135.73, 134.22, 133.67, 133.10, 132.24, 131.67, 126.43, 121.42, 120.09, 115.08, 114.88, 114.57, 113.77, 113.54, 69.54, 68.74, 55.68, 40.07, 39.25, 39.21, 38.94, 34.89, 34.77, 33.56, 33.34, 33.30, 33.00, 31.96, 31.84, 31.58, 31.54, 31.52, 30.53, 30.47, 30.43, 30.38, 29.75, 29.64, 29.63, 29.38, 29.34, 28.93, 28.83, 26.67, 26.57, 23.10, 23.01, 22.99, 22.86, 22.77, 22.71, 22.64, 22.45, 22.42, 14.22, 14.12, 14.11, 14.08, 14.06, 13.98, 13.96, 13.78, 13.76, 13.72. HRMS (MALDI-TOF) (m/z): $[M]^+$ calculated for $C_{96}H_{108}F_4N_8O_2S_6$, 1672.69; found: 1672.69.

DFT calculation

All calculations are performed by ORCA (*version 5.0.4*)[1]. The molecular geometries of S_0 states, S_1 states and anion state were optimized using function of ωB97X-D3 with def2-SVP basis set, time-dependent DFT (TD-DFT) on ωB97X-D3 with def2-SVP basis set and ωB97X-D3 with def2-SVPD basis set, respectively. For the reorganization energy calculation, the S_0 states, S_1 states and anion state were performed under ωB97X-D3 with def2-SVP basis set, time-dependent DFT (TD-DFT) on ωB97X-D3 with def2-SVP basis set and ωB97X-D3 with def2-SVPD basis set, respectively. The local excitation state and charge transfer state was optimized under

BLYP/def2-SV(P) level. The straight and branched alkyl chains were simplified to butyl and isobutyl, respectively, for saving time. Energy levels and molecular orbitals were calculated under B3PW91/def2-TZVP. ESP and molecular dipole moments were performed under ωB97M-V/def2-SVPD. Wavefunction analysis were performed by Multiwfn (*version 3.8 dev branch-2023-Dec-1*)[2] and VMD (*version 1.9.4*) was used for visualization[3].

Measurements and Instruments

^{1}H NMR and ^{13}C NMR spectra were recorded on Bruker AVANCE NEO 600 MHz spectrometer at room temperature (Supplementary Figs. 30-45). High-resolution matrix-assisted laser desorption ionization-time of flight mass spectrometry (MALDI-TOF MS) was performed on the Shimadzu spectrometer (Supplementary Figs. 46-53). Mass spectra were measured on a Shimadzu spectrometer. The thermogravimetric analysis (TGA) was carried out on a Mettler Toledo TGA thermogravimetric analyzer with a thermal balance under the protection of nitrogen. The UV-vis absorption spectra were measured on a Hitachi U-3010 UV-vis spectrophotometer. Cyclic voltammogram (CV) measurements were conducted on a Zahner IM6e electrochemical workstation using sample film-coated glassy carbon as the working electrode, Pt wire as the counter electrode, and Ag/AgCl as the reference electrode in a 0.1 M tetrabutylammonium hexafluorophosphate (Bu_4NPF_6) acetonitrile solution. The ferrocene/ferrocenium (Fc/Fc^+) couple was used as an internal reference.

SCLC mobility measurements

Electron-only devices with the structure of ITO/ZnO/ active layer/PDINN/Ag

and hole-only devices with the structure of ITO/ PEDOT: PSS/active layer/ MoO_3/Ag are used to conduct SCLC measurements. The mobilities were determined by fitting the dark-field current density-voltage curves using the Mott-Gurney relationship, which is described in the following equation:

$$J(V) = \frac{9}{8}\varepsilon_0\varepsilon_r\mu_0\frac{V^2}{L^3} \quad (1)$$

where J is the current density, ε_0 is the permittivity of free space, $\varepsilon_r \approx 3.5$ is the average dielectric constant of the blend film, μ_0 is the zero-field mobility, $V = V_{applied}-V_{built\text{-}in}-V_{series\text{-}resistance}$ (the $V_{built\text{-}in}$ values are 0.2 V and 0 V for the hole-only and the electron-only devices, respectively), $V_{applied}$ is the voltage applied, and $V_{built\text{-}in}$ is the built-in voltage from the relative work function difference between the two electrodes, and $V_{series\text{-}resistance}$ is the voltage caused by the series and contact resistance potential drop. For convenience, the voltage drops caused by the $R_{series\text{-}resistance}$ was ignored, L is the thickness of the active layer. The SCLC region for the measurement of charge mobility could be determined by the [log (J) vs. log (V)] curves fitted at the slope around 2. Thus, the carrier mobilities could be calculated from the slope of the $J^{1/2} \sim V$ curves in the SCLC region.

AFM, TEM，STEM and CA

The atomic force microscopic (AFM) images were acquired using a Bruker Dimension EDGE in tapping mode. Transmission electron microscopy (TEM) images were obtained on a JEOL JEM-1400 transmission electron microscope. Scanning transmission electron microscopy (STEM) images were obtained on a Talos F200X Scanning transmission electron microscope. Contact angle (CA) test was conducted through OCA 25 with water and diiodomethane.

GIWAXS and GISAXS

GIWAXS measurements were carried out with a Xeuss 2.0 SAXS/WAXS

laboratory beamline using a Cu X-ray source (8.05 keV, 1.54 Å) and a Pilatus3R 300 K detector. The incidence angle is 0.2°. The GIWAXS data were obtained at 1W1A Diffuse X-ray Scattering Station, Beijing Synchrotron Radiation Facility (BSRF-1W1A).

Transient absorption spectroscopy (TAS)

Ti: sapphire amplifier (Astrella, Coherent) delivers a laser beam centered at 800 nm with a pulse duration of ~40 fs, a pulse repetition rate of 1 kHz, and a maximum pulse energy of 7 mJ, the output of the amplifier was divided into two pulses, one of which was used to drive an optical parametric amplifier (Topas C, Light Conversion) to obtain the pump beam, the remaining pulse stream was directed into an ultrafast spectroscopic system (HARPIA-TA, Light Conversion) to generate the white light continuum probe beam. In the spectrometer, the pump pulse chopped at 500 Hz frequency was spatially and temporally overlapped with the probe beam on the sample, and the excitation energy of the pump pulse was set to 2 $\mu J/cm^2$ to avoid singlet-singlet annihilation. The film samples for TA measurements were prepared by spin coating the blended materials on thin quartz plates and then annealed at 100 °C for 10 min in a nitrogen atmosphere before measurement.

Single crystal analysis

Single crystals of BTP-DBO, BTP-DTBO and BTP-BO-TBO were cultivated using a solvent diffusion method with methanol/ethanol as a poor solvent and chloroform as a good solvent[4,5]. According to standard procedure, X-ray single crystal data were collected on XtaLAB Synergy-R at 170 K protected by liquid nitrogen, analyzed using Mercury (version 4.0), and deposited at the Cambridge Crystallographic Data Center (CCDC). The electronic coupling simulations were

performed by the DFT on B3LYP/6-31G** level, and the electronic coupling were calculated using Calc_J in CATNIP (version 1.9) [6,7].

Supplementary References

1. Neese F. The ORCA program system. *WIREs Computational Molecular Science* **2,** 73-78 (2011).
2. Lu T, Chen F. Multiwfn: A multifunctional wavefunction analyzer. *Journal of Computational Chemistry* **33**, 580-592 (2011).
3. Humphrey，William, Dalke，Andrew, Schulten，Klaus. VMD: Visual Molecular Dynamics. *Journal of Molecular Graphics* **14**, 33-38 (1996).
4. Gong Y, *et al.* C-shaped ortho-benzodipyrrole-based acceptors with different electronic effects of top substituents for as-cast green-solvent processed high-performance organic solar cells. *Energy & Environmental Science* **17**, 6844-6855 (2024).
5. Wu X, *et al.* Inner Side Chain Modification of Small Molecule Acceptors Enables Lower Energy Loss and High Efficiency of Organic Solar Cells Processed with Non-halogenated Solvents. *Angewandte Chemie International Edition* **64**, e202416016 (2024).
6. Liu F, *et al.* Nonfullerene Acceptor Featuring Unique Self‐Regulation Effect for Organic Solar Cells with 19 % Efficiency. *Angewandte Chemie International Edition* **63**, e202313791 (2023).
7. Luo Z, *et al.* Asymmetric side-chain substitution enables a 3D network acceptor with hydrogen bond assisted crystal packing and enhanced electronic coupling for efficient organic solar cells. *Energy & Environmental Science* **15**, 4601-4611 (2022).
