# Supplementary material for: Asymmetric small-molecule acceptor enables suppressed electron-vibration coupling and minimized driving force for organic solar cells
Source: Nat Commun. 2025 Feb 10;16:1503. doi: 10.1038/s41467-025-56799-6 (PMC11811148; doi:10.1038/s41467-025-56799-6)
Supplement: Supplementary file 2 — Description Of Additional Supplementary File [file 41467_2025_56799_MOESM2_ESM.pdf]

Description of Additional supplementary files

Supplementary data 1: Atomic Coordinates of BTP-BO-TBO

Supplementary data 2: Atomic Coordinates of BTP-C11-TBO

Supplementary data 3: Atomic Coordinates of BTP-DBO

Supplementary data 4: Atomic Coordinates of BTP-DC11

Supplementary data 5: Atomic Coordinates of BTP-DTBO

Supplementary data 6: Atomic Coordinates of PM6_BTP-BO-TBO_111

Supplementary data 7: Atomic Coordinates of PM6_BTP-BO-TBO_23

Supplementary data 8: Atomic Coordinates of PM6_BTP-BO-TBO_311

Supplementary data 9: Atomic Coordinates of PM6_BTP-BO-TBO_41

Supplementary data 10: Atomic Coordinates of PM6_BTP-DBO_1

Supplementary data 11: Atomic Coordinates of PM6_BTP-DBO_2

Supplementary data 12: Atomic Coordinates of PM6_BTP-DTBO_1

Supplementary data 13: Atomic Coordinates of PM6_BTP-DTBO_2

Supplementary data 14: checkcif-BTP-BO-TBO

Supplementary data 15: checkcif-BTP-DBO

Supplementary data 16: checkcif-BTP-DTBO
