# Supplementary material for: Asymmetric small-molecule acceptor enables suppressed electron-vibration coupling and minimized driving force for organic solar cells
Source: Nat Commun. 2025 Feb 10;16:1503. doi: 10.1038/s41467-025-56799-6 (PMC11811148; doi:10.1038/s41467-025-56799-6)

# checkCIF/PLATON report

Structure factors have been supplied for datablock(s) fx618_auto_sq

THIS REPORT IS FOR GUIDANCE ONLY. IF USED AS PART OF A REVIEW PROCEDURE FOR PUBLICATION, IT SHOULD NOT REPLACE THE EXPERTISE OF AN EXPERIENCED CRYSTALLOGRAPHIC REFEREE.

No syntax errors found.   CIF dictionary   Interpreting this report

# Datablock: fx618_auto_sq

---

| | | | |
|---|---|---|---|
| Bond precision: | C-C = 0.0116 A | | Wavelength=1.54184 |
| Cell: | a=28.0817(11) | b=56.7025(17) | c=13.5233(5) |
| | alpha=90 | beta=93.635(4) | gamma=90 |
| Temperature: | 170 K | | |

| | Calculated | Reported |
|---|---|---|
| Volume | 21489.9(13) | 21489.9(13) |
| Space group | C 2/c | C 1 2/c 1 |
| Hall group | -C 2yc | -C 2yc |
| Moiety formula | C85 H60 F4 N8 O2 S6 [+ solvent] | C84.797 H79.491 F4 N8 O2 S6, 0.102(C1), 0.25[] |
| Sum formula | C85 H60 F4 N8 O2 S6 [+ solvent] | C85 H60 F4 N8 O2 S6 |
| Mr | 1493.77 | 1493.77 |
| Dx,g cm-3 | 0.923 | 0.923 |
| Z | 8 | 8 |
| Mu (mm-1) | 1.544 | 1.544 |
| F000 | 6192.0 | 6192.0 |
| F000' | 6224.55 | |
| h,k,lmax | 35,71,17 | 35,71,17 |
| Nref | 22847 | 21460 |
| Tmin,Tmax | 0.631,0.970 | 0.524,1.000 |
| Tmin' | 0.572 | |

Correction method= # Reported T Limits: Tmin=0.524 Tmax=1.000
AbsCorr = MULTI-SCAN

Data completeness= 0.939   Theta(max)= 77.459

R(reflections)= 0.1046( 10551) wR2(reflections)= 0.3410( 21460)

S = 1.077 Npar= 946

---

The following ALERTS were generated. Each ALERT has the format
**test-name_ALERT_alert-type_alert-level**.
Click on the hyperlinks for more details of the test.

---

### Alert level B

```
PLAT220_ALERT_2_B NonSolvent   Resd 1  C   Ueq(max)/Ueq(min) Range       10.0 Ratio
PLAT242_ALERT_2_B Low    'MainMol' Ueq as Compared to Neighbors of        C73 Check
PLAT315_ALERT_2_B Singly Bonded Carbon Detected (H-atoms Missing).        C54 Check
PLAT315_ALERT_2_B Singly Bonded Carbon Detected (H-atoms Missing).        C58 Check
PLAT315_ALERT_2_B Singly Bonded Carbon Detected (H-atoms Missing).        C64 Check
PLAT315_ALERT_2_B Singly Bonded Carbon Detected (H-atoms Missing).        C68 Check
PLAT315_ALERT_2_B Singly Bonded Carbon Detected (H-atoms Missing).        C74 Check
PLAT315_ALERT_2_B Singly Bonded Carbon Detected (H-atoms Missing).        C78 Check
PLAT315_ALERT_2_B Singly Bonded Carbon Detected (H-atoms Missing).        C84 Check
PLAT315_ALERT_2_B Singly Bonded Carbon Detected (H-atoms Missing).        C85 Check
PLAT340_ALERT_3_B Low Bond Precision on  C-C Bonds ..............     0.0116 Ang.
PLAT410_ALERT_2_B Short Intra H...H Contact  H59B     ..H69B     .      1.85 Ang.
                                                    x,y,z  =      1_555 Check
```

---

### Alert level C

```
PLAT026_ALERT_3_C Ratio Observed / Unique Reflections (too) Low ..        49% Check
PLAT084_ALERT_3_C High wR2 Value (i.e. > 0.25) ..................       0.34 Report
PLAT222_ALERT_3_C NonSolvent Resd 1  H   Uiso(max)/Uiso(min) Range       6.9 Ratio
PLAT230_ALERT_2_C Hirshfeld Test Diff for    C4      --C5       .        6.1 s.u.
PLAT230_ALERT_2_C Hirshfeld Test Diff for    C38     --C46      .        5.7 s.u.
PLAT230_ALERT_2_C Hirshfeld Test Diff for    C76     --C77      .        5.6 s.u.
PLAT234_ALERT_4_C Large Hirshfeld Difference F1      --C6       .       0.17 Ang.
PLAT234_ALERT_4_C Large Hirshfeld Difference C50     --C55      .       0.24 Ang.
PLAT234_ALERT_4_C Large Hirshfeld Difference C62     --C63      .       0.18 Ang.
PLAT234_ALERT_4_C Large Hirshfeld Difference C65     --C66      .       0.20 Ang.
PLAT234_ALERT_4_C Large Hirshfeld Difference C72     --C73      .       0.16 Ang.
PLAT234_ALERT_4_C Large Hirshfeld Difference C75     --C76      .       0.23 Ang.
PLAT234_ALERT_4_C Large Hirshfeld Difference C77     --C78      .       0.22 Ang.
PLAT234_ALERT_4_C Large Hirshfeld Difference C79     --C80      .       0.21 Ang.
PLAT241_ALERT_2_C High   'MainMol' Ueq as Compared to Neighbors of         S6 Check
PLAT241_ALERT_2_C High   'MainMol' Ueq as Compared to Neighbors of        C34 Check
PLAT241_ALERT_2_C High   'MainMol' Ueq as Compared to Neighbors of        C56 Check
PLAT241_ALERT_2_C High   'MainMol' Ueq as Compared to Neighbors of        C57 Check
PLAT241_ALERT_2_C High   'MainMol' Ueq as Compared to Neighbors of        C72 Check
PLAT241_ALERT_2_C High   'MainMol' Ueq as Compared to Neighbors of        C77 Check
PLAT242_ALERT_2_C Low    'MainMol' Ueq as Compared to Neighbors of        C33 Check
PLAT242_ALERT_2_C Low    'MainMol' Ueq as Compared to Neighbors of        C49 Check
PLAT242_ALERT_2_C Low    'MainMol' Ueq as Compared to Neighbors of        C50 Check
PLAT242_ALERT_2_C Low    'MainMol' Ueq as Compared to Neighbors of        C53 Check
PLAT242_ALERT_2_C Low    'MainMol' Ueq as Compared to Neighbors of        C61 Check
PLAT242_ALERT_2_C Low    'MainMol' Ueq as Compared to Neighbors of        C67 Check
PLAT242_ALERT_2_C Low    'MainMol' Ueq as Compared to Neighbors of        C70 Check
PLAT242_ALERT_2_C Low    'MainMol' Ueq as Compared to Neighbors of        C79 Check
PLAT242_ALERT_2_C Low    'MainMol' Ueq as Compared to Neighbors of        C80 Check
```

```
PLAT242_ALERT_2_C Low    'MainMol' Ueq as Compared to Neighbors of        C83 Check
PLAT260_ALERT_2_C Large Average Ueq of Residue Including        S1      0.159 Check
PLAT905_ALERT_3_C Negative K value in the Analysis of Variance ...     -3.176 Report
PLAT911_ALERT_3_C Missing FCF Refl Between Thmin & STh/L=    0.600        153 Report
                 5  1  0,   4  4  0,   4 10  0,   2 12  0, -29  1  1,   7  1  1,
                 1  3  1,   5  3  1, -10  4  1,   1  5  1,   0  8  1,   0 10  1,
                29 33  1,  28 36  1,  27 37  1,  27 39  1,  26 42  1, -16  0  2,
               -15  1  2, -16  2  2, -15  3  2,  -1  3  2, -16  4  2, -15  5  2,
               -16  6  2, -16  8  2,  -6 10  2,   8 10  2,   9 17  2, -31 25  2,
                24 46  2, -17 49  2, -16 50  2, -16 52  2,  18 54  2,  17 55  2,
                18 56  2, -15  7  3, -15  9  3,  -1  9  3,   1  9  3,  -1 11  3,
                23 47  3, -17 51  3, -16 52  3,  18 52  3,  18 54  3,  -5  5  4,
                 1  9  4,  23 25  4,  23 27  4, -21 37  4, -20 40  4,  18 52  4,
                18 54  4, -10 62  4,  -9 63  4,  -8 64  4, -30 24  5,  18 52  5,
                17 49  6,  17 51  6,   5 61  6,  29  1  7,  29  3  7,  29  5  7,
                16 48  7,  17 49  7,  16 50  7, -30  2  8, -29 15  8, -27 15  8,
               -28 16  8, -29 17  8, -28 18  8, -20 40  8, -21 41  8, -20 42  8,
               -21 43  8,  15 47  8,  16 48  8, -29  1  9, -27 11  9, -26 12  9,
               -27 13  9, -28 14  9, -26 14  9, -27 15  9, -28 16  9,  13 33  9,
                13 35  9,  14 36  9,  13 37  9, -20 38  9,  14 38  9,  13 39  9,
PLAT918_ALERT_3_C Reflection(s) with I(obs) much Smaller I(calc) .          2 Check
PLAT977_ALERT_2_C Check Negative Difference Density on H56A      .      -0.33 eA-3
```

---

## Alert level G

```
FORMU01_ALERT_1_G  There is a discrepancy between the atom counts in the
            _chemical_formula_sum and _chemical_formula_moiety. This is
            usually due to the moiety formula being in the wrong format.
            Atom count from _chemical_formula_sum:   C85 H60 F4 N8 O2 S6
            Atom count from _chemical_formula_moiety:C84.89899 H79.49099 F4 N8 O2
PLAT002_ALERT_2_G Number of Distance or Angle Restraints on AtSite         36 Note
PLAT003_ALERT_2_G Number of Uiso or U(i,j)  Restrained non-H-Atoms         36 Report
PLAT042_ALERT_1_G Calc. and Reported MoietyFormula Strings  Differ     Please Check
              Calc: C85 H60 F4 N8 O2 S6
              Rep.: C84.797 H79.491 F4 N8 O2 S6, 0.102(C1), 0.25[]
PLAT072_ALERT_2_G SHELXL First  Parameter in WGHT  Unusually Large       0.20 Report
PLAT172_ALERT_4_G The CIF-Embedded .res File Contains DFIX Records          1 Report
PLAT177_ALERT_4_G The CIF-Embedded .res File Contains DELU Records          4 Report
PLAT178_ALERT_4_G The CIF-Embedded .res File Contains SIMU Records          4 Report
PLAT188_ALERT_3_G A Non-default SIMU Restraint Value has been used     0.0200 Report
PLAT188_ALERT_3_G A Non-default SIMU Restraint Value has been used     0.0200 Report
PLAT188_ALERT_3_G A Non-default SIMU Restraint Value has been used     0.0200 Report
PLAT188_ALERT_3_G A Non-default SIMU Restraint Value has been used     0.0200 Report
PLAT333_ALERT_2_G Large Aver C6-Ring C-C Dist C20      -C25      .       1.42 Ang.
PLAT343_ALERT_2_G Unusual sp?      Angle Range in Main Residue for        C54 Check
PLAT343_ALERT_2_G Unusual sp3      Angle Range in Main Residue for        C55 Check
PLAT343_ALERT_2_G Unusual sp3      Angle Range in Main Residue for        C56 Check
PLAT343_ALERT_2_G Unusual sp3      Angle Range in Main Residue for        C57 Check
PLAT343_ALERT_2_G Unusual sp?      Angle Range in Main Residue for        C58 Check
PLAT343_ALERT_2_G Unusual sp?      Angle Range in Main Residue for        C64 Check
PLAT343_ALERT_2_G Unusual sp?      Angle Range in Main Residue for        C68 Check
PLAT343_ALERT_2_G Unusual sp?      Angle Range in Main Residue for        C74 Check
PLAT343_ALERT_2_G Unusual sp?      Angle Range in Main Residue for        C77 Check
PLAT343_ALERT_2_G Unusual sp?      Angle Range in Main Residue for        C78 Check
PLAT343_ALERT_2_G Unusual sp3      Angle Range in Main Residue for        C83 Check
PLAT343_ALERT_2_G Unusual sp?      Angle Range in Main Residue for        C84 Check
PLAT343_ALERT_2_G Unusual sp?      Angle Range in Main Residue for        C85 Check
```

```
PLAT367_ALERT_2_G Long?  C(sp?)-C(sp?) Bond  C53      - C54      .       1.52 Ang.
PLAT367_ALERT_2_G Long?  C(sp?)-C(sp?) Bond  C57      - C58      .       1.55 Ang.
PLAT367_ALERT_2_G Long?  C(sp?)-C(sp?) Bond  C63      - C64      .       1.55 Ang.
PLAT367_ALERT_2_G Long?  C(sp?)-C(sp?) Bond  C67      - C68      .       1.55 Ang.
PLAT367_ALERT_2_G Long?  C(sp?)-C(sp?) Bond  C73      - C74      .       1.53 Ang.
PLAT367_ALERT_2_G Long?  C(sp?)-C(sp?) Bond  C76      - C77      .       1.53 Ang.
PLAT367_ALERT_2_G Long?  C(sp?)-C(sp?) Bond  C77      - C78      .       1.55 Ang.
PLAT367_ALERT_2_G Long?  C(sp?)-C(sp?) Bond  C80      - C85      .       1.55 Ang.
PLAT367_ALERT_2_G Long?  C(sp?)-C(sp?) Bond  C83      - C84      .       1.56 Ang.
PLAT432_ALERT_2_G Short Inter X...Y Contact  C77      ..C77      .       3.17 Ang.
                                                1-x,y,1/2-z  =      2_655 Check
PLAT606_ALERT_4_G Solvent Accessible VOID(S) in Structure ........          ! Info
PLAT793_ALERT_4_G Model has Chirality at C70         (Centro SpGr)          R Verify
PLAT793_ALERT_4_G Model has Chirality at C80         (Centro SpGr)          R Verify
PLAT860_ALERT_3_G Number of Least-Squares Restraints .............        224 Note
PLAT869_ALERT_4_G ALERTS Related to the Use of SQUEEZE  Suppressed          ! Info
PLAT910_ALERT_3_G Missing # of FCF Reflection(s) Below Theta(Min).          2 Note
                1  1  0,   0  2  0,
PLAT912_ALERT_4_G Missing # of FCF Reflections Above STh/L=  0.600       1227 Note
PLAT933_ALERT_2_G Number of HKL-OMIT Records in Embedded .res File         13 Note
              -10  4  1,  -6 10  2,  -5  5  4,  -1  3  2,   1  3  1,   1  5  1,
                1  9  3,   1  9  4,   2 12  0,   4  4  0,   4 10  0,   5  1  0,
                8 10  2,
PLAT941_ALERT_3_G Average HKL Measurement Multiplicity ...........        3.3 Low
PLAT969_ALERT_5_G The 'Henn et al.' R-Factor-gap value ...........      4.631 Note
              Predicted wR2: Based on SigI**2  7.36 or SHELX Weight 31.78
PLAT978_ALERT_2_G Number C-C Bonds with Positive Residual Density.          0 Info
PLAT992_ALERT_5_G Repd & Actual _reflns_number_gt Values Differ by          3 Check
```

---

```
   0 ALERT level A = Most likely a serious problem - resolve or explain
  12 ALERT level B = A potentially serious problem, consider carefully
  35 ALERT level C = Check. Ensure it is not caused by an omission or oversight
  48 ALERT level G = General information/check it is not something unexpected

   2 ALERT type 1 CIF construction/syntax error, inconsistent or missing data
  61 ALERT type 2 Indicator that the structure model may be wrong or deficient
  14 ALERT type 3 Indicator that the structure quality may be low
  16 ALERT type 4 Improvement, methodology, query or suggestion
   2 ALERT type 5 Informative message, check
```

---

It is advisable to attempt to resolve as many as possible of the alerts in all categories. Often the minor alerts point to easily fixed oversights, errors and omissions in your CIF or refinement strategy, so attention to these fine details can be worthwhile. In order to resolve some of the more serious problems it may be necessary to carry out additional measurements or structure refinements. However, the purpose of your study may justify the reported deviations and the more serious of these should normally be commented upon in the discussion or experimental section of a paper or in the "special_details" fields of the CIF. checkCIF was carefully designed to identify outliers and unusual parameters, but every test has its limitations and alerts that are not important in a particular case may appear. Conversely, the absence of alerts does not guarantee there are no aspects of the results needing attention. It is up to the individual to critically assess their own results and, if necessary, seek expert advice.

**Publication of your CIF in IUCr journals**

A basic structural check has been run on your CIF. These basic checks will be run on all CIFs submitted for publication in IUCr journals (*Acta Crystallographica*, *Journal of Applied Crystallography*, *Journal of Synchrotron Radiation*); however, if you intend to submit to *Acta Crystallographica Section C* or *E* or *IUCrData*, you should make sure that full publication checks are run on the final version of your CIF prior to submission.

**Publication of your CIF in other journals**

Please refer to the *Notes for Authors* of the relevant journal for any special instructions relating to CIF submission.

---

**PLATON version of 11/11/2024; check.def file version of 11/11/2024**

**Datablock fx618_auto_sq** - ellipsoid plot

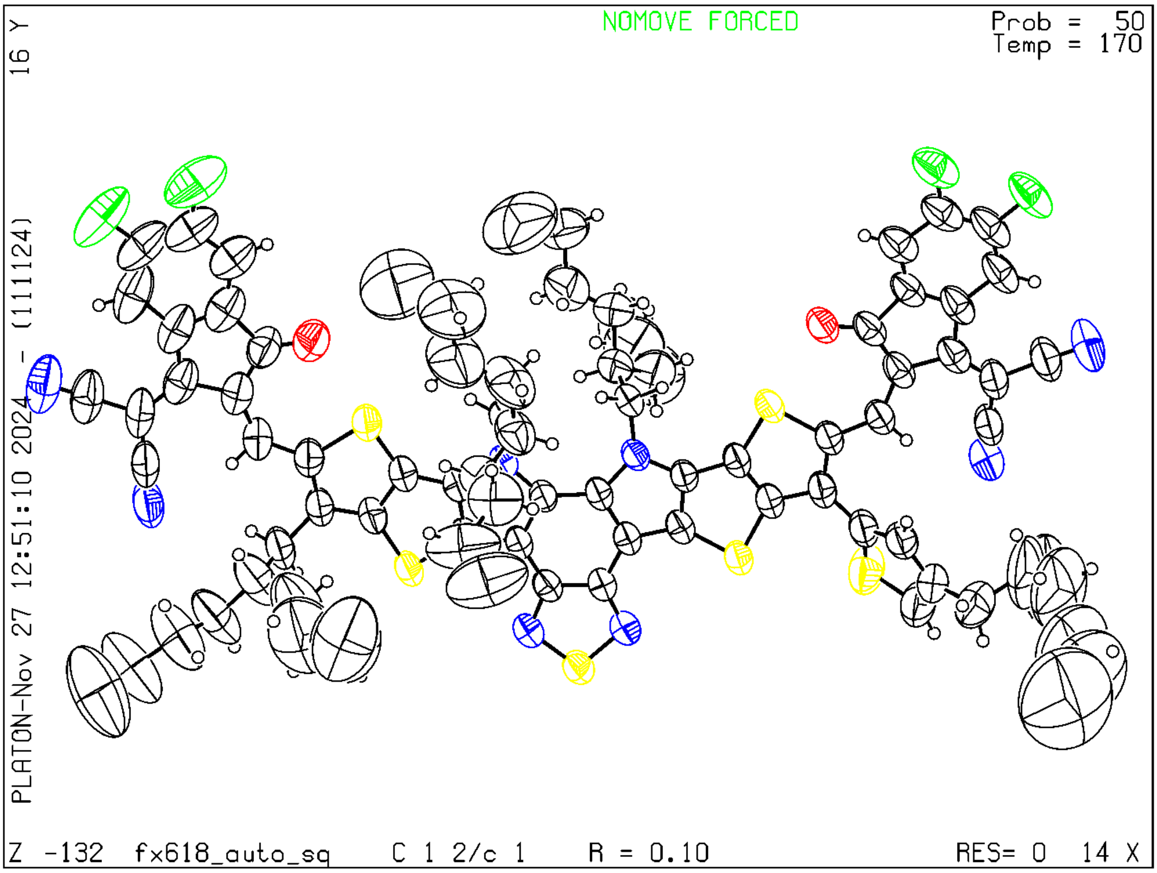

Supplement: Supplementary file 16 — Supplementary Data 14 [file 41467_2025_56799_MOESM16_ESM.pdf]
