# Supplementary material for: Asymmetric small-molecule acceptor enables suppressed electron-vibration coupling and minimized driving force for organic solar cells
Source: Nat Commun. 2025 Feb 10;16:1503. doi: 10.1038/s41467-025-56799-6 (PMC11811148; doi:10.1038/s41467-025-56799-6)

checkCIF/PLATON report

Structure factors have been supplied for datablock(s) fx449_auto_sq

THIS REPORT IS FOR GUIDANCE ONLY. IF USED AS PART OF A REVIEW PROCEDURE FOR PUBLICATION, IT SHOULD NOT REPLACE THE EXPERTISE OF AN EXPERIENCED CRYSTALLOGRAPHIC REFEREE.

No syntax errors found. CIF dictionary Interpreting this report

Datablock: fx449_auto_sq

Bond precision:	C-C = 0.0181 A	Wavelength=1.54184	
Cell:	a=27.0612(7)	b=22.3128(6)	c=32.2993(9)
	alpha=90	beta=110.561(3)	gamma=90
Temperature:	170 K		

	Calculated	Reported
Volume	18260.3(9)	18260.3(9)
Space group	C 2/c	C 1 2/c 1
Hall group	-C 2yc	-C 2yc
Moiety formula	C92 H93 F4 N8 O2 S5, C H Cl2, Cl [+ solvent]	0.039(C H Cl3), 0.039(C91 H103 F4 N8 O2 S5), 0.02[], 0.039[], 0
Sum formula	C93 H94 Cl3 F4 N8 O2 S5 [+ solvent]	C93 H94 Cl3 F4 N8 O2 S5
Mr	1698.42	1698.41
Dx,g cm-3	1.236	1.236
Z	8	8
Mu (mm-1)	2.452	2.452
F000	7128.0	7128.0
F000'	7167.79	
h,k,lmax	32,26,38	32,26,38
Nref	16621	16521
Tmin,Tmax	0.863,0.907	0.472,1.000
Tmin'	0.332	

Correction method= # Reported T Limits: Tmin=0.472 Tmax=1.000
AbsCorr = MULTI-SCAN

Data completeness= 0.994 Theta(max)= 68.000

R(reflections)= 0.1508(10054) wR2(reflections)= 0.3999(16521)

S = 1.032 Npar= 1035

The following ALERTS were generated. Each ALERT has the format
test-name_ALERT_alert-type_alert-level.
Click on the hyperlinks for more details of the test.

Alert level B

```
PLAT084_ALERT_3_B High wR2 Value (i.e. > 0.25) ..................       0.40 Report
PLAT220_ALERT_2_B NonSolvent   Resd 1  C   Ueq(max)/Ueq(min)  Range        6.1 Ratio
PLAT230_ALERT_2_B Hirshfeld Test Diff for    C82       --C83      .        7.3 s.u.
PLAT234_ALERT_4_B Large Hirshfeld Difference C62       --C63      .       0.26 Ang.
PLAT234_ALERT_4_B Large Hirshfeld Difference C71       --C72      .       0.29 Ang.
PLAT234_ALERT_4_B Large Hirshfeld Difference C72       --C73      .       0.26 Ang.
PLAT234_ALERT_4_B Large Hirshfeld Difference C83       --C84      .       0.26 Ang.
PLAT234_ALERT_4_B Large Hirshfeld Difference C91       --C92      .       0.30 Ang.
PLAT241_ALERT_2_B High   'MainMol' Ueq as Compared to Neighbors of         C54 Check
PLAT260_ALERT_2_B Large Average Ueq of Residue Including       Cl2      0.313 Check
PLAT260_ALERT_2_B Large Average Ueq of Residue Including       Cl1      0.349 Check
PLAT315_ALERT_2_B Singly Bonded Carbon Detected (H-atoms Missing).         C51 Check
PLAT315_ALERT_2_B Singly Bonded Carbon Detected (H-atoms Missing).         C74 Check
PLAT315_ALERT_2_B Singly Bonded Carbon Detected (H-atoms Missing).         C80 Check
PLAT340_ALERT_3_B Low Bond Precision on  C-C Bonds ..............    0.01807 Ang.
PLAT410_ALERT_2_B Short Intra H...H Contact  H46       ..H54A     .       1.81 Ang.
                                                       x,y,z  =      1_555 Check
PLAT410_ALERT_2_B Short Intra H...H Contact  H58A      ..H69B     .       1.88 Ang.
                                                       x,y,z  =      1_555 Check
PLAT410_ALERT_2_B Short Intra H...H Contact  H70       ..H71B     .       1.89 Ang.
                                                       x,y,z  =      1_555 Check
PLAT410_ALERT_2_B Short Intra H...H Contact  H70       ..H75A     .       1.82 Ang.
                                                       x,y,z  =      1_555 Check
PLAT413_ALERT_2_B Short Inter XH3 .. XHn     H55A      ..H86C     .       1.95 Ang.
                                          3/2-x,1/2+y,3/2-z  =      4_656 Check
PLAT413_ALERT_2_B Short Inter XH3 .. XHn     H84B      ..H86B     .       1.97 Ang.
                                                 2-x,1-y,2-z  =      5_767 Check
```

Alert level C

```
PLAT082_ALERT_2_C High R1 Value .................................       0.15 Report
PLAT222_ALERT_3_C NonSolvent Resd 1  H   Uiso(max)/Uiso(min)  Range        6.4 Ratio
PLAT230_ALERT_2_C Hirshfeld Test Diff for    C33       --C41      .        5.1 s.u.
PLAT230_ALERT_2_C Hirshfeld Test Diff for    C48       --C49      .        5.2 s.u.
PLAT230_ALERT_2_C Hirshfeld Test Diff for    C60       --C61      .        5.2 s.u.
PLAT230_ALERT_2_C Hirshfeld Test Diff for    C67       --C68      .        5.5 s.u.
PLAT234_ALERT_4_C Large Hirshfeld Difference F3        --C37      .       0.24 Ang.
PLAT234_ALERT_4_C Large Hirshfeld Difference N8        --C44      .       0.16 Ang.
PLAT234_ALERT_4_C Large Hirshfeld Difference C34       --C35      .       0.17 Ang.
PLAT234_ALERT_4_C Large Hirshfeld Difference C34       --C42      .       0.20 Ang.
PLAT234_ALERT_4_C Large Hirshfeld Difference C35       --C40      .       0.22 Ang.
PLAT234_ALERT_4_C Large Hirshfeld Difference C37       --C38      .       0.24 Ang.
PLAT234_ALERT_4_C Large Hirshfeld Difference C47       --C48      .       0.20 Ang.
PLAT234_ALERT_4_C Large Hirshfeld Difference C52       --C53      .       0.21 Ang.
PLAT234_ALERT_4_C Large Hirshfeld Difference C59       --C60      .       0.19 Ang.
```

```
PLAT234_ALERT_4_C Large Hirshfeld Difference C61      --C62      .       0.22 Ang.
PLAT234_ALERT_4_C Large Hirshfeld Difference C64      --C65      .       0.18 Ang.
PLAT234_ALERT_4_C Large Hirshfeld Difference C65      --C66      .       0.18 Ang.
PLAT234_ALERT_4_C Large Hirshfeld Difference C70      --C75      .       0.22 Ang.
PLAT234_ALERT_4_C Large Hirshfeld Difference C81      --C82      .       0.21 Ang.
PLAT241_ALERT_2_C High   'MainMol' Ueq as Compared to Neighbors of         S3 Check
PLAT241_ALERT_2_C High   'MainMol' Ueq as Compared to Neighbors of        C55 Check
PLAT241_ALERT_2_C High   'MainMol' Ueq as Compared to Neighbors of        C58 Check
PLAT241_ALERT_2_C High   'MainMol' Ueq as Compared to Neighbors of        C61 Check
PLAT241_ALERT_2_C High   'MainMol' Ueq as Compared to Neighbors of        C64 Check
PLAT241_ALERT_2_C High   'MainMol' Ueq as Compared to Neighbors of        C77 Check
PLAT241_ALERT_2_C High   'MainMol' Ueq as Compared to Neighbors of        C78 Check
PLAT241_ALERT_2_C High   'MainMol' Ueq as Compared to Neighbors of        C83 Check
PLAT242_ALERT_2_C Low    'MainMol' Ueq as Compared to Neighbors of         N5 Check
PLAT242_ALERT_2_C Low    'MainMol' Ueq as Compared to Neighbors of        C38 Check
PLAT242_ALERT_2_C Low    'MainMol' Ueq as Compared to Neighbors of        C43 Check
PLAT242_ALERT_2_C Low    'MainMol' Ueq as Compared to Neighbors of        C44 Check
PLAT242_ALERT_2_C Low    'MainMol' Ueq as Compared to Neighbors of        C48 Check
PLAT242_ALERT_2_C Low    'MainMol' Ueq as Compared to Neighbors of        C52 Check
PLAT242_ALERT_2_C Low    'MainMol' Ueq as Compared to Neighbors of        C53 Check
PLAT242_ALERT_2_C Low    'MainMol' Ueq as Compared to Neighbors of        C60 Check
PLAT242_ALERT_2_C Low    'MainMol' Ueq as Compared to Neighbors of        C65 Check
PLAT242_ALERT_2_C Low    'MainMol' Ueq as Compared to Neighbors of        C67 Check
PLAT242_ALERT_2_C Low    'MainMol' Ueq as Compared to Neighbors of        C68 Check
PLAT242_ALERT_2_C Low    'MainMol' Ueq as Compared to Neighbors of        C73 Check
PLAT242_ALERT_2_C Low    'MainMol' Ueq as Compared to Neighbors of        C76 Check
PLAT242_ALERT_2_C Low    'MainMol' Ueq as Compared to Neighbors of        C81 Check
PLAT242_ALERT_2_C Low    'MainMol' Ueq as Compared to Neighbors of        C84 Check
PLAT242_ALERT_2_C Low    'MainMol' Ueq as Compared to Neighbors of        C90 Check
PLAT242_ALERT_2_C Low    'MainMol' Ueq as Compared to Neighbors of        C91 Check
PLAT260_ALERT_2_C Large Average Ueq of Residue Including        S1      0.201 Check
PLAT410_ALERT_2_C Short Intra H...H Contact  H59      ..H62B     .       1.94 Ang.
                                                  x,y,z  =      1_555 Check
PLAT411_ALERT_2_C Short Inter H...H Contact  H55B     ..H85B     .       2.03 Ang.
                                      3/2-x,1/2+y,3/2-z  =      4_656 Check
PLAT411_ALERT_2_C Short Inter H...H Contact  H73A     ..H93      .       2.11 Ang.
                                     3/2-x,-1/2+y,3/2-z  =      4_646 Check
PLAT906_ALERT_3_C Large K Value in the Analysis of Variance ......      6.071 Check
PLAT906_ALERT_3_C Large K Value in the Analysis of Variance ......      2.324 Check
PLAT911_ALERT_3_C Missing FCF Refl Between Thmin & STh/L=    0.600         94 Report
                0  2  0,   2  0  0,   5  5  0,  24 16  0,  25 15  0, -25 15  1,
               -3  5  1,   2  2  1,  24 16  1,   0  2  2,   1  1  2,   2  0  2,
                2  4  2,   5  5  2,  -8  4  3,  -6  4  3,  -4  4  3,  -3  5  3,
               -2  2  3,   0  2  3,   1  5  3,   3  1  3,   6  4  3,  10  2  3,
               -2 26  4,  -1  1  4,   0  0  4,   0  2  4,   1  1  4,   1  3  4,
               25 13  4, -10 24  5,  -9 25  5,  -4 26  5,  -3 13  5,  -2 12  5,
               -1 13  5,   1  3  5,   9  3  5,  13 23  5, -10 24  6,  -9 25  6,
               -4 26  6,  -3 13  6,  -1  3  6,   1  3  6,   2  2  6,   6  0  6,
               13 23  6, -28 12  7, -27 13  7, -10 24  7,  -9 25  7,  -2  2  7,
               -1  7  7,   1  3  7, -29 11  8, -27 13  8,  -4  8  8,   1  3  8,
                2  0  8, -29 11  9,  -4  4  9,   1  3  9, -30 10 10,  -1  1 10,
               22 10 10,  19 11 11,  19 11 12,   8  0 14, -20 20 15,   7 19 19,
                8 20 19,   6 20 20,   7 19 20, -10 22 21, -12 18 22, -11 19 22,
                1 19 25,   0 18 26, -23  9 27,   7 11 29,   8 10 29,   0  0 30,
                1  3 30,   6 10 30,   7  9 30,   4 10 31, -17  1 38, -16  0 38,
              -15  1 38, -14  0 38, -14  2 38, -13  3 38,
PLAT918_ALERT_3_C Reflection(s) with I(obs) much Smaller I(calc) .          8 Check
```

```
PLAT977_ALERT_2_C Check Negative Difference Density on H54B      .      -0.40 eA-3
PLAT977_ALERT_2_C Check Negative Difference Density on H56A      .      -0.32 eA-3
```

Alert level G

```
FORMU01_ALERT_1_G  There is a discrepancy between the atom counts in the
            _chemical_formula_sum and _chemical_formula_moiety. This is
            usually due to the moiety formula being in the wrong format.
            Atom count from _chemical_formula_sum:   C93 H94 Cl3 F4 N8 O2 S5
            Atom count from _chemical_formula_moiety:C3.588 H4.056 Cl0.117 F0.156
PLAT002_ALERT_2_G Number of Distance or Angle Restraints on AtSite         48 Note
PLAT003_ALERT_2_G Number of Uiso or U(i,j)  Restrained non-H-Atoms         48 Report
PLAT042_ALERT_1_G Calc. and Reported MoietyFormula Strings  Differ     Please Check
              Calc: C92 H93 F4 N8 O2 S5, C H Cl2, Cl
              Rep.: 0.039(C H Cl3), 0.039(C91 H103 F4 N8 O2 S5), 0
                    .02[], 0.039[], 0.02[], 0.02[]
PLAT072_ALERT_2_G SHELXL First  Parameter in WGHT  Unusually Large       0.17 Report
PLAT083_ALERT_2_G SHELXL Second Parameter in WGHT  Unusually Large      60.52 Why ?
PLAT172_ALERT_4_G The CIF-Embedded .res File Contains DFIX Records          2 Report
PLAT173_ALERT_4_G The CIF-Embedded .res File Contains DANG Records         11 Report
PLAT177_ALERT_4_G The CIF-Embedded .res File Contains DELU Records          4 Report
PLAT178_ALERT_4_G The CIF-Embedded .res File Contains SIMU Records          4 Report
PLAT188_ALERT_3_G A Non-default SIMU Restraint Value has been used     0.0200 Report
PLAT188_ALERT_3_G A Non-default SIMU Restraint Value has been used     0.0200 Report
PLAT188_ALERT_3_G A Non-default SIMU Restraint Value has been used     0.0200 Report
PLAT188_ALERT_3_G A Non-default SIMU Restraint Value has been used     0.0200 Report
PLAT333_ALERT_2_G Large Aver C6-Ring C-C Dist C20      -C25      .       1.42 Ang.
PLAT335_ALERT_2_G Check Large C6 Ring C-C Range C35      -C40            0.18 Ang.
PLAT343_ALERT_2_G Unusual sp?      Angle Range in Main Residue for        C51 Check
PLAT343_ALERT_2_G Unusual sp3      Angle Range in Main Residue for        C59 Check
PLAT343_ALERT_2_G Unusual sp3      Angle Range in Main Residue for        C69 Check
PLAT343_ALERT_2_G Unusual sp?      Angle Range in Main Residue for        C70 Check
PLAT343_ALERT_2_G Unusual sp?      Angle Range in Main Residue for        C74 Check
PLAT343_ALERT_2_G Unusual sp3      Angle Range in Main Residue for        C75 Check
PLAT343_ALERT_2_G Unusual sp?      Angle Range in Main Residue for        C78 Check
PLAT343_ALERT_2_G Unusual sp?      Angle Range in Main Residue for        C79 Check
PLAT343_ALERT_2_G Unusual sp?      Angle Range in Main Residue for        C80 Check
PLAT344_ALERT_2_G Unusual sp?       Angle Range in Solvent/Ion for        C93 Check
PLAT367_ALERT_2_G Long?  C(sp?)-C(sp?) Bond  C51      - C68      .       1.54 Ang.
PLAT367_ALERT_2_G Long?  C(sp?)-C(sp?) Bond  C70      - C71      .       1.54 Ang.
PLAT367_ALERT_2_G Long?  C(sp?)-C(sp?) Bond  C73      - C74      .       1.54 Ang.
PLAT367_ALERT_2_G Long?  C(sp?)-C(sp?) Bond  C77      - C78      .       1.55 Ang.
PLAT367_ALERT_2_G Long?  C(sp?)-C(sp?) Bond  C78      - C79      .       1.54 Ang.
PLAT367_ALERT_2_G Long?  C(sp?)-C(sp?) Bond  C79      - C80      .       1.54 Ang.
PLAT431_ALERT_2_G Short Inter HL..A Contact  Cl2      ..O2       .       3.05 Ang.
                                        3/2-x,1/2+y,3/2-z  =     4_656 Check
PLAT432_ALERT_2_G Short Inter X...Y Contact  Cl1      ..C93      .       2.08 Ang.
                                                    x,y,z  =     1_555 Check
PLAT432_ALERT_2_G Short Inter X...Y Contact  Cl3      ..C62      .       3.21 Ang.
                                              x,1-y,1/2+z  =     6_566 Check
PLAT432_ALERT_2_G Short Inter X...Y Contact  C51      ..C61      .       2.64 Ang.
                                          3/2-x,1/2-y,1-z  =     7_656 Check
PLAT432_ALERT_2_G Short Inter X...Y Contact  C73      ..C80      .       2.90 Ang.
                                       3/2-x,-1/2+y,3/2-z  =     4_646 Check
PLAT432_ALERT_2_G Short Inter X...Y Contact  C74      ..C80      .       2.86 Ang.
                                       3/2-x,-1/2+y,3/2-z  =     4_646 Check
PLAT434_ALERT_2_G Short Inter HL..HL Contact Cl1      ..Cl3      .       2.79 Ang.
```

```
                                                     x,y,z  =      1_555 Check
PLAT434_ALERT_2_G Short Inter HL..HL Contact Cl1      ..Cl2      .       2.93 Ang.
                                                     x,y,z  =      1_555 Check
PLAT605_ALERT_4_G Largest Solvent Accessible VOID in the Structure       350 A**3
PLAT793_ALERT_4_G Model has Chirality at C46        (Centro SpGr)          S Verify
PLAT793_ALERT_4_G Model has Chirality at C59        (Centro SpGr)          S Verify
PLAT793_ALERT_4_G Model has Chirality at C82        (Centro SpGr)          S Verify
PLAT860_ALERT_3_G Number of Least-Squares Restraints ............        323 Note
PLAT869_ALERT_4_G ALERTS Related to the Use of SQUEEZE  Suppressed          ! Info
PLAT912_ALERT_4_G Missing # of FCF Reflections Above STh/L=  0.600          4 Note
PLAT933_ALERT_2_G Number of HKL-OMIT Records in Embedded .res File         29 Note
               -8  4  3,  -6  4  3,  -4  4  3,  -4  4  9,  -4  8  8,  -3  5  1,
               -3  5  3,  -2  2  3,  -2  2  7,  -1  1  4,  -1  1 10,  -1  3  6,
                0  0  4,   1  1  2,   1  3  4,   1  5  3,   2  0  0,   2  0  2,
                2  0  8,   2  2  1,   2  2  6,   2  4  2,   3  1  3,   5  5  0,
                5  5  2,   6  0  6,   6  4  3,   9  3  5,  10  2  3,
PLAT941_ALERT_3_G Average HKL Measurement Multiplicity ..........        3.9 Low
PLAT967_ALERT_5_G Note: Two-Theta Cutoff Value in Embedded .res ..      136.0 Degree
PLAT969_ALERT_5_G The 'Henn et al.' R-Factor-gap value ..........      5.589 Note
              Predicted wR2: Based on SigI**2  7.16 or SHELX Weight 38.79
PLAT978_ALERT_2_G Number C-C Bonds with Positive Residual Density.          0 Info
PLAT992_ALERT_5_G Repd & Actual _reflns_number_gt Values Differ by         10 Check
```

---

0 **ALERT level A** = Most likely a serious problem - resolve or explain
21 **ALERT level B** = A potentially serious problem, consider carefully
55 **ALERT level C** = Check. Ensure it is not caused by an omission or oversight
53 **ALERT level G** = General information/check it is not something unexpected

2 ALERT type 1 CIF construction/syntax error, inconsistent or missing data
82 ALERT type 2 Indicator that the structure model may be wrong or deficient
13 ALERT type 3 Indicator that the structure quality may be low
29 ALERT type 4 Improvement, methodology, query or suggestion
3 ALERT type 5 Informative message, check

It is advisable to attempt to resolve as many as possible of the alerts in all categories. Often the minor alerts point to easily fixed oversights, errors and omissions in your CIF or refinement strategy, so attention to these fine details can be worthwhile. In order to resolve some of the more serious problems it may be necessary to carry out additional measurements or structure refinements. However, the purpose of your study may justify the reported deviations and the more serious of these should normally be commented upon in the discussion or experimental section of a paper or in the "special_details" fields of the CIF. checkCIF was carefully designed to identify outliers and unusual parameters, but every test has its limitations and alerts that are not important in a particular case may appear. Conversely, the absence of alerts does not guarantee there are no aspects of the results needing attention. It is up to the individual to critically assess their own results and, if necessary, seek expert advice.

Publication of your CIF in IUCr journals

A basic structural check has been run on your CIF. These basic checks will be run on all CIFs submitted for publication in IUCr journals (*Acta Crystallographica*, *Journal of Applied Crystallography*, *Journal of Synchrotron Radiation*); however, if you intend to submit to *Acta Crystallographica Section C* or *E* or *IUCrData*, you should make sure that full publication checks are run on the final version of your CIF prior to submission.

Publication of your CIF in other journals

Please refer to the *Notes for Authors* of the relevant journal for any special instructions relating to CIF submission.

PLATON version of 11/11/2024; check.def file version of 11/11/2024

Datablock fx449_auto_sq - ellipsoid plot

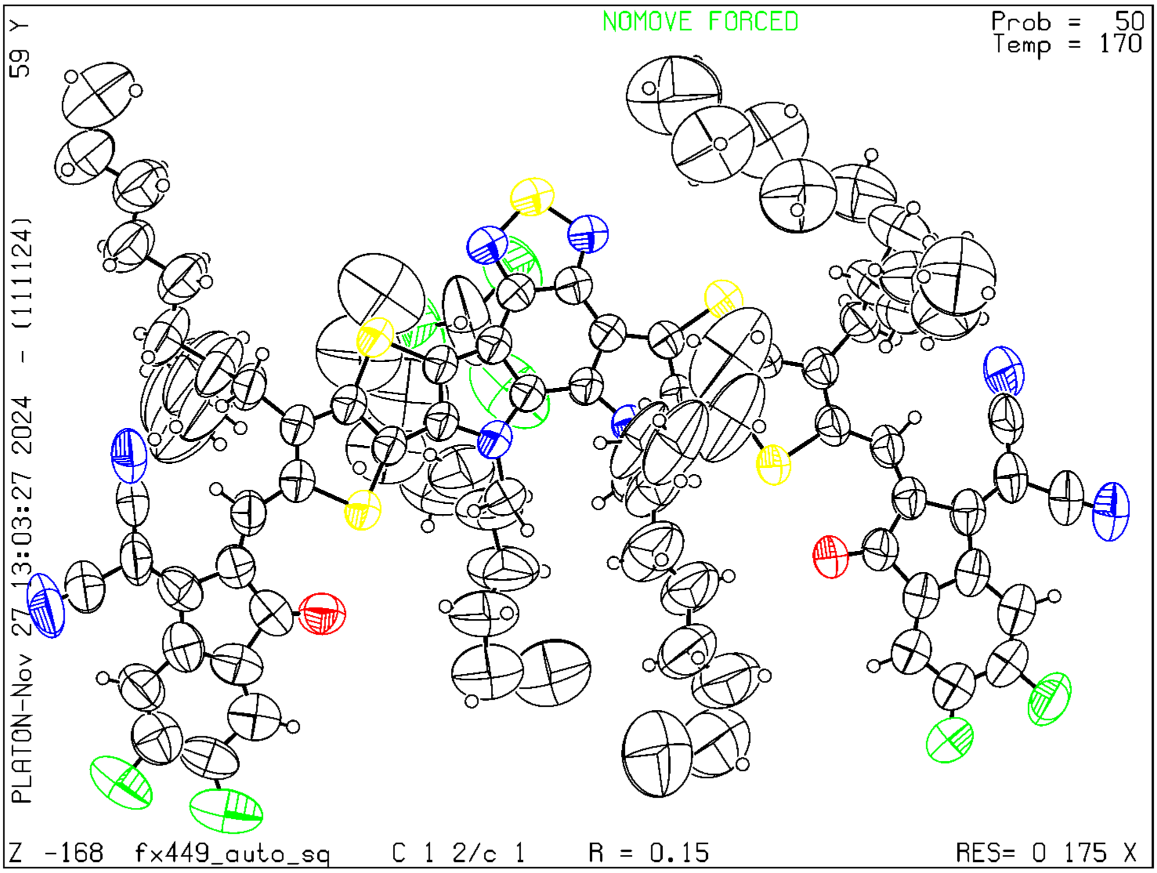

Supplement: Supplementary file 17 — Supplementary Data 15 [file 41467_2025_56799_MOESM17_ESM.pdf]
