# Supplementary material for: Asymmetric small-molecule acceptor enables suppressed electron-vibration coupling and minimized driving force for organic solar cells
Source: Nat Commun. 2025 Feb 10;16:1503. doi: 10.1038/s41467-025-56799-6 (PMC11811148; doi:10.1038/s41467-025-56799-6)

checkCIF/PLATON report

Structure factors have been supplied for datablock(s) fx651_sq

THIS REPORT IS FOR GUIDANCE ONLY. IF USED AS PART OF A REVIEW PROCEDURE FOR PUBLICATION, IT SHOULD NOT REPLACE THE EXPERTISE OF AN EXPERIENCED CRYSTALLOGRAPHIC REFEREE.

No syntax errors found. CIF dictionary Interpreting this report

Datablock: fx651_sq

Bond precision: C-C = 0.0215 A Wavelength=1.54184

Cell:	a=27.5504(13)	b=57.0641(14)	c=13.5264(5)
	alpha=90	beta=93.431(4)	gamma=90
Temperature:	170 K		

	Calculated	Reported
Volume	21227.3(14)	21227.3(14)
Space group	C 2/c	C 1 2/c 1
Hall group	-C 2yc	-C 2yc
Moiety formula	C87 H56 F4 N8 O2 S7 [+ solvent]	0.034(C88 H82 F4 N8 O2 S7), 0[]
Sum formula	C87 H56 F4 N8 O2 S7 [+ solvent]	C87 H56 F4 N8 O2 S7
Mr	1545.82	1545.81
Dx,g cm-3	0.967	0.967
Z	8	8
Mu (mm-1)	1.757	1.757
F000	6384.0	6384.0
F000'	6419.52	
h,k,lmax	33,68,16	33,68,16
Nref	19342	18860
Tmin,Tmax	0.765,0.949	0.479,1.000
Tmin'	0.659	

Correction method= # Reported T Limits: Tmin=0.479 Tmax=1.000
AbsCorr = MULTI-SCAN

Data completeness= 0.975 Theta(max)= 67.999

```
R(reflections)= 0.1786( 10285)          wR2(reflections)=
                                        0.3481( 18860)
S = 1.284                  Npar= 973
```

The following ALERTS were generated. Each ALERT has the format
test-name_ALERT_alert-type_alert-level.
Click on the hyperlinks for more details of the test.

Alert level B

```
PLAT082_ALERT_2_B High R1 Value ................................      0.18 Report
PLAT220_ALERT_2_B NonSolvent   Resd 1  C   Ueq(max)/Ueq(min) Range    7.5 Ratio
PLAT315_ALERT_2_B Singly Bonded Carbon Detected (H-atoms Missing).    C52 Check
PLAT315_ALERT_2_B Singly Bonded Carbon Detected (H-atoms Missing).    C56 Check
PLAT315_ALERT_2_B Singly Bonded Carbon Detected (H-atoms Missing).    C63 Check
PLAT315_ALERT_2_B Singly Bonded Carbon Detected (H-atoms Missing).    C67 Check
PLAT315_ALERT_2_B Singly Bonded Carbon Detected (H-atoms Missing).    C73 Check
PLAT315_ALERT_2_B Singly Bonded Carbon Detected (H-atoms Missing).    C77 Check
PLAT315_ALERT_2_B Singly Bonded Carbon Detected (H-atoms Missing).    C85 Check
PLAT315_ALERT_2_B Singly Bonded Carbon Detected (H-atoms Missing).    C87 Check
PLAT340_ALERT_3_B Low Bond Precision on  C-C Bonds ..............  0.02152 Ang.
```

Alert level C

```
PLAT029_ALERT_3_C _diffrn_measured_fraction_theta_full value Low .   0.976 Why?
PLAT084_ALERT_3_C High wR2 Value (i.e. > 0.25) ..................     0.35 Report
PLAT220_ALERT_2_C NonSolvent   Resd 1  S   Ueq(max)/Ueq(min) Range    3.4 Ratio
PLAT222_ALERT_3_C NonSolvent Resd 1  H   Uiso(max)/Uiso(min) Range    5.4 Ratio
PLAT230_ALERT_2_C Hirshfeld Test Diff for    O2       --C34      .    5.4 s.u.
PLAT230_ALERT_2_C Hirshfeld Test Diff for    C20      --C27      .    5.5 s.u.
PLAT230_ALERT_2_C Hirshfeld Test Diff for    C47      --C48      .    5.7 s.u.
PLAT234_ALERT_4_C Large Hirshfeld Difference S5       --C28      .   0.16 Ang.
PLAT234_ALERT_4_C Large Hirshfeld Difference S7       --C78      .   0.19 Ang.
PLAT234_ALERT_4_C Large Hirshfeld Difference S7       --C81      .   0.16 Ang.
PLAT234_ALERT_4_C Large Hirshfeld Difference F3       --C37      .   0.16 Ang.
PLAT234_ALERT_4_C Large Hirshfeld Difference C2       --C10      .   0.16 Ang.
PLAT234_ALERT_4_C Large Hirshfeld Difference C4       --C5       .   0.18 Ang.
PLAT234_ALERT_4_C Large Hirshfeld Difference C5       --C6       .   0.23 Ang.
PLAT234_ALERT_4_C Large Hirshfeld Difference C19      --C20      .   0.16 Ang.
PLAT234_ALERT_4_C Large Hirshfeld Difference C22      --C23      .   0.19 Ang.
PLAT234_ALERT_4_C Large Hirshfeld Difference C30      --C31      .   0.21 Ang.
PLAT234_ALERT_4_C Large Hirshfeld Difference C46      --C47      .   0.17 Ang.
PLAT234_ALERT_4_C Large Hirshfeld Difference C49      --C50      .   0.23 Ang.
PLAT234_ALERT_4_C Large Hirshfeld Difference C50      --C51      .   0.24 Ang.
PLAT234_ALERT_4_C Large Hirshfeld Difference C57      --C58      .   0.16 Ang.
PLAT234_ALERT_4_C Large Hirshfeld Difference C59      --C60      .   0.18 Ang.
PLAT234_ALERT_4_C Large Hirshfeld Difference C68      --C69      .   0.18 Ang.
PLAT234_ALERT_4_C Large Hirshfeld Difference C72      --C73      .   0.22 Ang.
PLAT234_ALERT_4_C Large Hirshfeld Difference C79      --C80      .   0.21 Ang.
PLAT234_ALERT_4_C Large Hirshfeld Difference C80      --C82      .   0.22 Ang.
PLAT234_ALERT_4_C Large Hirshfeld Difference C82      --C83      .   0.25 Ang.
PLAT241_ALERT_2_C High   'MainMol' Ueq as Compared to Neighbors of     S4 Check
PLAT241_ALERT_2_C High   'MainMol' Ueq as Compared to Neighbors of     S6 Check
PLAT241_ALERT_2_C High   'MainMol' Ueq as Compared to Neighbors of     S7 Check
PLAT241_ALERT_2_C High   'MainMol' Ueq as Compared to Neighbors of    C49 Check
```

```
PLAT242_ALERT_2_C Low    'MainMol' Ueq as Compared to Neighbors of        C29 Check
PLAT242_ALERT_2_C Low    'MainMol' Ueq as Compared to Neighbors of        C47 Check
PLAT242_ALERT_2_C Low    'MainMol' Ueq as Compared to Neighbors of        C62 Check
PLAT242_ALERT_2_C Low    'MainMol' Ueq as Compared to Neighbors of        C72 Check
PLAT242_ALERT_2_C Low    'MainMol' Ueq as Compared to Neighbors of        C79 Check
PLAT260_ALERT_2_C Large Average Ueq of Residue Including        S1      0.151 Check
PLAT369_ALERT_2_C Long   C(sp2)-C(sp2) Bond  C30      - C78      .       1.54 Ang.
PLAT410_ALERT_2_C Short Intra H...H Contact  H57A     ..H68A     .       1.94 Ang.
                                                      x,y,z  =      1_555 Check
PLAT906_ALERT_3_C Large K Value in the Analysis of Variance ......      8.055 Check
PLAT906_ALERT_3_C Large K Value in the Analysis of Variance ......      3.744 Check
PLAT906_ALERT_3_C Large K Value in the Analysis of Variance ......      2.440 Check
PLAT911_ALERT_3_C Missing FCF Refl Between Thmin & STh/L=    0.600        463 Report
               26  0  0,  17  1  0,   0  8  0,   0 10  0,   2 12  0,   1 13  0,
                0 14  0,   2 18  0,   0 20  0,   7 27  0,   6 28  0,  27 35  0,
               26 36  0,  28 36  0,  27 37  0,  25 39  0,  19 47  0,  17 49  0,
               14 62  0,   0 64  0,   4 64  0,   1 65  0, -33  1  1,  -3  1  1,
               -5  3  1,  -3  5  1,   2  8  1,  -1  9  1,  -4 12  1,   4 12  1,
                0 14  1,   2 14  1,  -5 15  1,  -6 16  1,  21 29  1, -23 33  1,
              -26 34  1, -27 35  1,  27 35  1, -28 36  1, -26 36  1,  26 36  1,
               27 37  1,  24 40  1,  24 42  1, -22 46  1,  14 60  1,   1 63  1,
                2 64  1,   4 64  1,   3 65  1,   2 66  1,   4 66  1,   8 66  1,
               -9  1  2,  27  1  2,  32  2  2,  -4  8  2,   1  9  2,  -6 20  2,
               30 24  2, -27 29  2, -26 30  2, -27 31  2,  25 31  2, -26 32  2,
               18 32  2,  25 33  2,  18 34  2,  17 35  2,  27 35  2,  16 36  2,
               26 36  2,  27 37  2,   4 38  2,  24 42  2,   4 66  2,   6 66  2,
                3 67  2,   5 67  2,  11  1  3,  27  1  3,  -2  2  3,  32  2  3,
                1  3  3,  27  3  3,  26  4  3,  27  5  3, -26 16  3,  -7 21  3,
               30 22  3,  -8 24  3,   4 24  3,  -9 25  3,  25 31  3, -15 35  3,
PLAT913_ALERT_3_C Missing # of Very Strong Reflections in FCF ....          4 Note
                1  1  0,   1  3  3,  -2  0  4,   0  0  4,
PLAT918_ALERT_3_C Reflection(s) with I(obs) much Smaller I(calc) .          6 Check
```

Alert level G

```
FORMU01_ALERT_1_G  There is a discrepancy between the atom counts in the
            _chemical_formula_sum and _chemical_formula_moiety. This is
            usually due to the moiety formula being in the wrong format.
            Atom count from _chemical_formula_sum:   C87 H56 F4 N8 O2 S7
            Atom count from _chemical_formula_moiety:C2.992 H2.788 F0.136 N0.272 O
PLAT002_ALERT_2_G Number of Distance or Angle Restraints on AtSite         43 Note
PLAT003_ALERT_2_G Number of Uiso or U(i,j)  Restrained non-H-Atoms         58 Report
PLAT042_ALERT_1_G Calc. and Reported MoietyFormula Strings  Differ     Please Check
              Calc: C87 H56 F4 N8 O2 S7
              Rep.: 0.034(C88 H82 F4 N8 O2 S7), 0[]
PLAT083_ALERT_2_G SHELXL Second Parameter in WGHT  Unusually Large     211.46 Why ?
PLAT172_ALERT_4_G The CIF-Embedded .res File Contains DFIX Records          7 Report
PLAT173_ALERT_4_G The CIF-Embedded .res File Contains DANG Records          3 Report
PLAT177_ALERT_4_G The CIF-Embedded .res File Contains DELU Records          1 Report
PLAT178_ALERT_4_G The CIF-Embedded .res File Contains SIMU Records          5 Report
PLAT186_ALERT_4_G The CIF-Embedded .res File Contains ISOR Records          1 Report
PLAT188_ALERT_3_G A Non-default SIMU Restraint Value has been used     0.0100 Report
PLAT188_ALERT_3_G A Non-default SIMU Restraint Value has been used     0.0100 Report
PLAT188_ALERT_3_G A Non-default SIMU Restraint Value has been used     0.0100 Report
PLAT188_ALERT_3_G A Non-default SIMU Restraint Value has been used     0.0100 Report
PLAT188_ALERT_3_G A Non-default SIMU Restraint Value has been used     0.0100 Report
PLAT333_ALERT_2_G Large Aver C6-Ring C-C Dist C20      -C27      .       1.43 Ang.
```

```
PLAT335_ALERT_2_G Check Large C6 Ring C-C Range C35      -C40           0.19 Ang.
PLAT343_ALERT_2_G Unusual sp3      Angle Range in Main Residue for        C51 Check
PLAT343_ALERT_2_G Unusual sp?      Angle Range in Main Residue for        C52 Check
PLAT343_ALERT_2_G Unusual sp?      Angle Range in Main Residue for        C54 Check
PLAT343_ALERT_2_G Unusual sp?      Angle Range in Main Residue for        C55 Check
PLAT343_ALERT_2_G Unusual sp?      Angle Range in Main Residue for        C56 Check
PLAT343_ALERT_2_G Unusual sp?      Angle Range in Main Residue for        C63 Check
PLAT343_ALERT_2_G Unusual sp?      Angle Range in Main Residue for        C67 Check
PLAT343_ALERT_2_G Unusual sp?      Angle Range in Main Residue for        C73 Check
PLAT343_ALERT_2_G Unusual sp?      Angle Range in Main Residue for        C77 Check
PLAT343_ALERT_2_G Unusual sp3      Angle Range in Main Residue for        C83 Check
PLAT343_ALERT_2_G Unusual sp3      Angle Range in Main Residue for        C84 Check
PLAT343_ALERT_2_G Unusual sp?      Angle Range in Main Residue for        C85 Check
PLAT343_ALERT_2_G Unusual sp3      Angle Range in Main Residue for        C86 Check
PLAT343_ALERT_2_G Unusual sp?      Angle Range in Main Residue for        C87 Check
PLAT367_ALERT_2_G Long?  C(sp?)-C(sp?) Bond  C51      - C52      .       1.55 Ang.
PLAT367_ALERT_2_G Long?  C(sp?)-C(sp?) Bond  C53      - C54      .       1.56 Ang.
PLAT367_ALERT_2_G Long?  C(sp?)-C(sp?) Bond  C54      - C55      .       1.54 Ang.
PLAT367_ALERT_2_G Long?  C(sp?)-C(sp?) Bond  C55      - C56      .       1.55 Ang.
PLAT367_ALERT_2_G Long?  C(sp?)-C(sp?) Bond  C62      - C63      .       1.56 Ang.
PLAT367_ALERT_2_G Long?  C(sp?)-C(sp?) Bond  C66      - C67      .       1.55 Ang.
PLAT367_ALERT_2_G Long?  C(sp?)-C(sp?) Bond  C72      - C73      .       1.56 Ang.
PLAT367_ALERT_2_G Long?  C(sp?)-C(sp?) Bond  C76      - C77      .       1.54 Ang.
PLAT367_ALERT_2_G Long?  C(sp?)-C(sp?) Bond  C84      - C85      .       1.56 Ang.
PLAT367_ALERT_2_G Long?  C(sp?)-C(sp?) Bond  C86      - C87      .       1.57 Ang.
PLAT606_ALERT_4_G Solvent Accessible VOID(S) in Structure ........         ! Info
PLAT793_ALERT_4_G Model has Chirality at C50         (Centro SpGr)         R Verify
PLAT793_ALERT_4_G Model has Chirality at C58         (Centro SpGr)         S Verify
PLAT860_ALERT_3_G Number of Least-Squares Restraints ............        405 Note
PLAT869_ALERT_4_G ALERTS Related to the Use of SQUEEZE  Suppressed         ! Info
PLAT909_ALERT_3_G Percentage of I>2sig(I) Data at Theta(Max) Still        32% Note
PLAT910_ALERT_3_G Missing # of FCF Reflection(s) Below Theta(Min).         3 Note
                1  1  0,   0  2  0,   1  3  0,
PLAT912_ALERT_4_G Missing # of FCF Reflections Above STh/L=  0.600        15 Note
PLAT933_ALERT_2_G Number of HKL-OMIT Records in Embedded .res File        23 Note
               -5  3  1,  -4  8  2,  -3  1  1,  -3  5  1,  -2  0  4,  -2  2  3,
               -1  1  4,  -1  9  1,   0  0  4,   0  2  4,   0  8  0,   0 10  0,
                0 14  0,   0 14  1,   0 20  0,   1  3  3,   1  9  2,   1 13  0,
                2  8  1,   2 12  0,   2 14  1,   2 18  0,   4 12  1,
PLAT941_ALERT_3_G Average HKL Measurement Multiplicity ..........        3.4 Low
PLAT967_ALERT_5_G Note: Two-Theta Cutoff Value in Embedded .res ..      136.0 Degree
PLAT969_ALERT_5_G The 'Henn et al.' R-Factor-gap value ..........      7.753 Note
              Predicted wR2: Based on SigI**2  4.49 or SHELX Weight 27.28
PLAT978_ALERT_2_G Number C-C Bonds with Positive Residual Density.         0 Info
PLAT992_ALERT_5_G Repd & Actual _reflns_number_gt Values Differ by         2 Check
```

---

 0 **ALERT level A** = Most likely a serious problem - resolve or explain
11 **ALERT level B** = A potentially serious problem, consider carefully
45 **ALERT level C** = Check. Ensure it is not caused by an omission or oversight
55 **ALERT level G** = General information/check it is not something unexpected

 2 ALERT type 1 CIF construction/syntax error, inconsistent or missing data
57 ALERT type 2 Indicator that the structure model may be wrong or deficient
19 ALERT type 3 Indicator that the structure quality may be low
30 ALERT type 4 Improvement, methodology, query or suggestion
 3 ALERT type 5 Informative message, check

It is advisable to attempt to resolve as many as possible of the alerts in all categories. Often the minor alerts point to easily fixed oversights, errors and omissions in your CIF or refinement strategy, so attention to these fine details can be worthwhile. In order to resolve some of the more serious problems it may be necessary to carry out additional measurements or structure refinements. However, the purpose of your study may justify the reported deviations and the more serious of these should normally be commented upon in the discussion or experimental section of a paper or in the "special_details" fields of the CIF. checkCIF was carefully designed to identify outliers and unusual parameters, but every test has its limitations and alerts that are not important in a particular case may appear. Conversely, the absence of alerts does not guarantee there are no aspects of the results needing attention. It is up to the individual to critically assess their own results and, if necessary, seek expert advice.

Publication of your CIF in IUCr journals

A basic structural check has been run on your CIF. These basic checks will be run on all CIFs submitted for publication in IUCr journals (*Acta Crystallographica*, *Journal of Applied Crystallography*, *Journal of Synchrotron Radiation*); however, if you intend to submit to *Acta Crystallographica Section C* or *E* or *IUCrData*, you should make sure that full publication checks are run on the final version of your CIF prior to submission.

Publication of your CIF in other journals

Please refer to the *Notes for Authors* of the relevant journal for any special instructions relating to CIF submission.

PLATON version of 11/11/2024; check.def file version of 11/11/2024

Datablock fx651_sq - ellipsoid plot

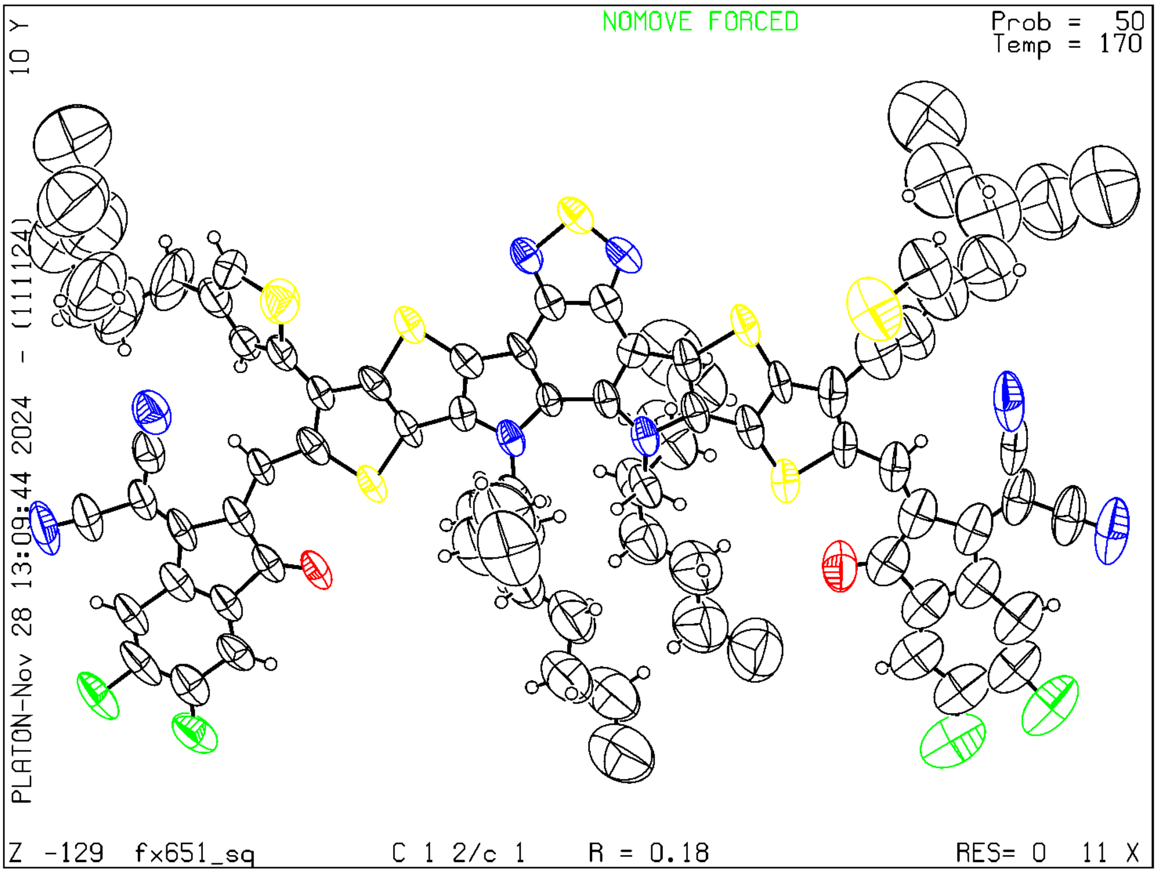

Supplement: Supplementary file 18 — Supplementary Data 16 [file 41467_2025_56799_MOESM18_ESM.pdf]
