# Supplementary material for: Asymmetric small-molecule acceptor enables suppressed electron-vibration coupling and minimized driving force for organic solar cells
Source: Nat Commun. 2025 Feb 10;16:1503. doi: 10.1038/s41467-025-56799-6 (PMC11811148; doi:10.1038/s41467-025-56799-6)
Supplement: Supplementary file 19 — Reporting Summary [file 41467_2025_56799_MOESM19_ESM.pdf]

nature portfolio

Corresponding author(s): Beibei Qiu

# Solar Cells Reporting Summary

Nature Portfolio wishes to improve the reproducibility of the work that we publish. This form is intended for publication with all accepted papers reporting the characterization of photovoltaic devices and provides structure for consistency and transparency in reporting. Some list items might not apply to an individual manuscript, but all fields must be completed for clarity.

For further information on Nature Research policies, including our data availability policy, see Authors & Referees.

## Experimental design

**Please check the following details are reported in the manuscript, and provide a brief description or explanation where applicable.**

### 1. Dimensions

Area of the tested solar cells

- [x] Yes — The effective area of the OSCs is 6 mm2.
- [ ] No — *Explain why this information is not reported/not relevant.*

Method used to determine the device area

- [x] Yes — The device area is calibrated using an optical microscope. And the statement can be found in the 'Method' part in the manuscript.
- [ ] No — *Explain why this information is not reported/not relevant.*

### 2. Current-voltage characterization

Current density-voltage (J-V) plots in both forward and backward direction

- [ ] Yes — Only forward direction was tested, as organic solar cells have no hysteresis effect.
- [x] No

Voltage scan conditions

- [x] Yes — The J-V curves are measured in the forward direction from −0.2 to 1.0 V
- [ ] No — *Explain why this information is not reported/not relevant.*

Test environment

- [x] Yes — The current density-voltage (J-V) curves of devices were measured in a glove box filled with nitrogen (O2 < 10 ppm; H2O < 10 ppm).
- [ ] No — *Explain why this information is not reported/not relevant.*

Protocol for preconditioning of the device before its characterization

- [ ] Yes — *Provide a description of the protocol.*
- [x] No — No preconditioning was used before characterization.

Stability of the J-V characteristic

- [x] Yes — The maximum power point tracking stability (measured in glove box under AM 1.5 G spectra) has been measured.
- [ ] No — *Explain why this information is not reported/not relevant.*

### 3. Hysteresis or any other unusual behaviour

Description of the unusual behaviour observed during the characterization

- [ ] Yes — *Provide a description of hysteresis or any other unusual behaviour observed during the characterization.*
- [x] No — Organic solar cells have no hysteresis effect.

Related experimental data

- [ ] Yes — *Provide a description of the related experimental data.*
- [x] No — Organic solar cells have no hysteresis effect.

### 4. Efficiency

External quantum efficiency (EQE) or incident photons to current efficiency (IPCE)

- [x] Yes — External quantum efficiency (EQE) was measured by the solar cell spectral response measurement system QE-R3011 (Taiwan Enli Technology Co., Ltd.).
- [ ] No — *Explain why this information is not reported/not relevant.*

A comparison between the integrated response under the standard reference spectrum and the response measure under the simulator

- [x] Yes — Table 2 in the manuscript displays the information.
- [ ] No — *Explain why this information is not reported/not relevant.*



April 2023

For tandem solar cells, the bias illumination and bias voltage used for each subcell

☐ Yes — *Provide a description of the measurement conditions.*
☒ No — Tandem solar cells were not fabricated.

## 5. Calibration

Light source and reference cell or sensor used for the characterization

☒ Yes — The J–V curves of OSCs were tested by a Keysight B2901BL source meter and an AAA grade solar simulator (SS-X50, Enli Tech. Co., Ltd., Taiwan) along with AM 1.5 G spectra corrected by a standard silicon solar cell at 1000 W/m2.
☐ No — *Explain why this information is not reported/not relevant.*

Confirmation that the reference cell was calibrated and certified

☒ Yes — The reference cell was calibrated and certified by Enli Technology Co., Ltd.
☐ No — *Explain why this information is not reported/not relevant.*

Calculation of spectral mismatch between the reference cell and the devices under test

☐ Yes — *Provide a value of the spectral mismatch and/or a description of how it has been taken into account in the measurements.*
☒ No — The mismatch has not been measured.

## 6. Mask/aperture

Size of the mask/aperture used during testing

☐ Yes — *Report the size of the mask/aperture.*
☒ No — No mask is used to test the efficiency of the devices.

Variation of the measured short-circuit current density with the mask/aperture area

☐ Yes — *Report the difference in the short-circuit current density values measured with the mask and aperture area.*
☒ No — No mask is used to test the efficiency of the devices.

## 7. Performance certification

Identity of the independent certification laboratory that confirmed the photovoltaic performance

☐ Yes — *Identify the independent certification laboratory.*
☒ No — The efficiency has not been certified by independent certification laboratory.

A copy of any certificate(s)

☐ Yes — *Certificate copies should be provided in the Supplementary information. Please state the supplementary item number.*
☒ No — The efficiency has not been certified by independent certification laboratory.

## 8. Statistics

Number of solar cells tested

☒ Yes — 20 devices have been tested.
☐ No — *Explain why this information is not reported/not relevant.*

Statistical analysis of the device performance

☒ Yes — Table 2 in the manuscript displays the statistic analysis information.
☐ No — *Explain why this information is not reported/not relevant.*

## 9. Long-term stability analysis

Type of analysis, bias conditions and environmental conditions

☒ Yes — Photo-stability (measured in glove box under white LED light for 250 h) and storage stability (measured in glove box for 1600 h) have been measured.
☐ No — *Explain why this information is not reported/not relevant.*
